# Supplementary figures and images for: The effect of lidocaine against sepsis-induced acute lung injury in a mouse model through the JAK2/STAT3 pathway
Source: PLoS One. 2025 May 8;20(5):e0322653. doi: 10.1371/journal.pone.0322653 (PMC12061136; doi:10.1371/journal.pone.0322653)

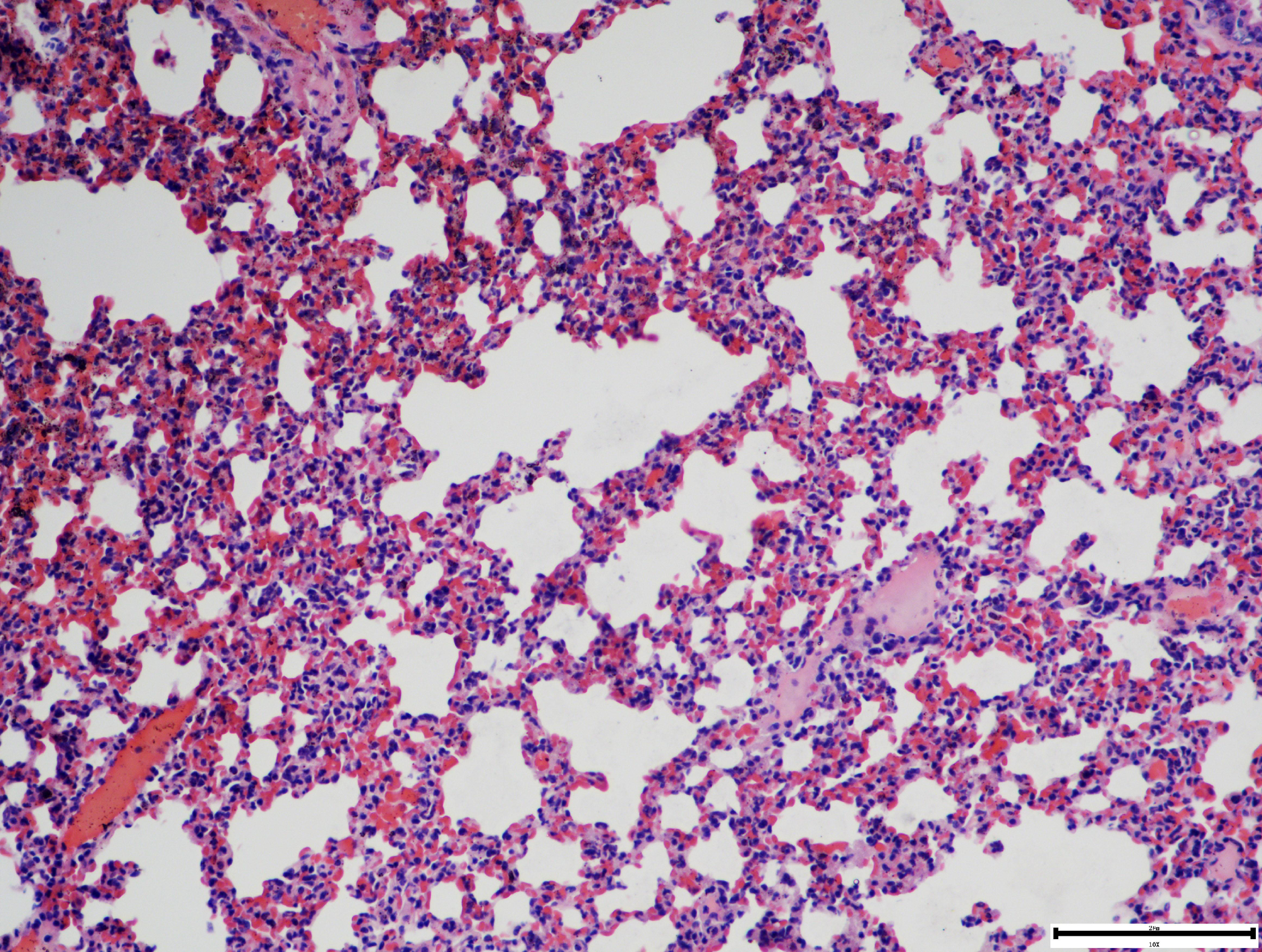

Supplement: S1 Raw Images — (ZIP) [file pone.0322653.s001.zip › S1_raw_images1-tunel and HE picture/CLP10 200X (2).tif]

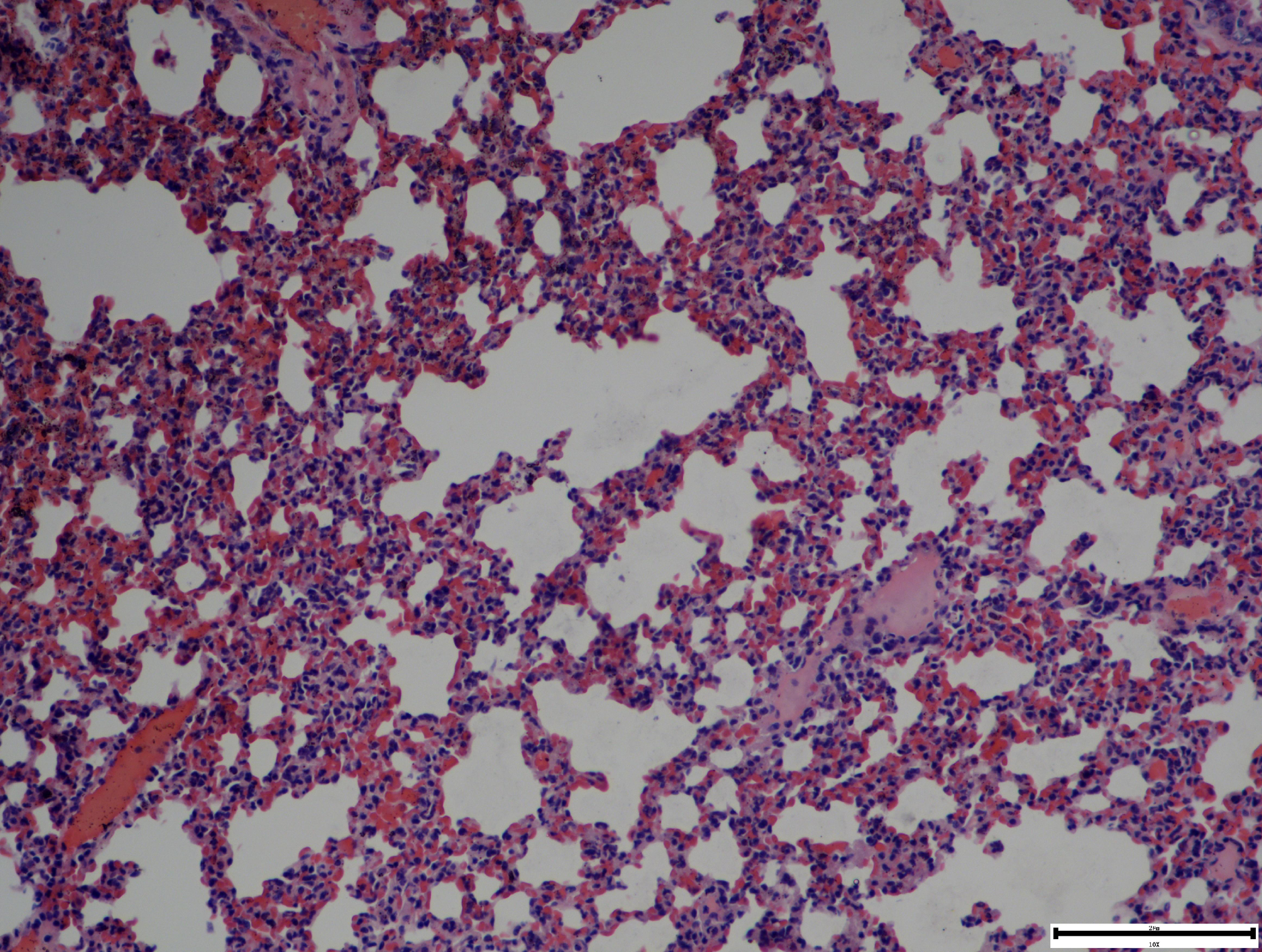

Supplement: S1 Raw Images — (ZIP) [file pone.0322653.s001.zip › S1_raw_images1-tunel and HE picture/CLP10 200X.tif]

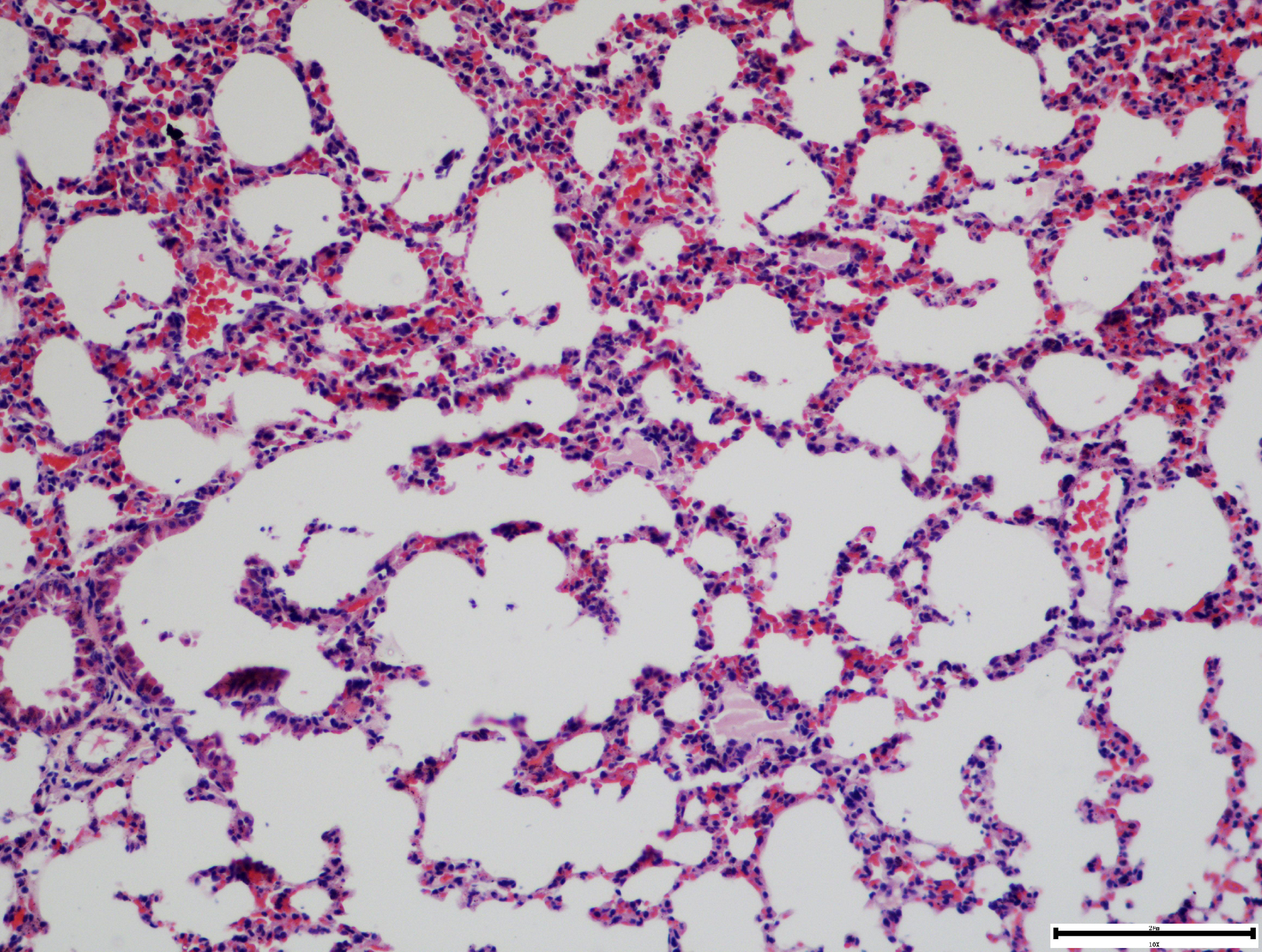

Supplement: S1 Raw Images — (ZIP) [file pone.0322653.s001.zip › S1_raw_images1-tunel and HE picture/LD3 200X (2).tif]

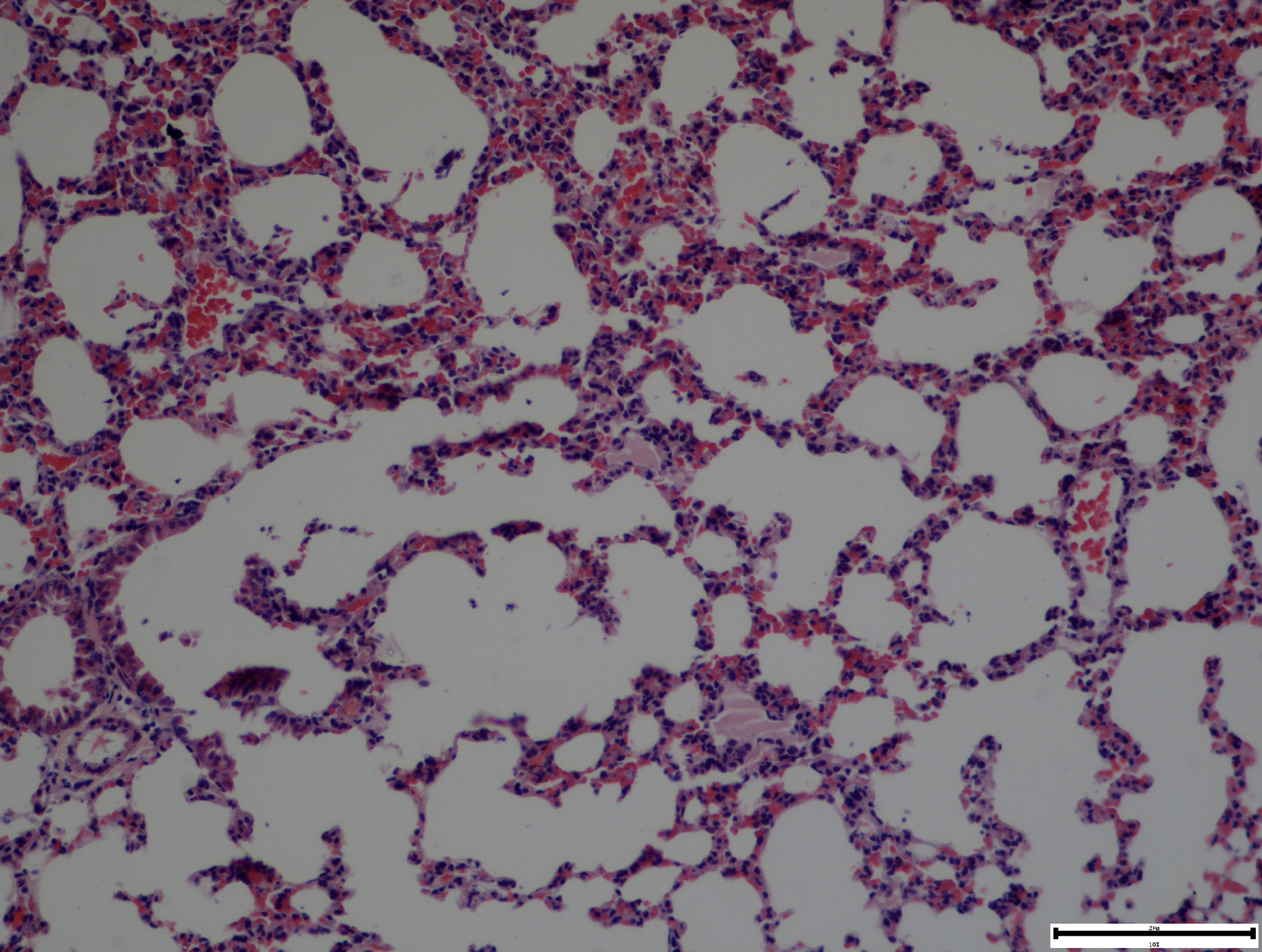

Supplement: S1 Raw Images — (ZIP) [file pone.0322653.s001.zip › S1_raw_images1-tunel and HE picture/LD3 200X.tif]

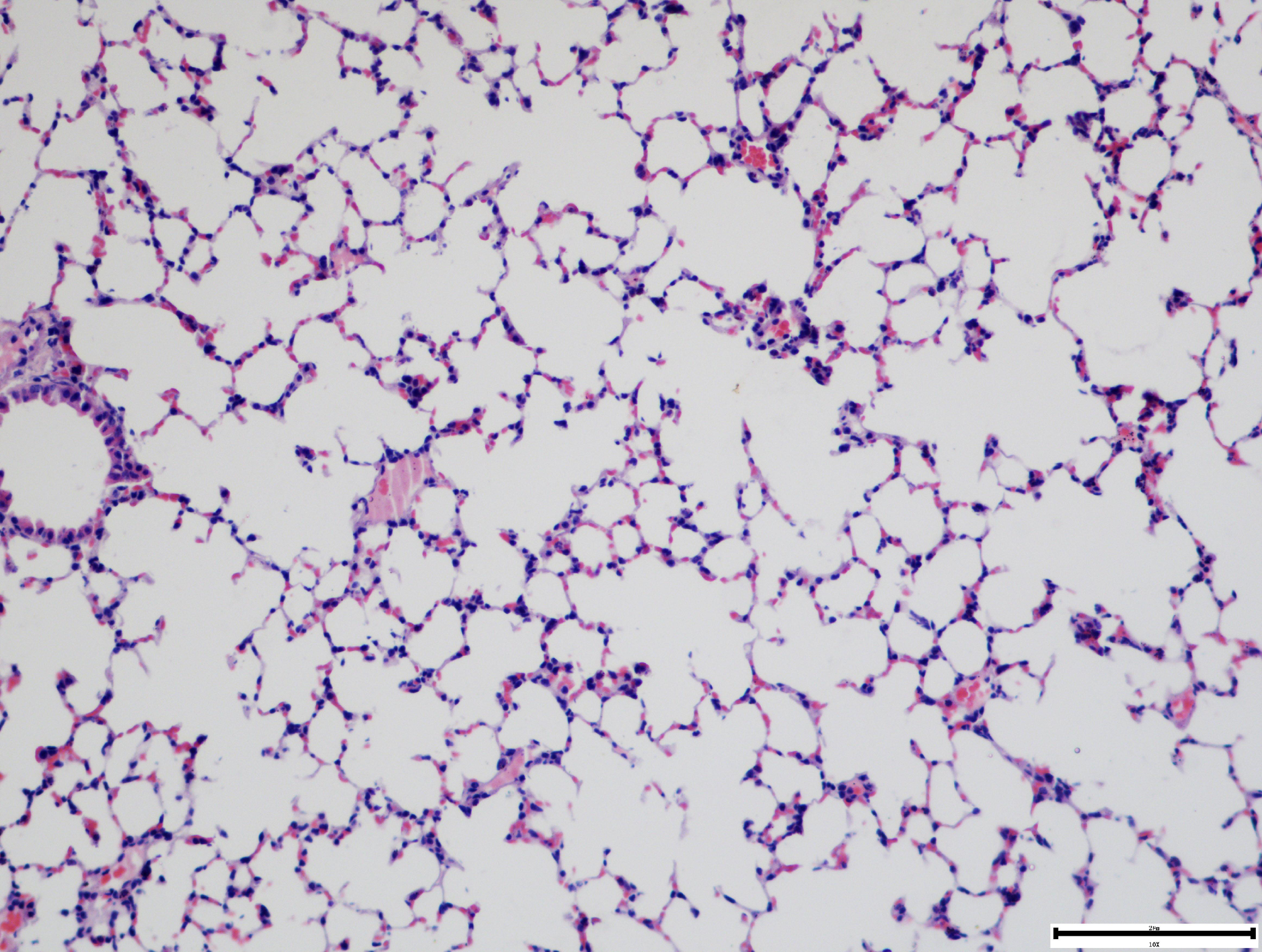

Supplement: S1 Raw Images — (ZIP) [file pone.0322653.s001.zip › S1_raw_images1-tunel and HE picture/S4 200x (2).tif]

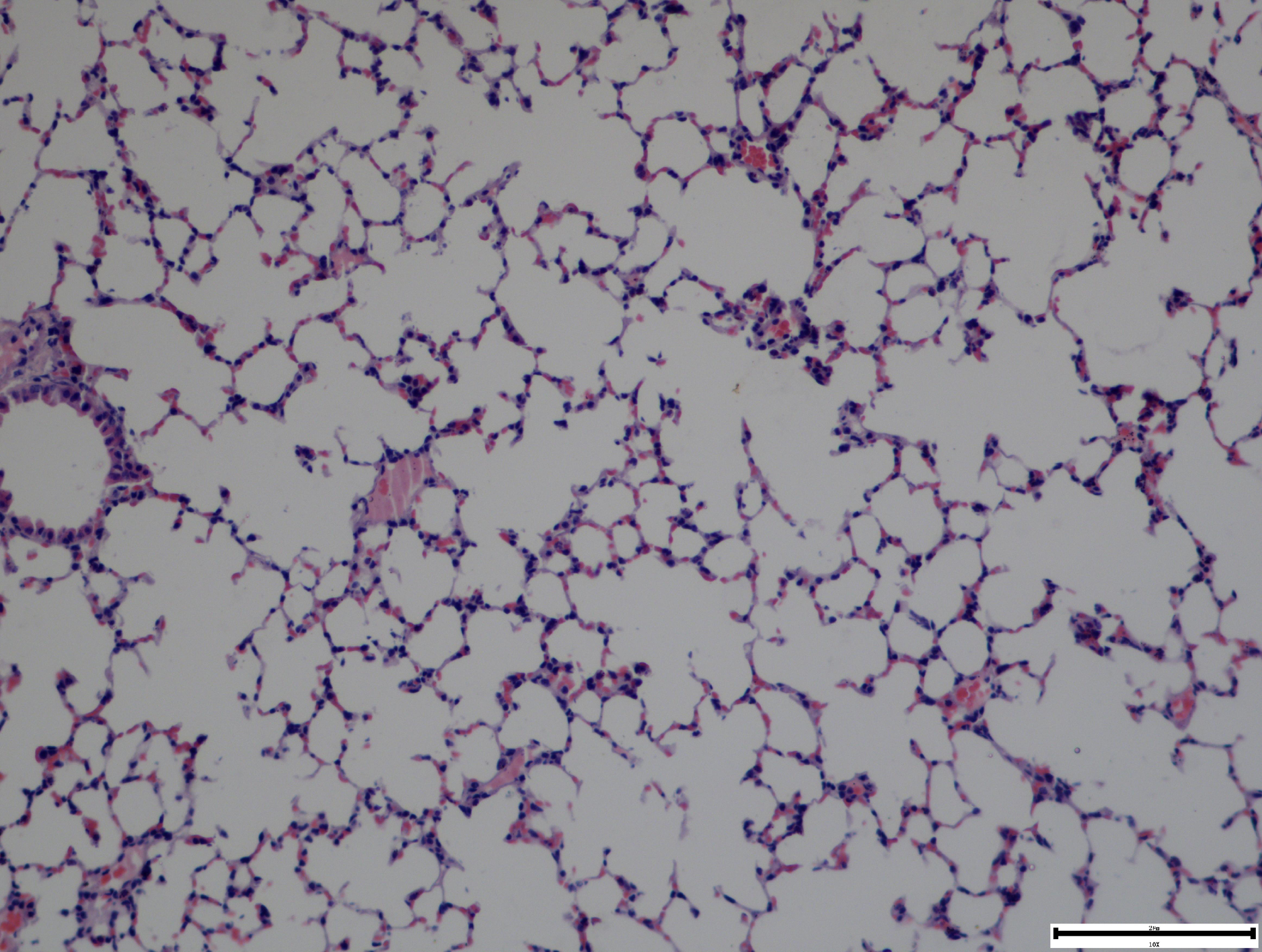

Supplement: S1 Raw Images — (ZIP) [file pone.0322653.s001.zip › S1_raw_images1-tunel and HE picture/S4 200x.tif]

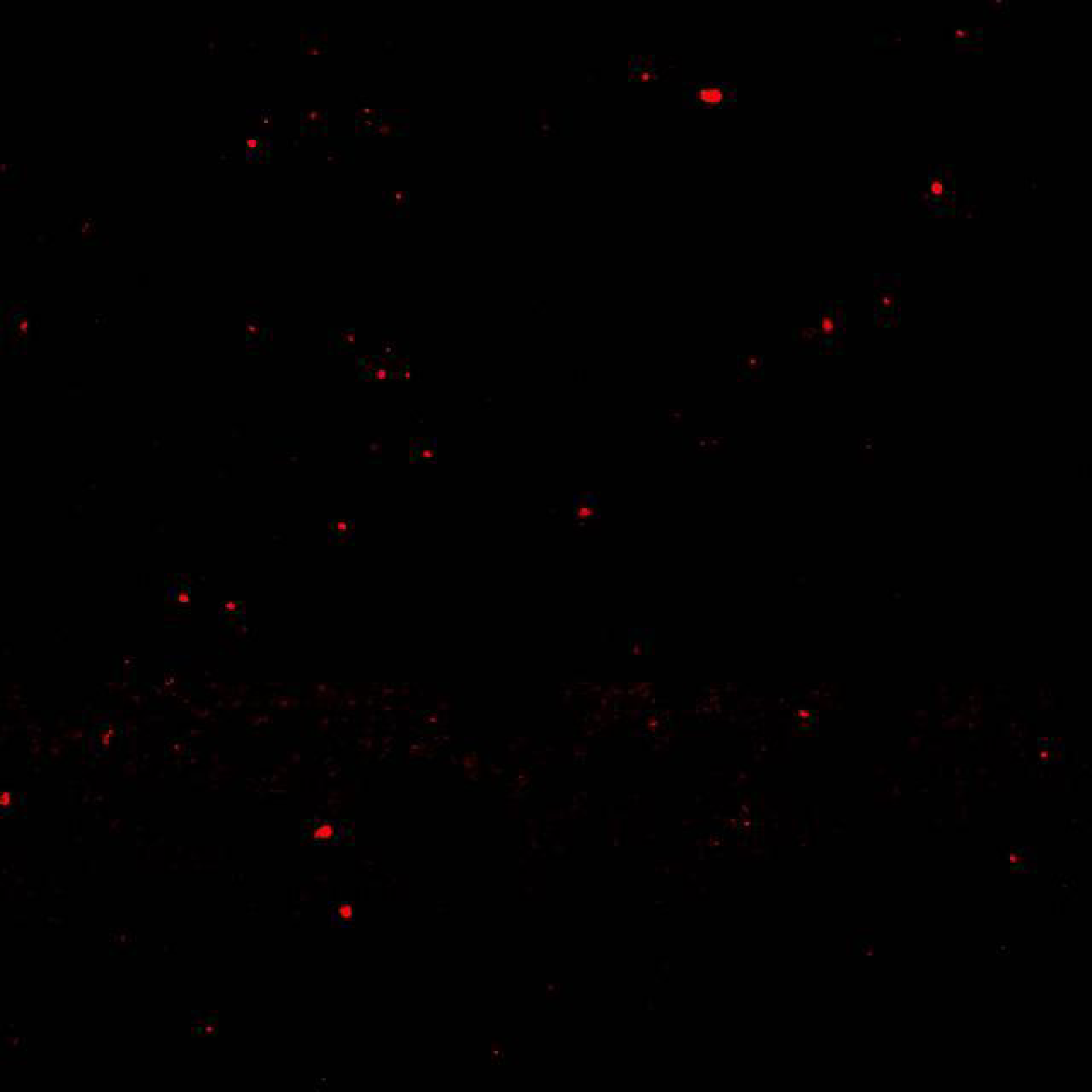

Supplement: S1 Raw Images — (ZIP) [file pone.0322653.s001.zip › S1_raw_images1-tunel and HE picture/TUNEL/CA4-002 20X cy3.tif]

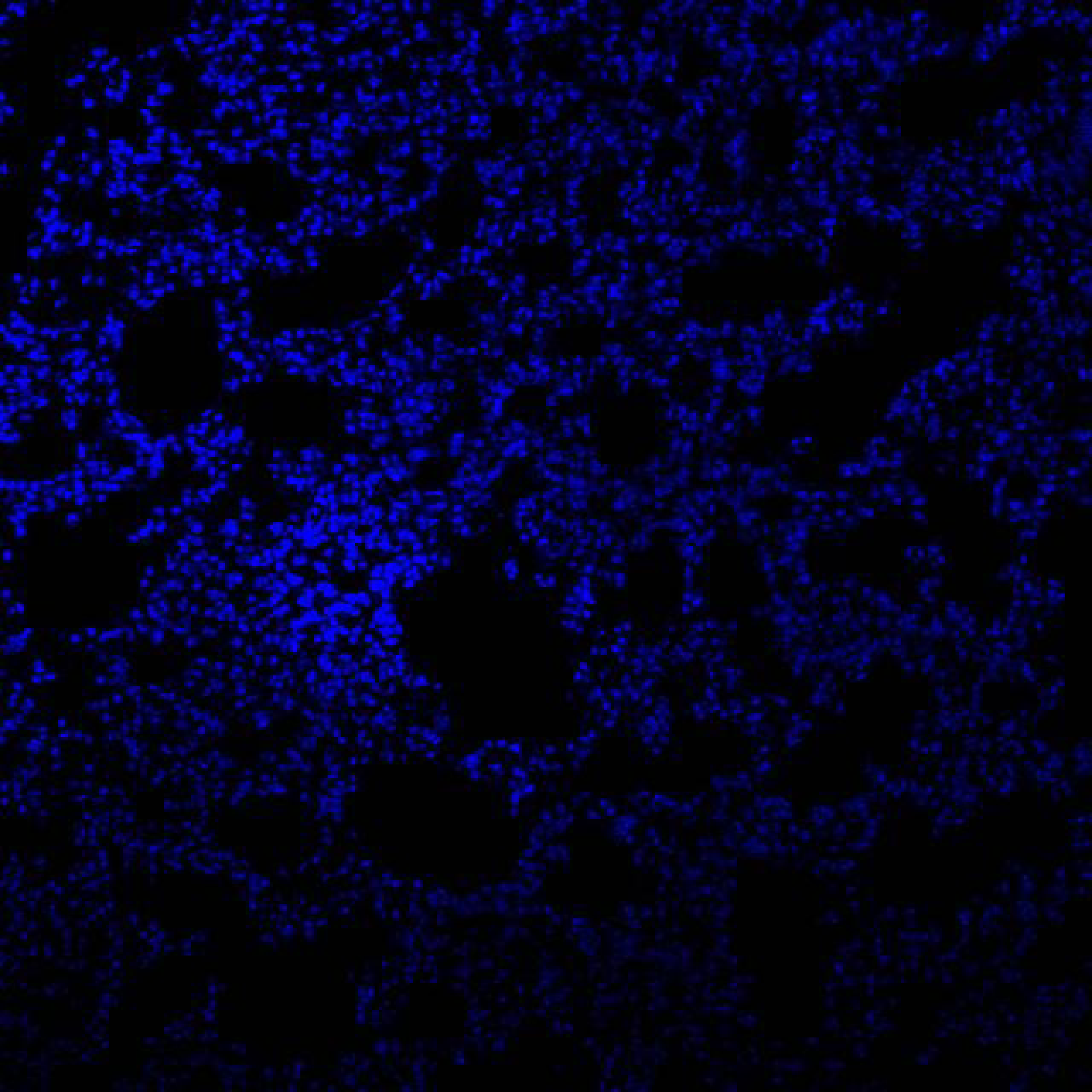

Supplement: S1 Raw Images — (ZIP) [file pone.0322653.s001.zip › S1_raw_images1-tunel and HE picture/TUNEL/CA4-002 20X dapi.tif]

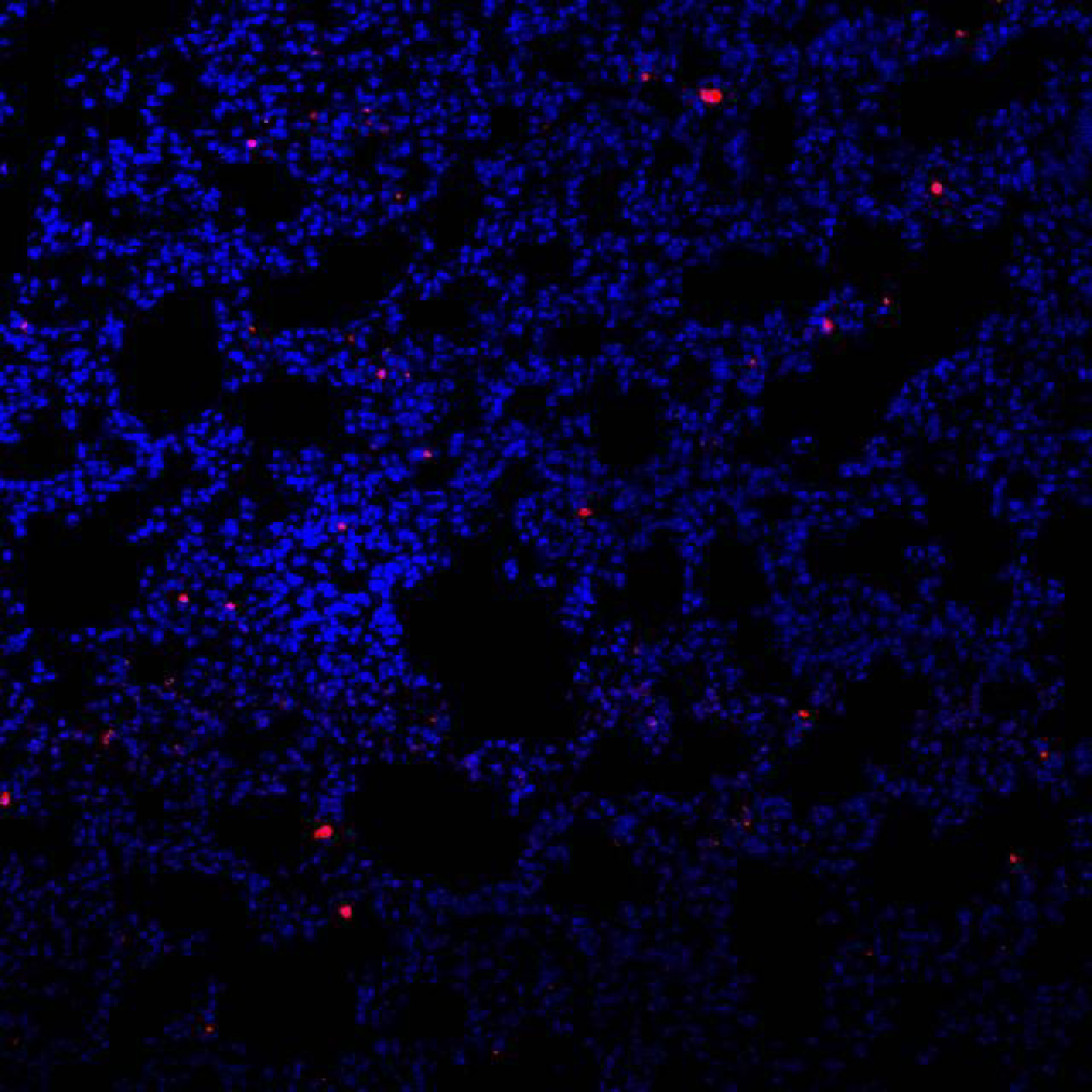

Supplement: S1 Raw Images — (ZIP) [file pone.0322653.s001.zip › S1_raw_images1-tunel and HE picture/TUNEL/CA4-002 20X.tif]

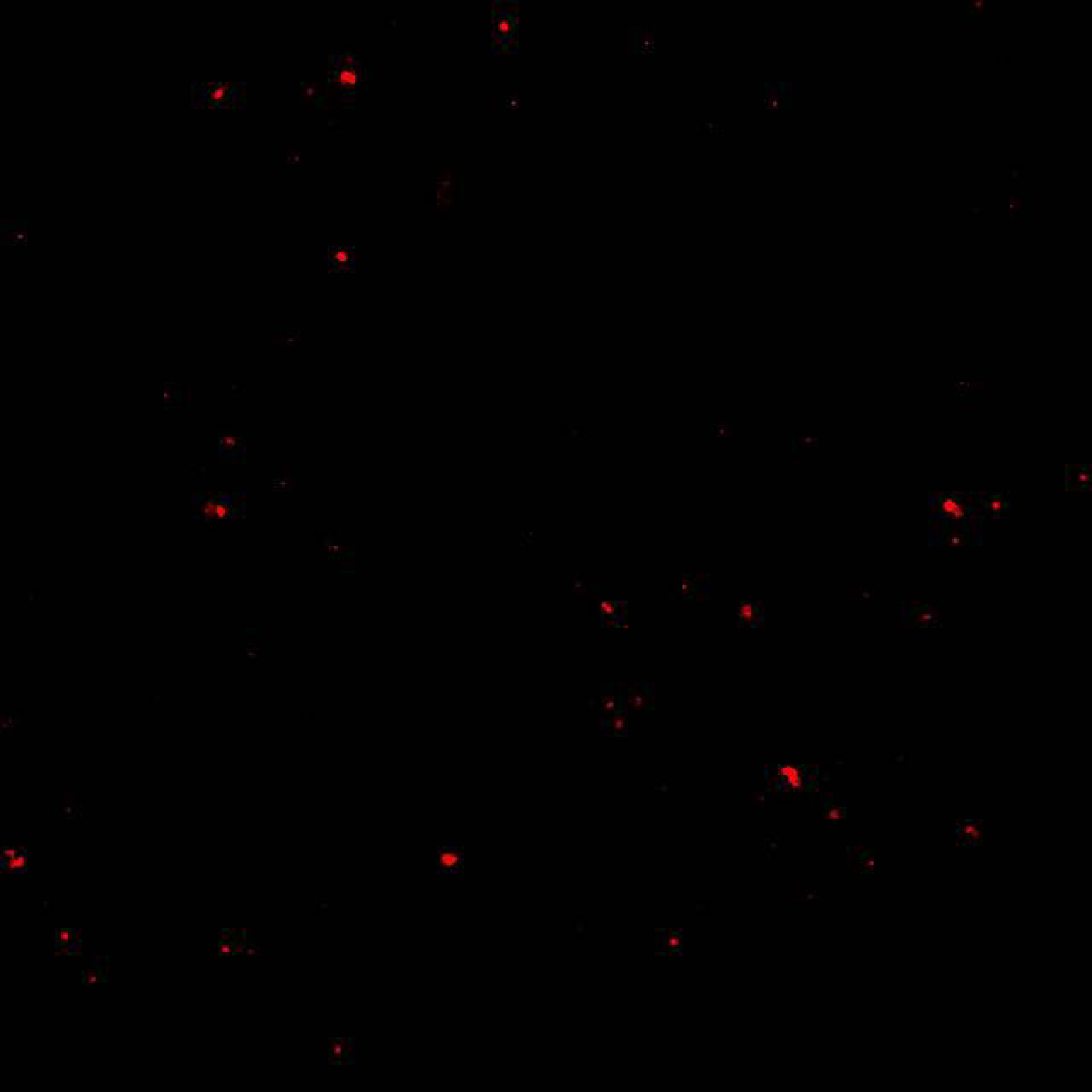

Supplement: S1 Raw Images — (ZIP) [file pone.0322653.s001.zip › S1_raw_images1-tunel and HE picture/TUNEL/CA4-003 20X cy3.tif]

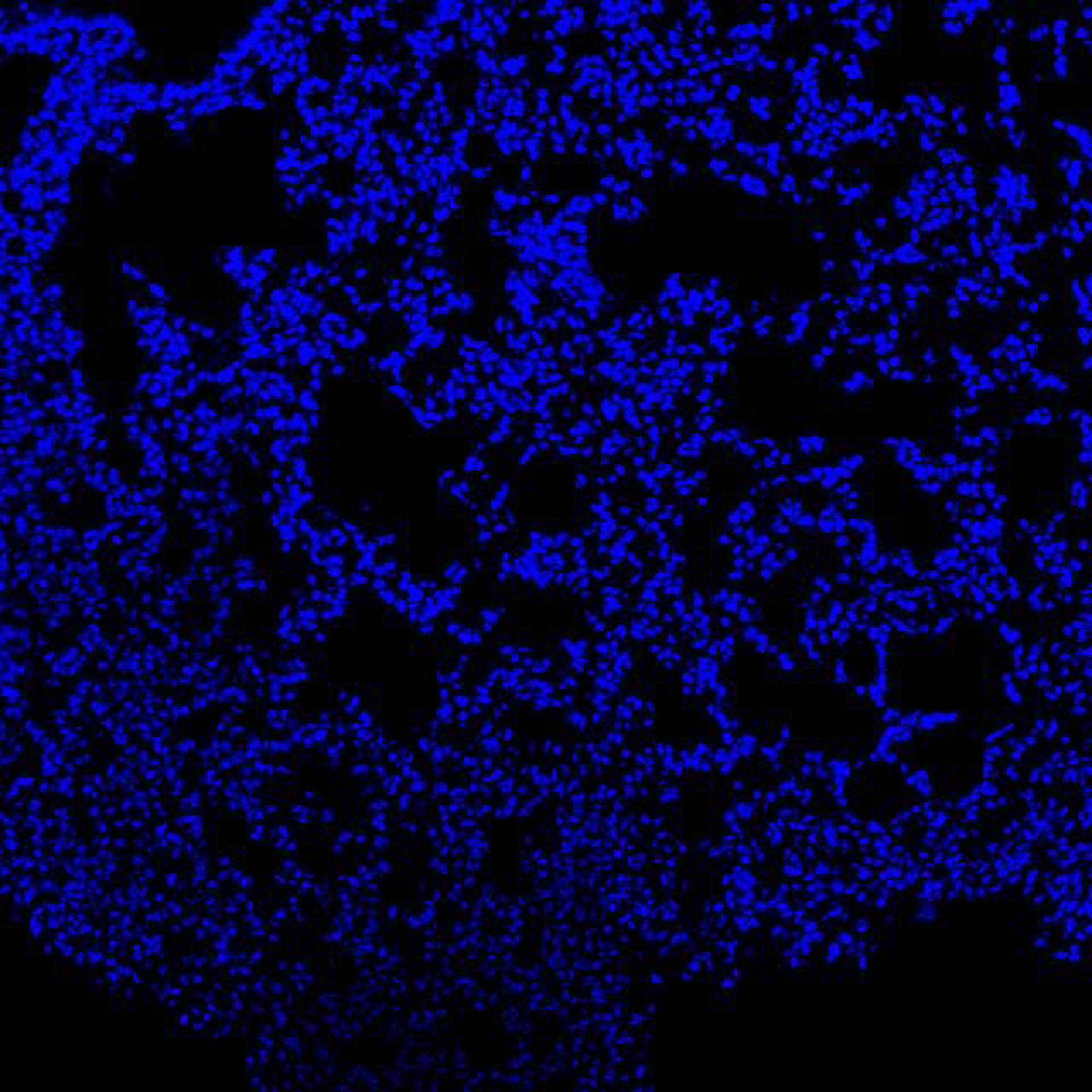

Supplement: S1 Raw Images — (ZIP) [file pone.0322653.s001.zip › S1_raw_images1-tunel and HE picture/TUNEL/CA4-003 20X dapi.tif]

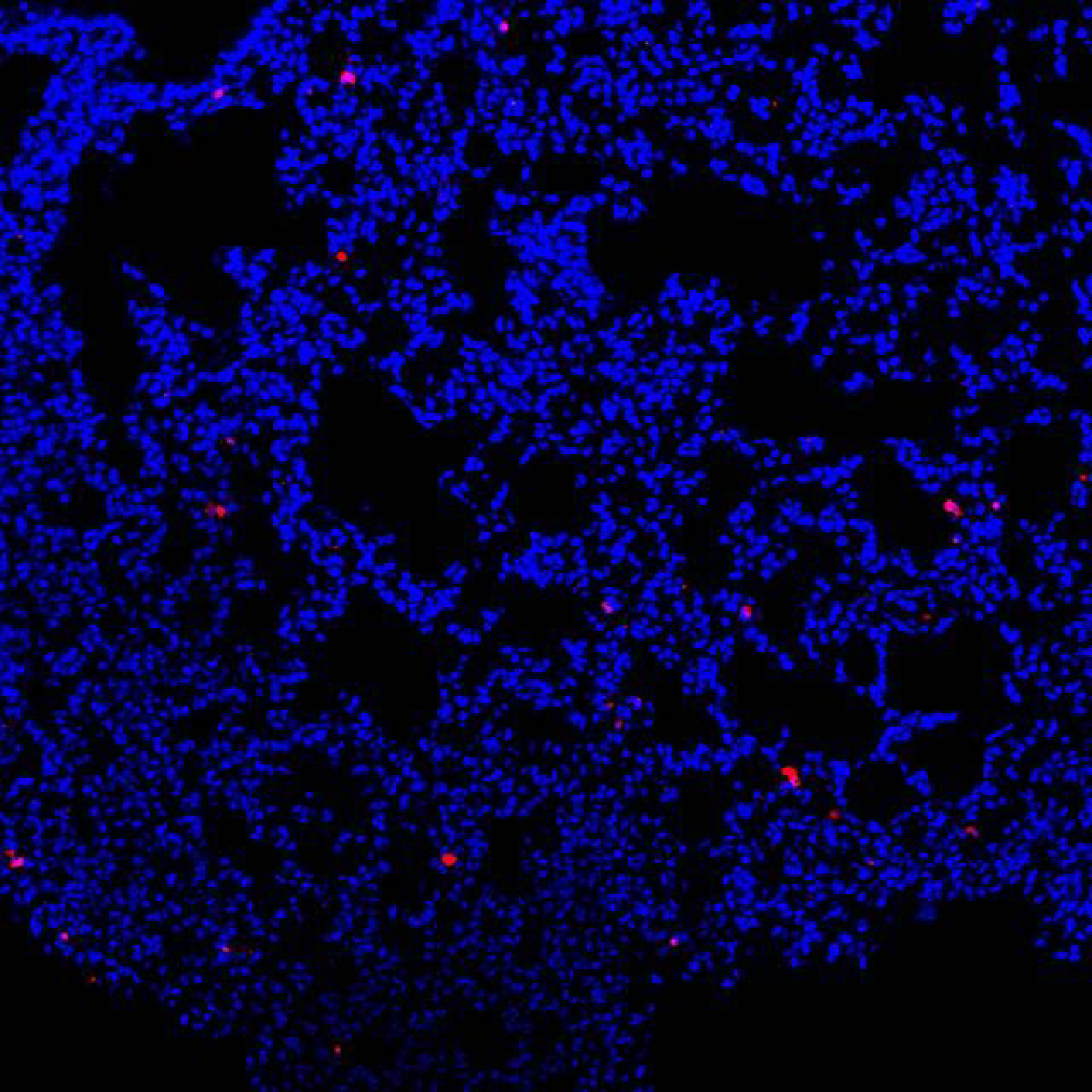

Supplement: S1 Raw Images — (ZIP) [file pone.0322653.s001.zip › S1_raw_images1-tunel and HE picture/TUNEL/CA4-003 20X.tif]

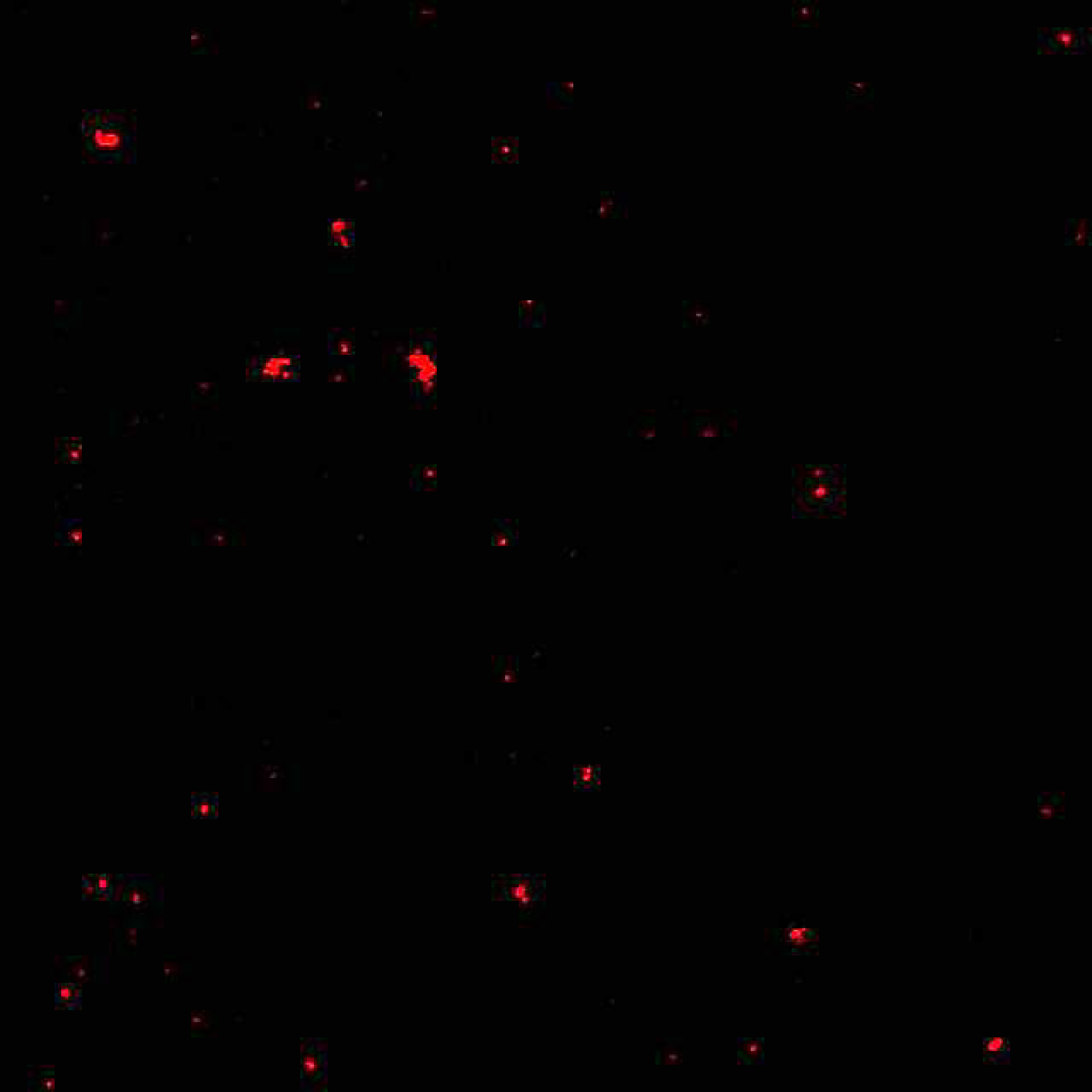

Supplement: S1 Raw Images — (ZIP) [file pone.0322653.s001.zip › S1_raw_images1-tunel and HE picture/TUNEL/CA4-004 20X cy3 (2).tif]

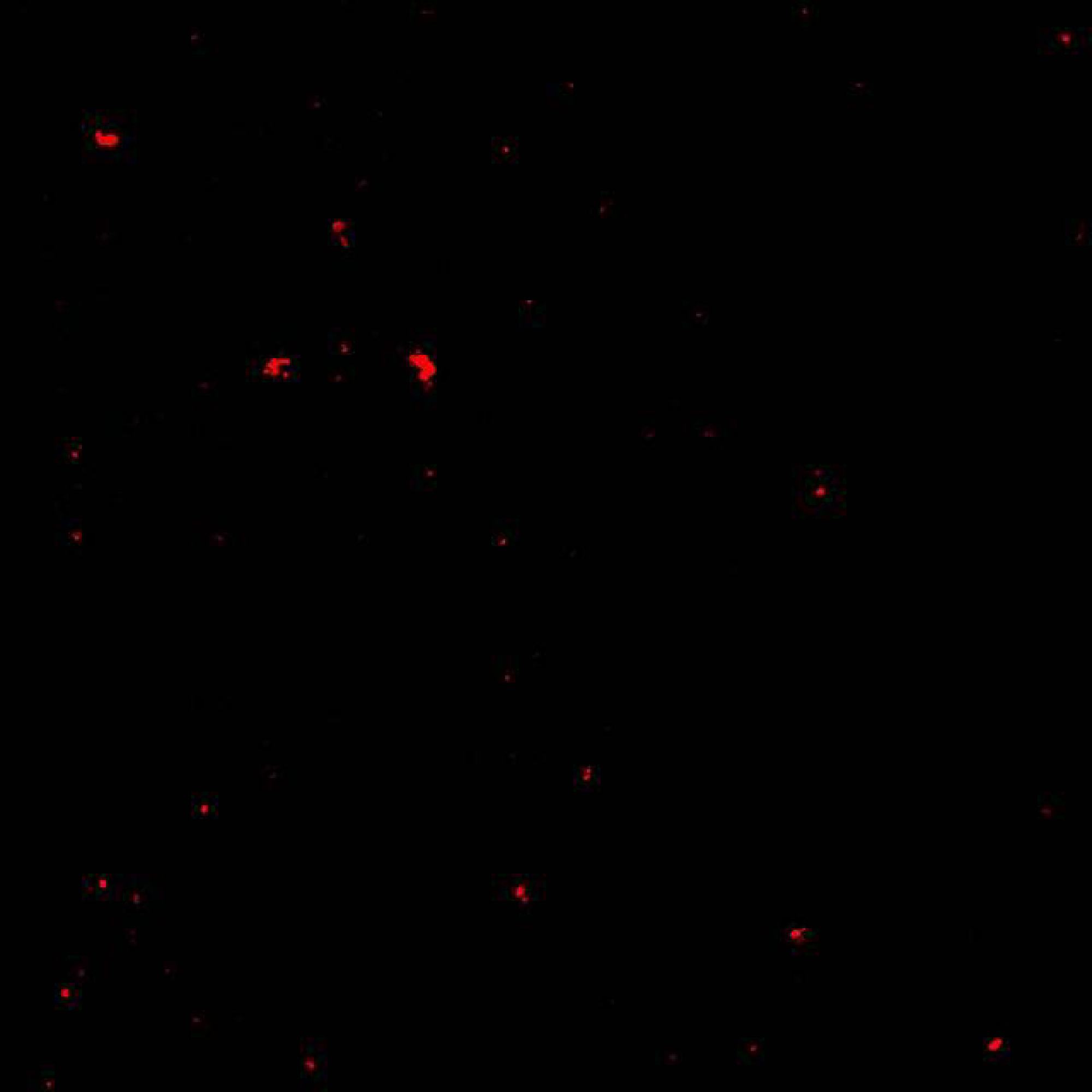

Supplement: S1 Raw Images — (ZIP) [file pone.0322653.s001.zip › S1_raw_images1-tunel and HE picture/TUNEL/CA4-004 20X cy3.tif]

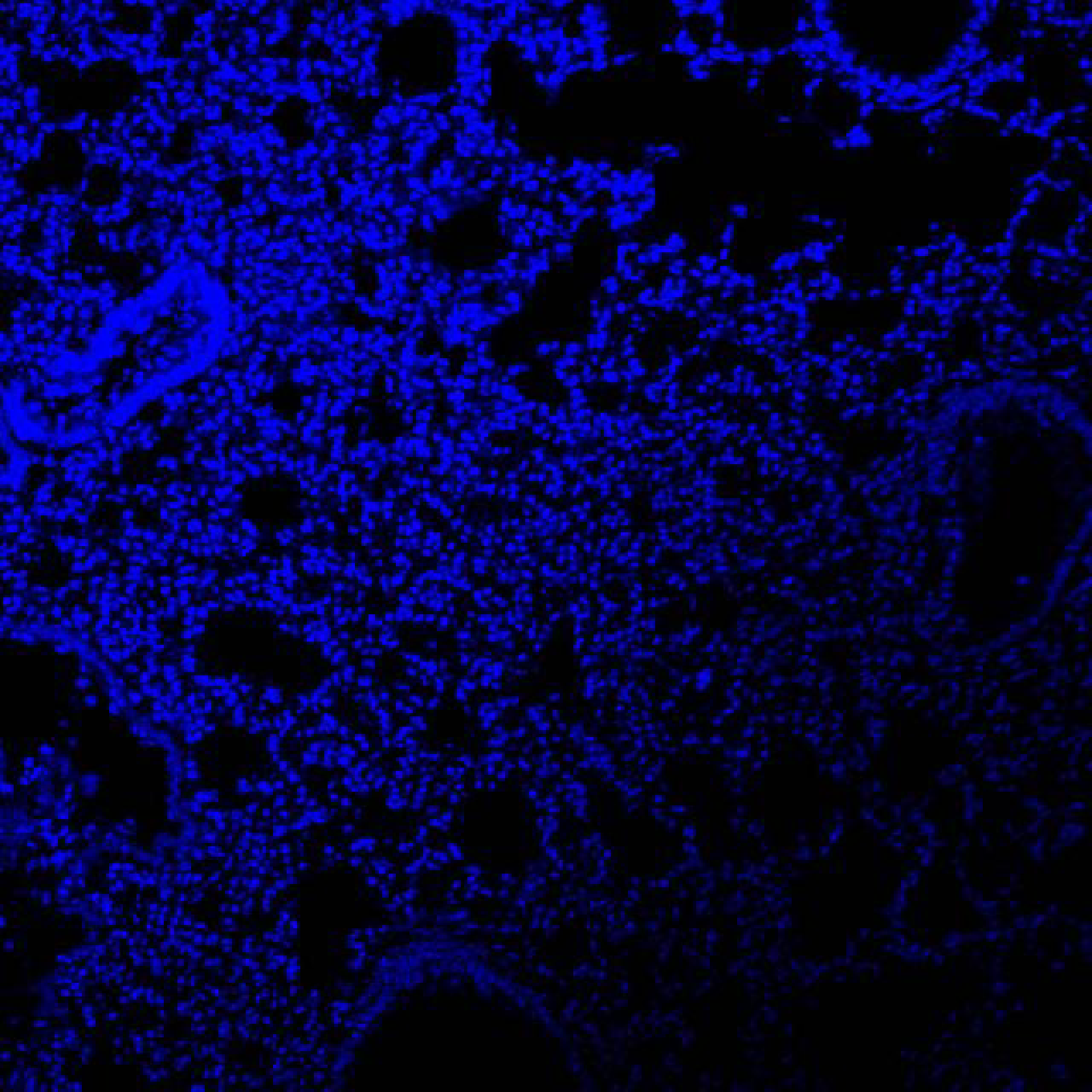

Supplement: S1 Raw Images — (ZIP) [file pone.0322653.s001.zip › S1_raw_images1-tunel and HE picture/TUNEL/CA4-004 20X dapi.tif]

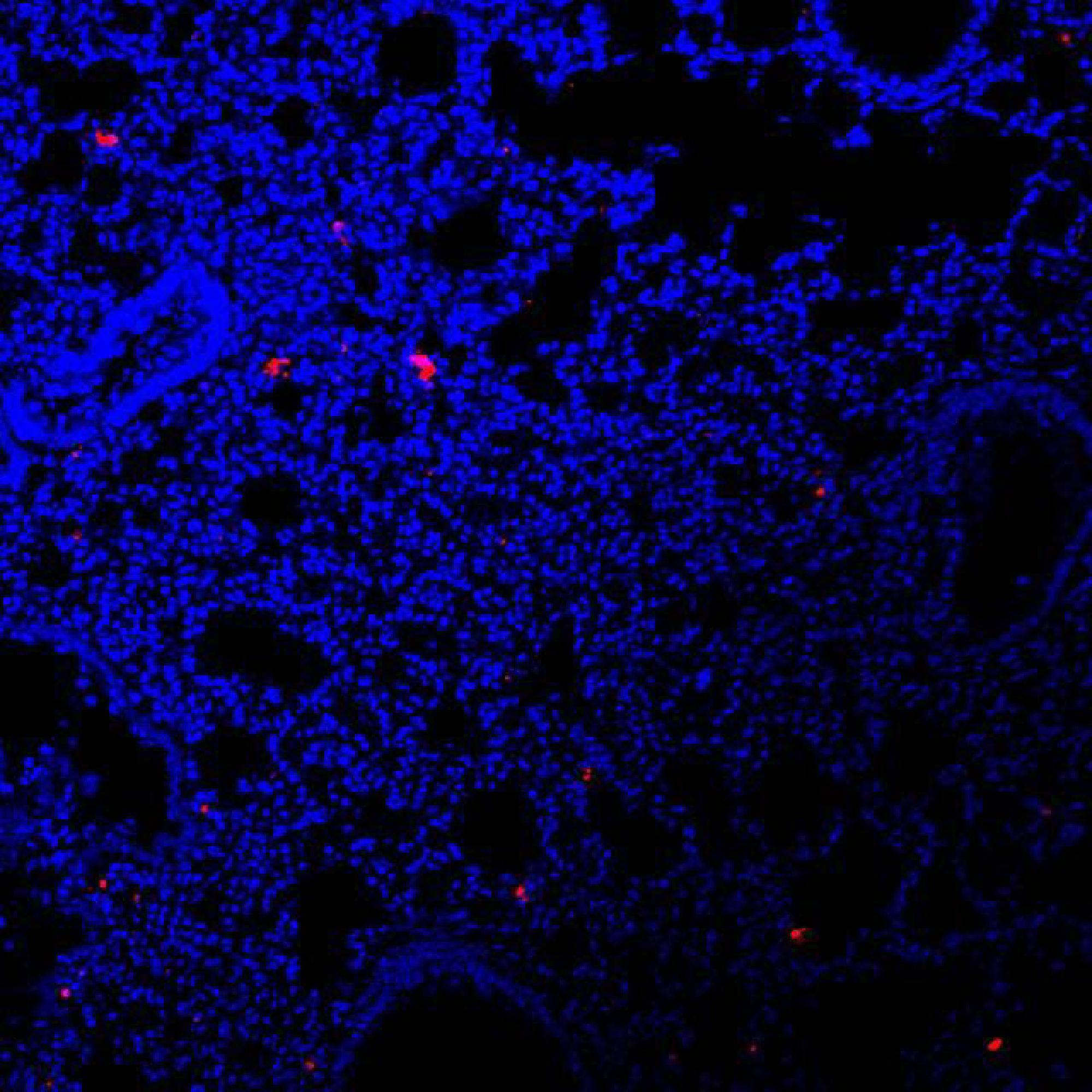

Supplement: S1 Raw Images — (ZIP) [file pone.0322653.s001.zip › S1_raw_images1-tunel and HE picture/TUNEL/CA4-004 20X.tif]

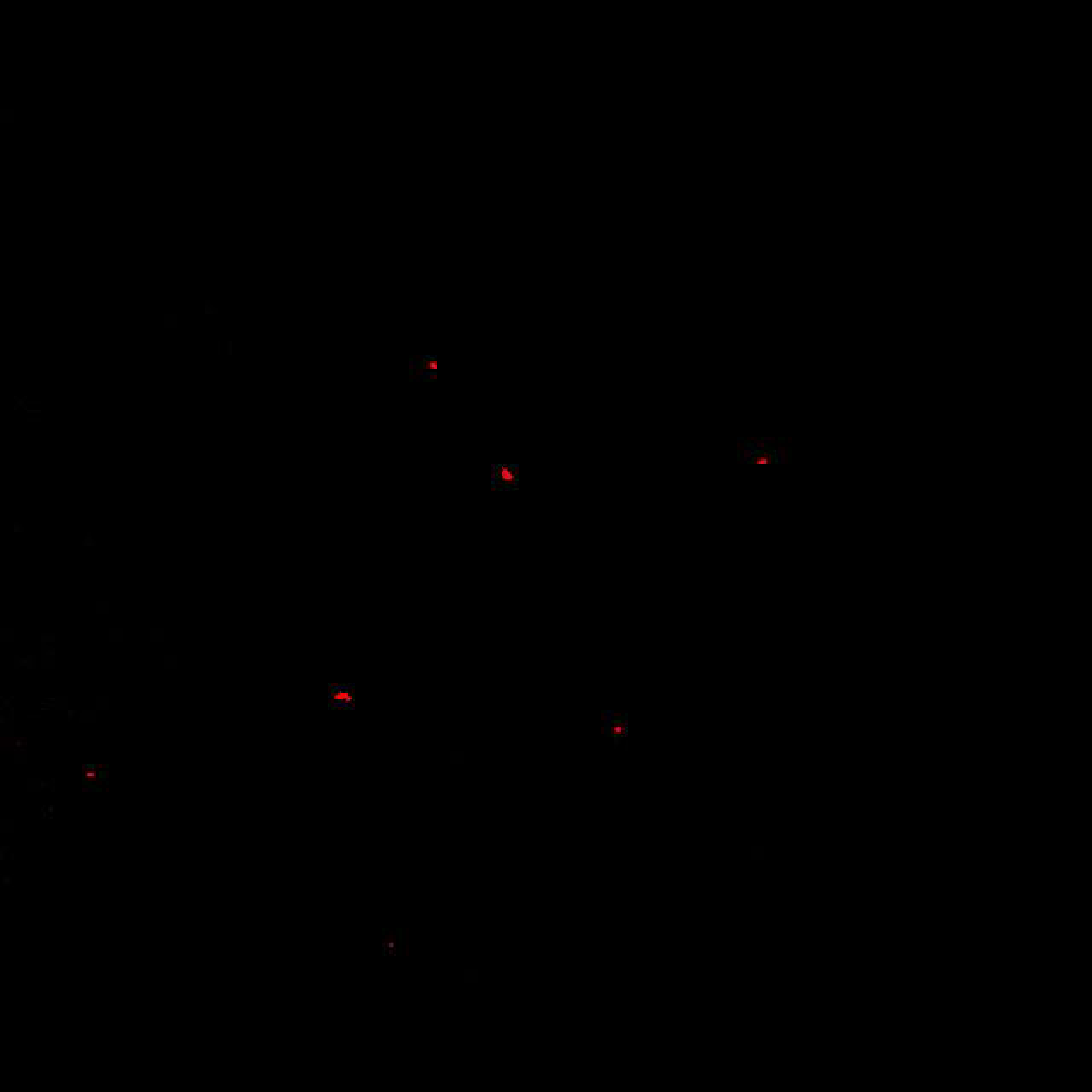

Supplement: S1 Raw Images — (ZIP) [file pone.0322653.s001.zip › S1_raw_images1-tunel and HE picture/TUNEL/LD+A5-001 20X cy3.tif]

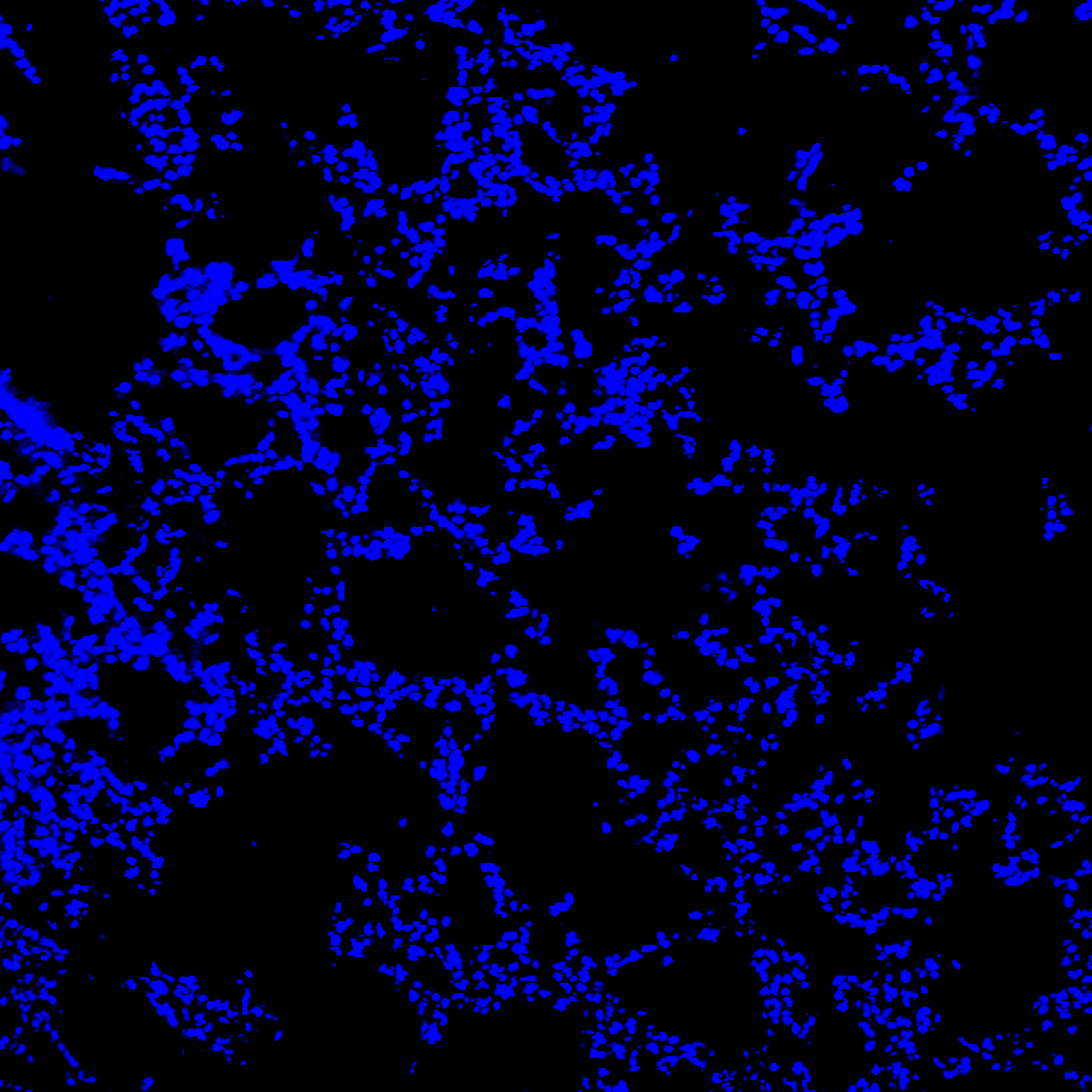

Supplement: S1 Raw Images — (ZIP) [file pone.0322653.s001.zip › S1_raw_images1-tunel and HE picture/TUNEL/LD+A5-001 20X dapi.tif]

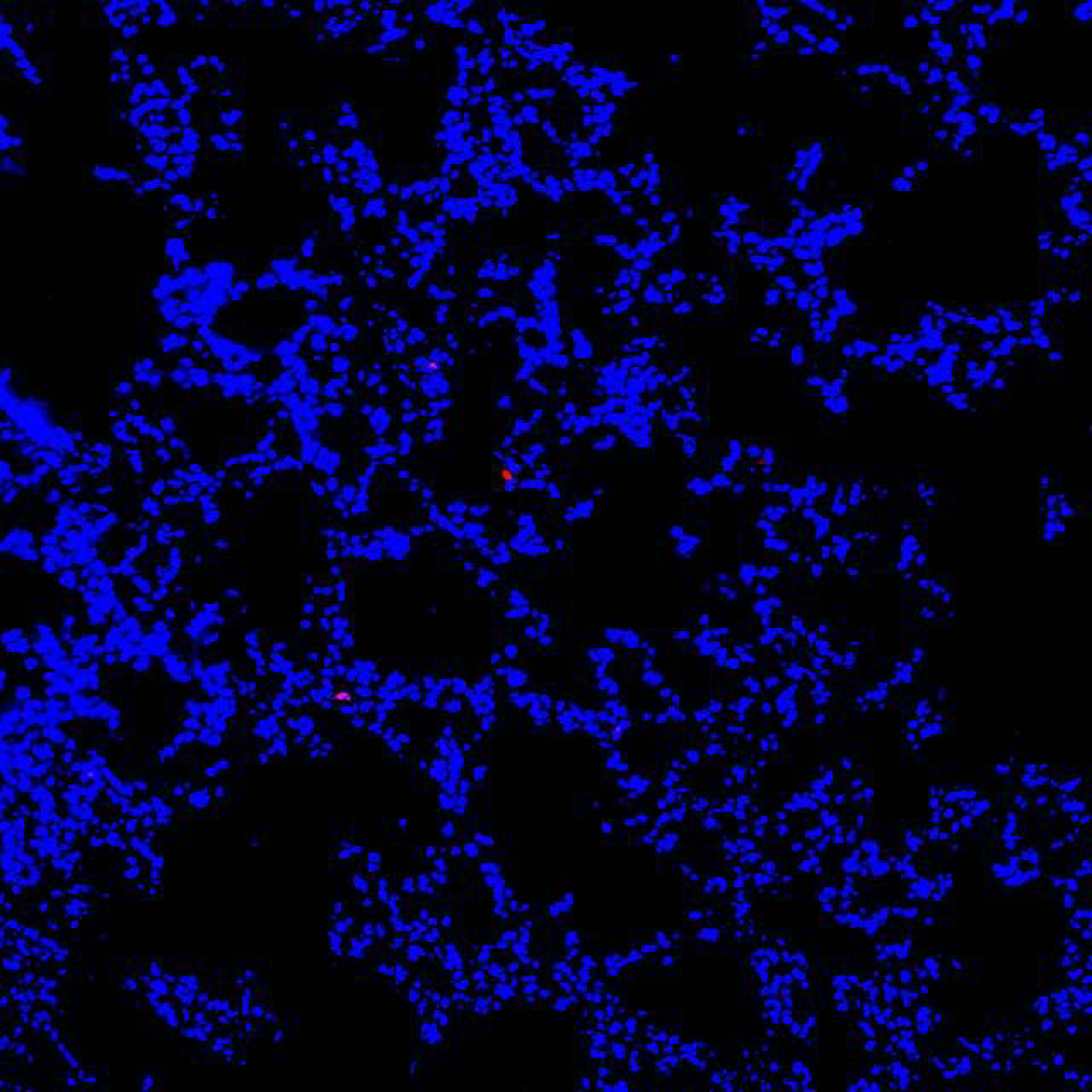

Supplement: S1 Raw Images — (ZIP) [file pone.0322653.s001.zip › S1_raw_images1-tunel and HE picture/TUNEL/LD+A5-001 20X.tif]

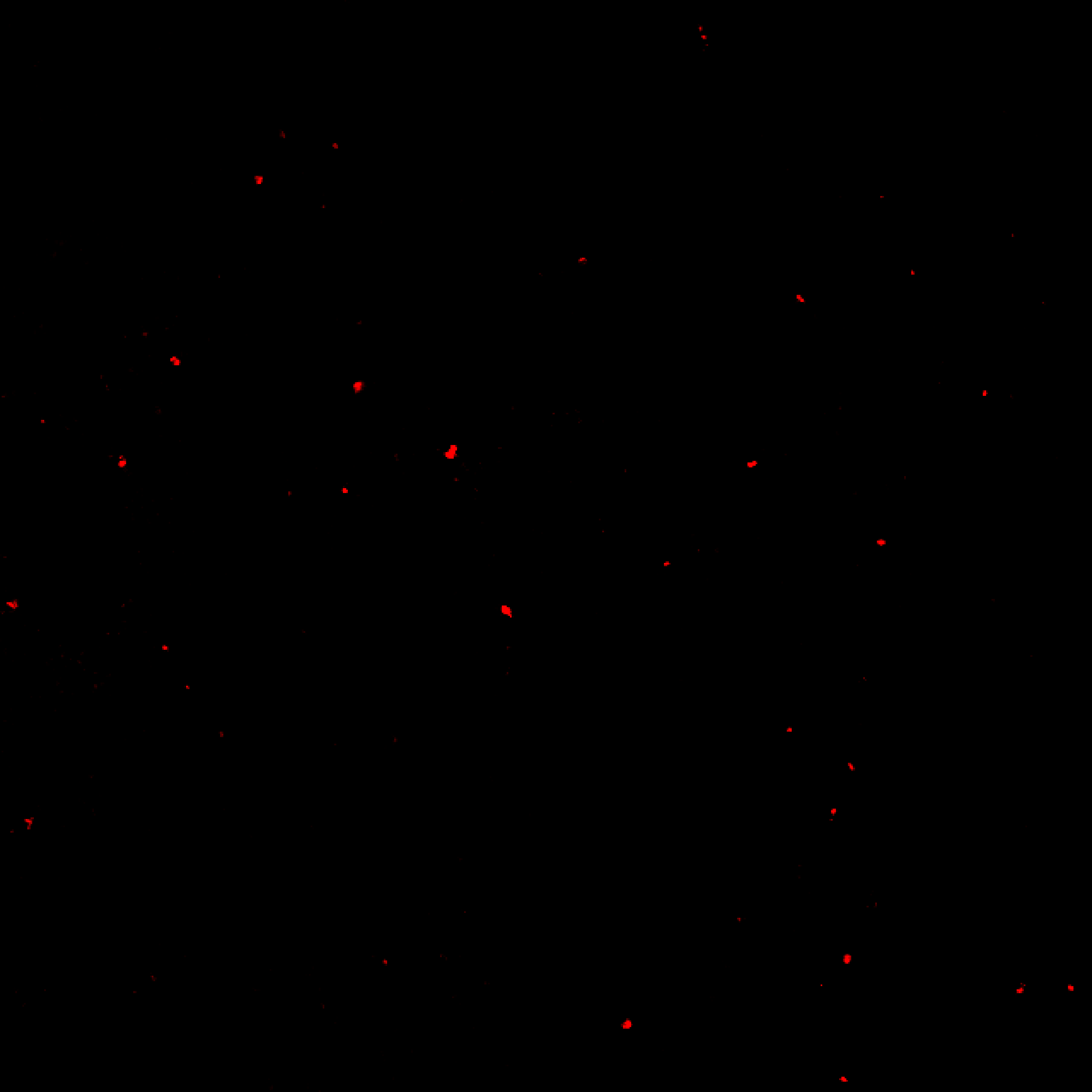

Supplement: S1 Raw Images — (ZIP) [file pone.0322653.s001.zip › S1_raw_images1-tunel and HE picture/TUNEL/LD+A5-005 20X cy3.tif]

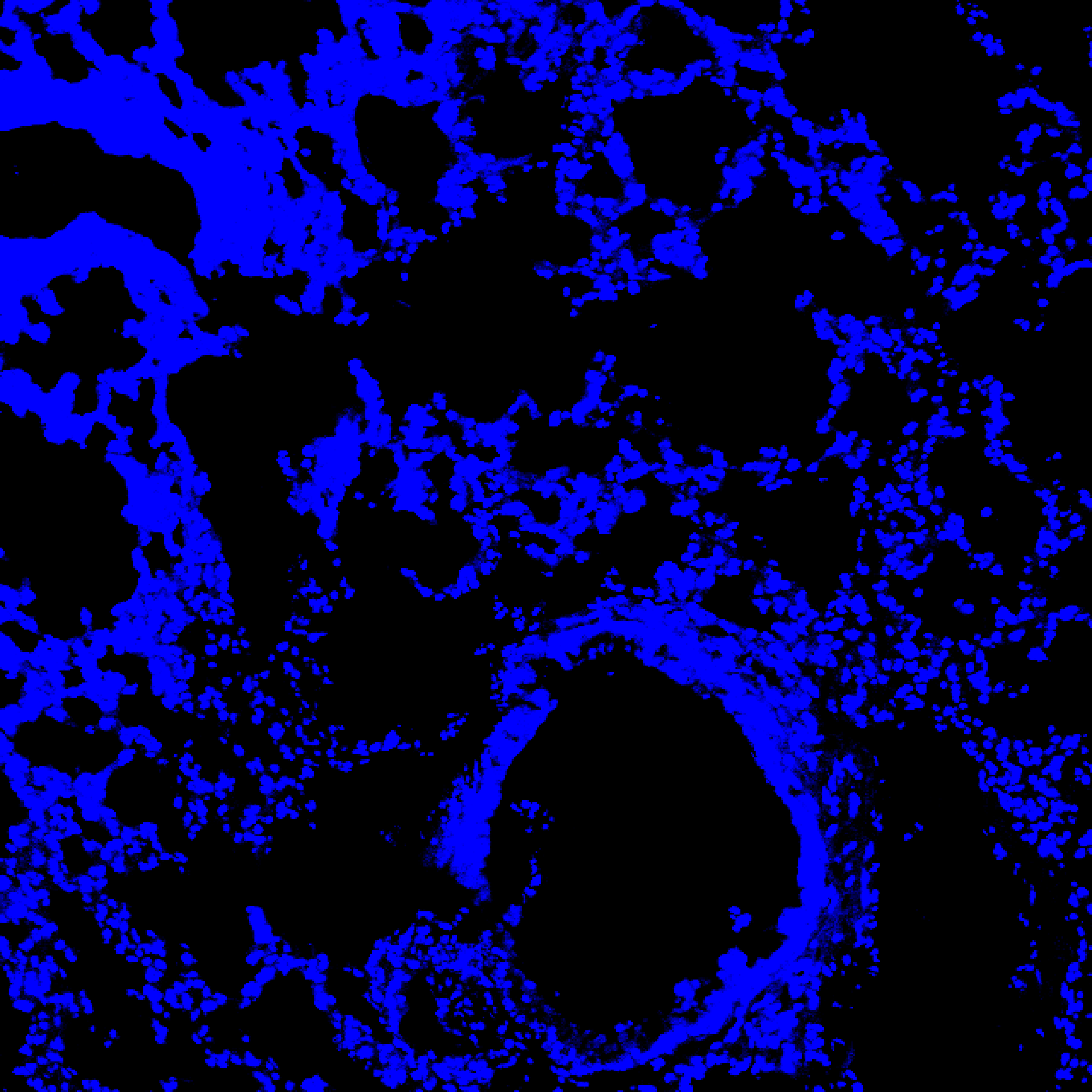

Supplement: S1 Raw Images — (ZIP) [file pone.0322653.s001.zip › S1_raw_images1-tunel and HE picture/TUNEL/LD+A5-005 20X dapi.tif]

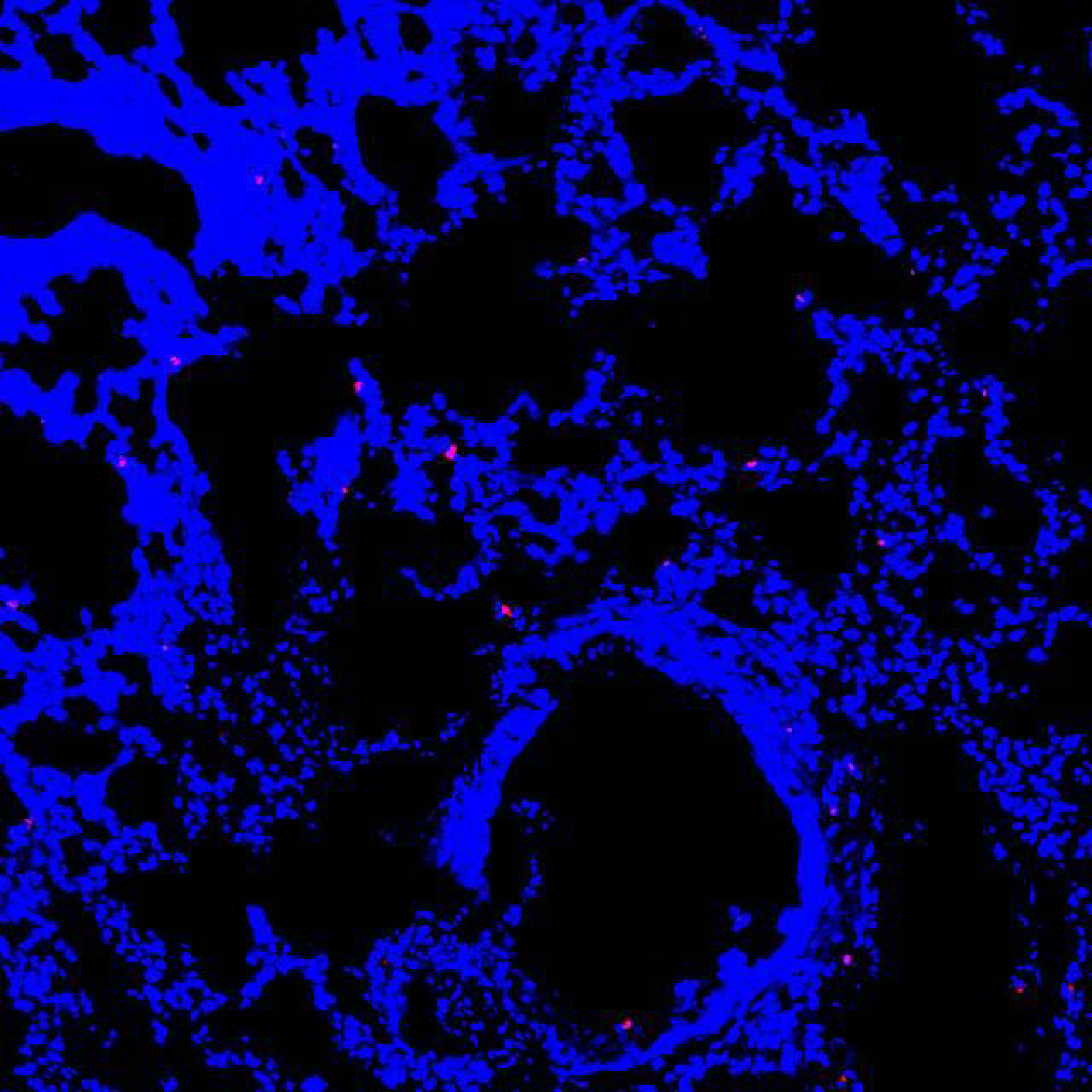

Supplement: S1 Raw Images — (ZIP) [file pone.0322653.s001.zip › S1_raw_images1-tunel and HE picture/TUNEL/LD+A5-005 20X.tif]

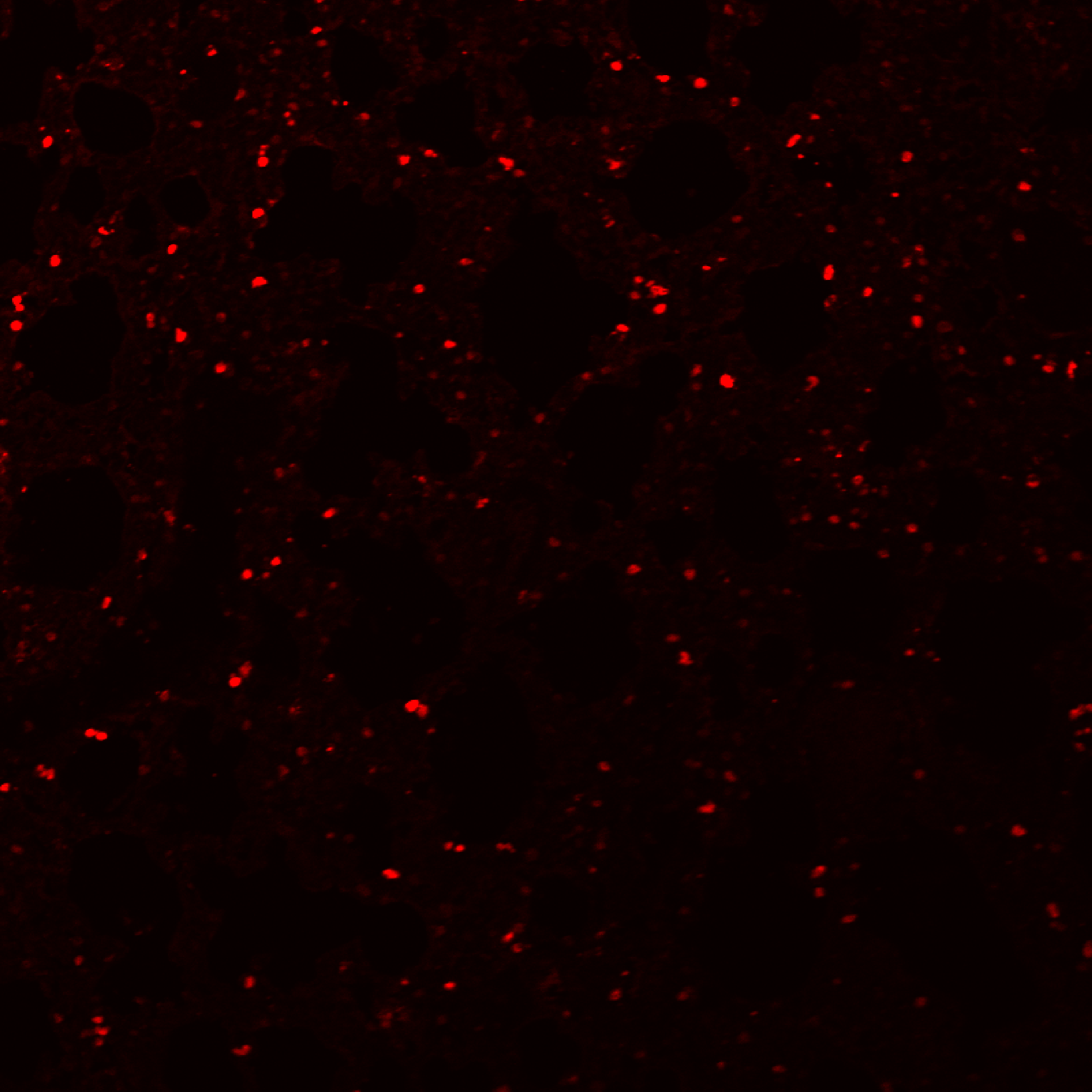

Supplement: S1 Raw Images — (ZIP) [file pone.0322653.s001.zip › S1_raw_images1-tunel and HE picture/TUNEL/picture/3.10-CB2-2-CY3.tif]

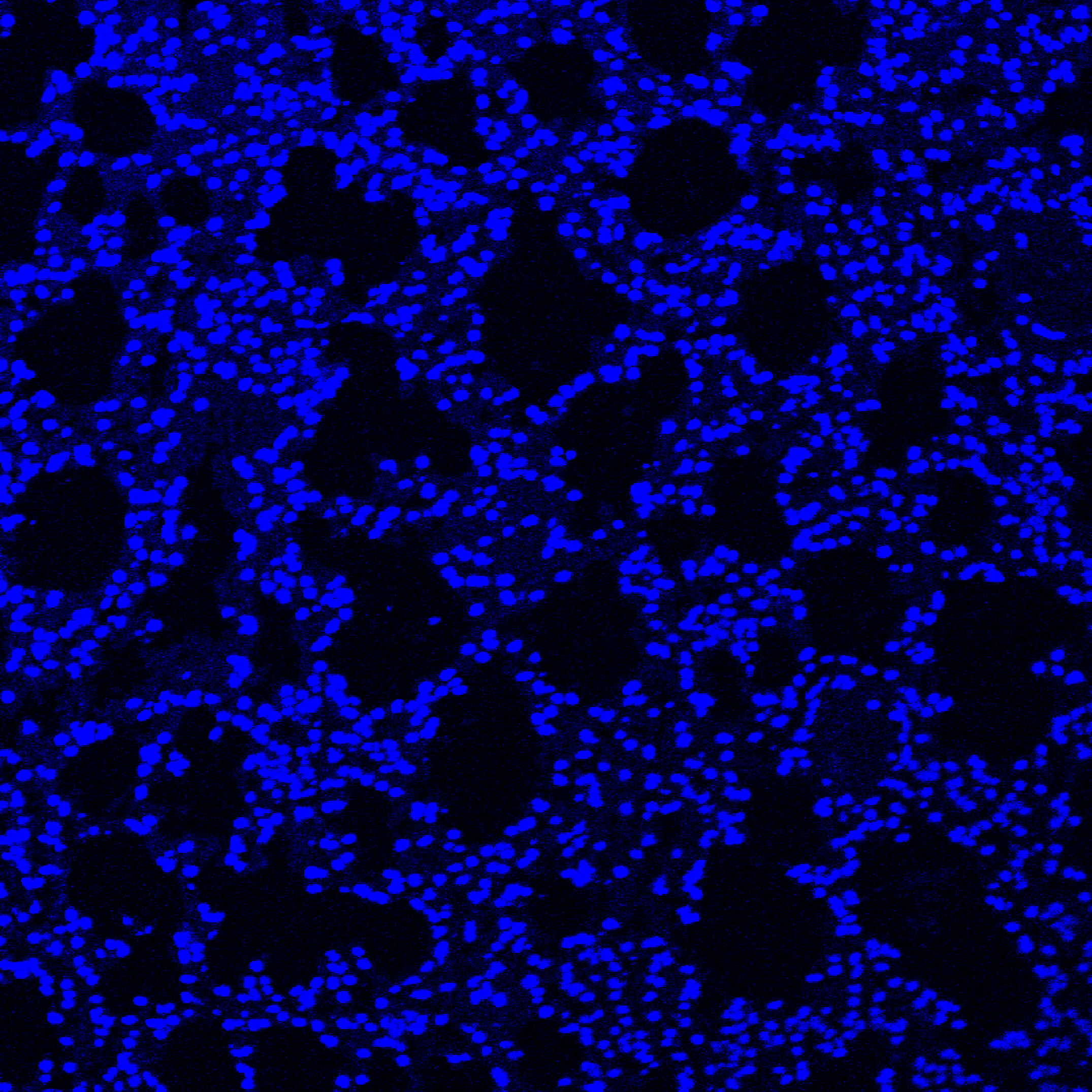

Supplement: S1 Raw Images — (ZIP) [file pone.0322653.s001.zip › S1_raw_images1-tunel and HE picture/TUNEL/picture/3.10-CB2-2-DAPI.tif]

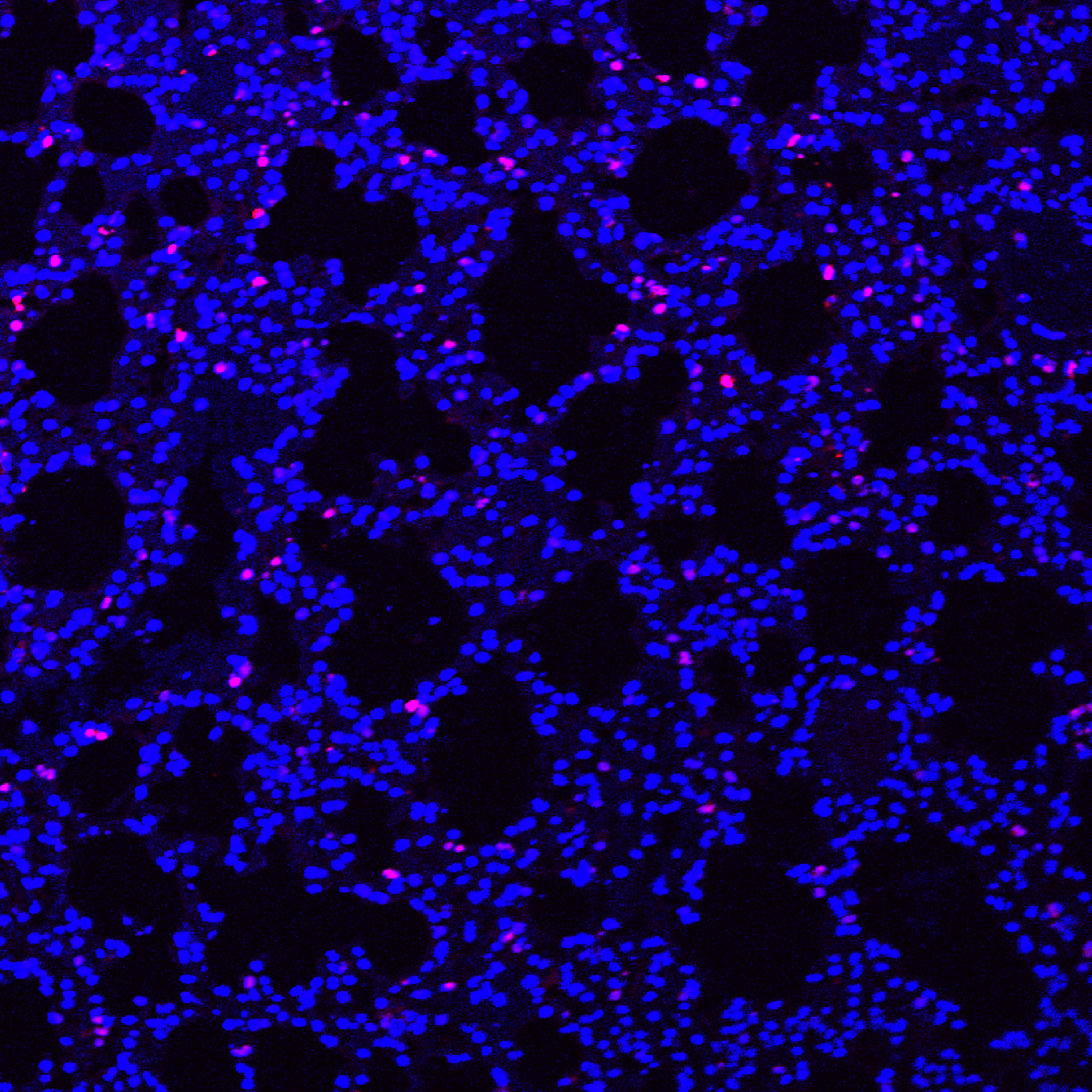

Supplement: S1 Raw Images — (ZIP) [file pone.0322653.s001.zip › S1_raw_images1-tunel and HE picture/TUNEL/picture/3.10-CB2-2.tif]

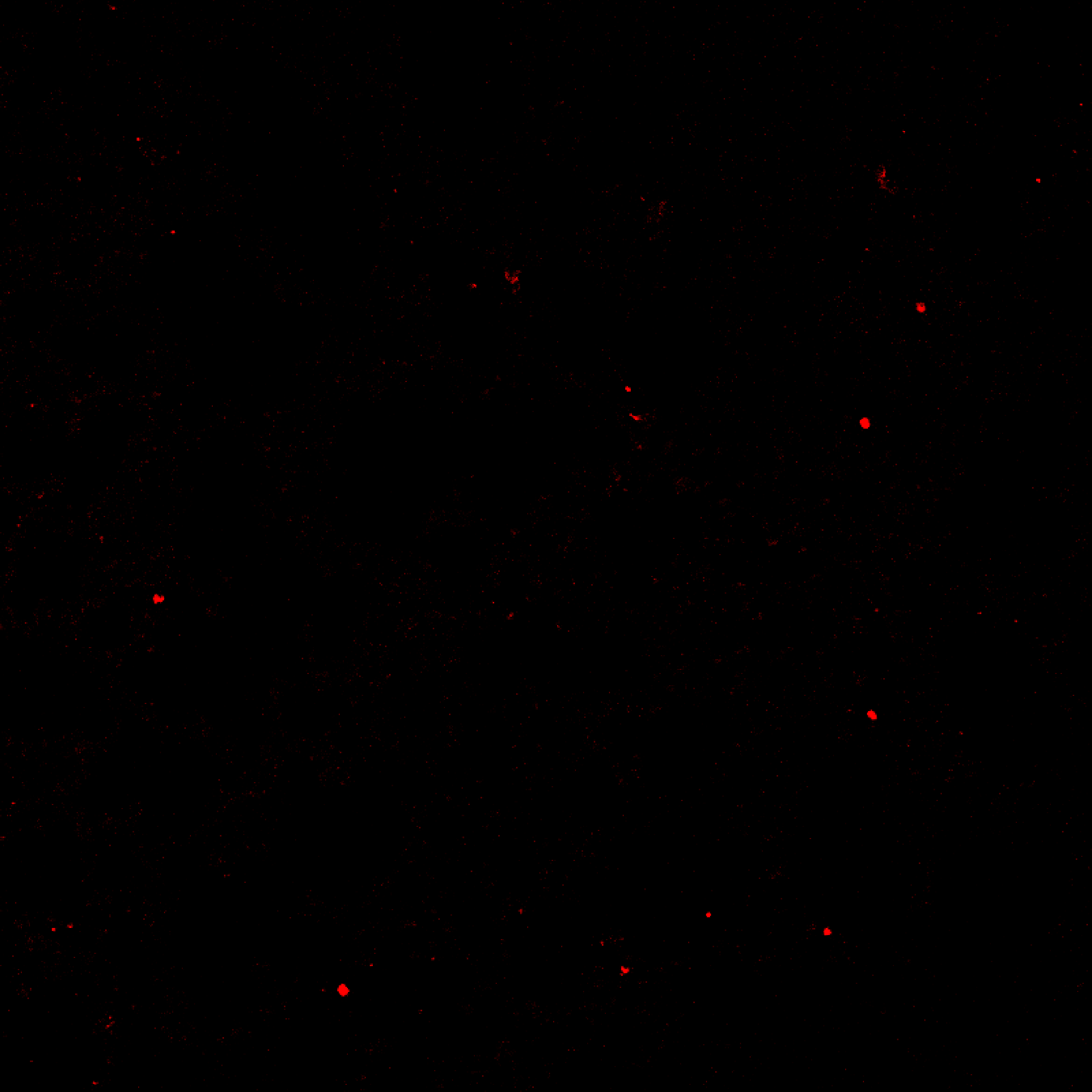

Supplement: S1 Raw Images — (ZIP) [file pone.0322653.s001.zip › S1_raw_images1-tunel and HE picture/TUNEL/picture/3.12-LD1-2-CY3.tif]

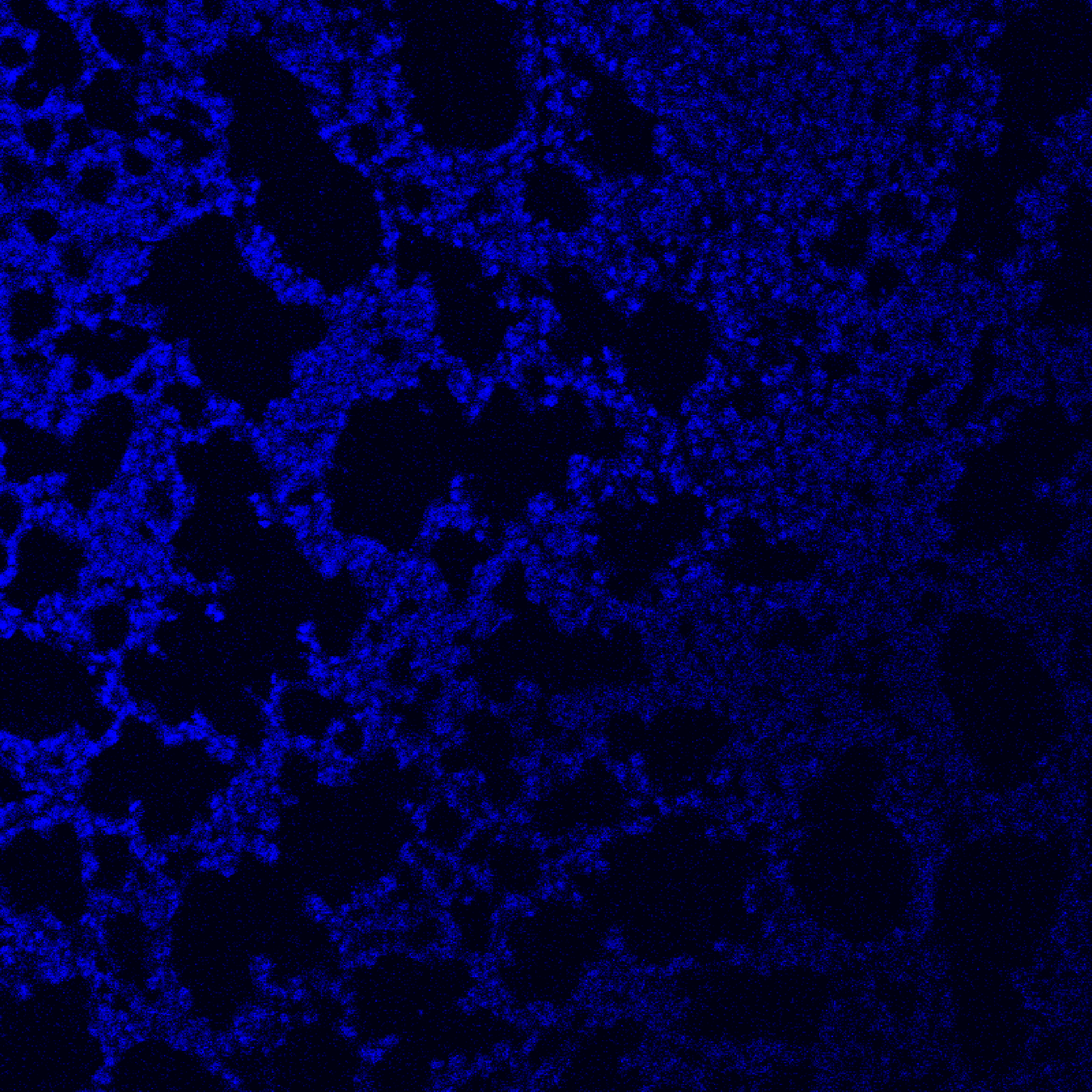

Supplement: S1 Raw Images — (ZIP) [file pone.0322653.s001.zip › S1_raw_images1-tunel and HE picture/TUNEL/picture/3.12-LD1-2-DAPI.tif]

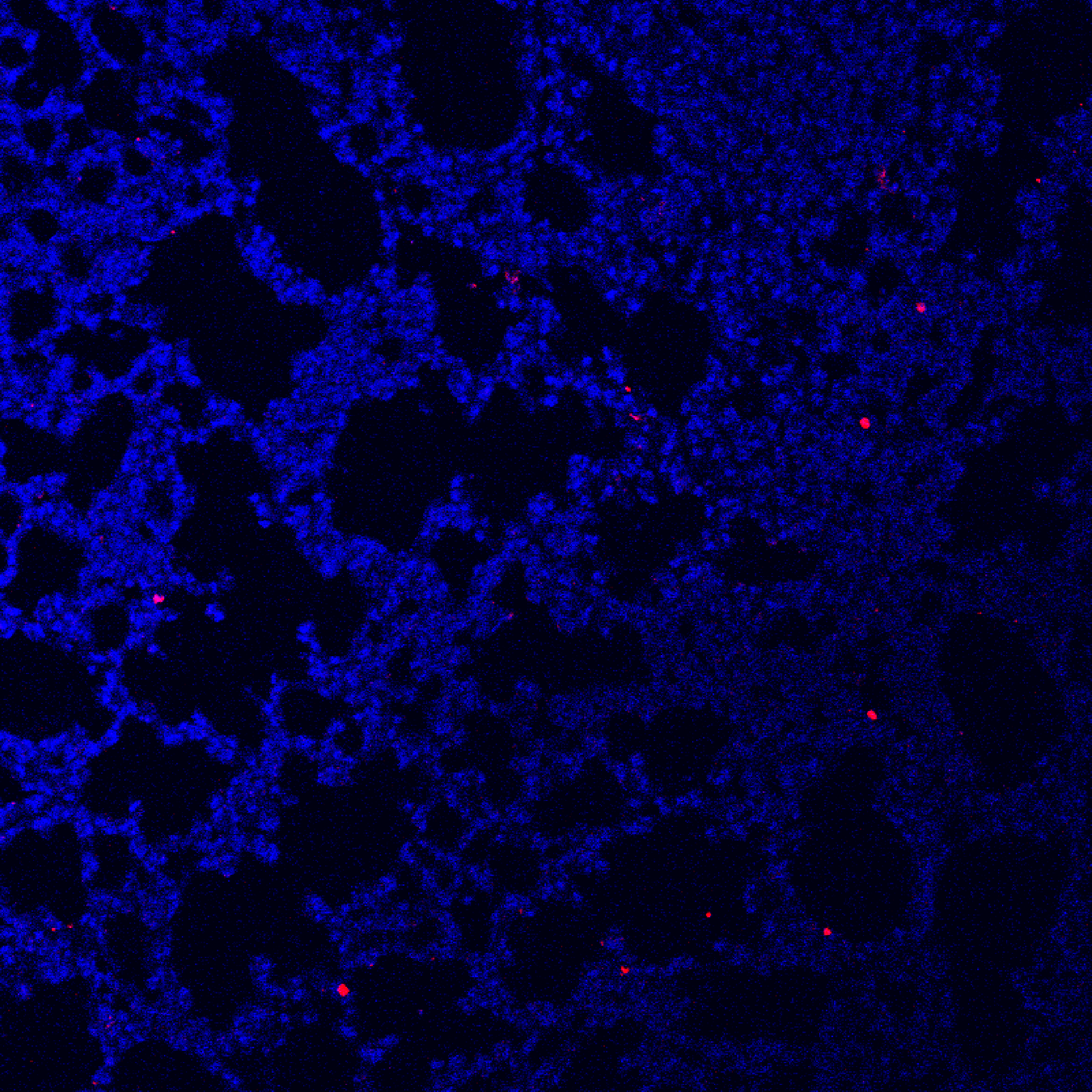

Supplement: S1 Raw Images — (ZIP) [file pone.0322653.s001.zip › S1_raw_images1-tunel and HE picture/TUNEL/picture/3.12-LD1-2.tif]

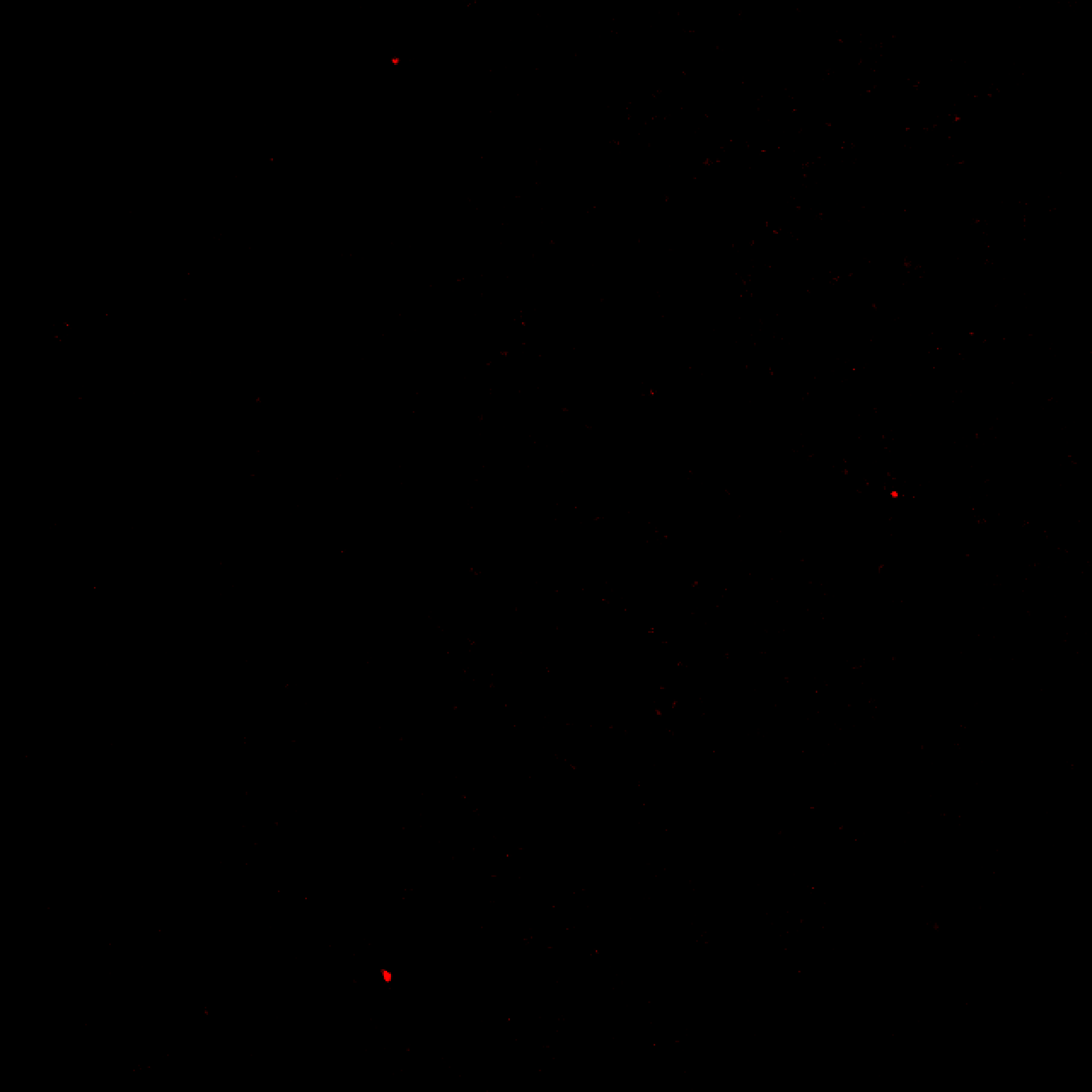

Supplement: S1 Raw Images — (ZIP) [file pone.0322653.s001.zip › S1_raw_images1-tunel and HE picture/TUNEL/picture/N3-002 20X cy3.tif]

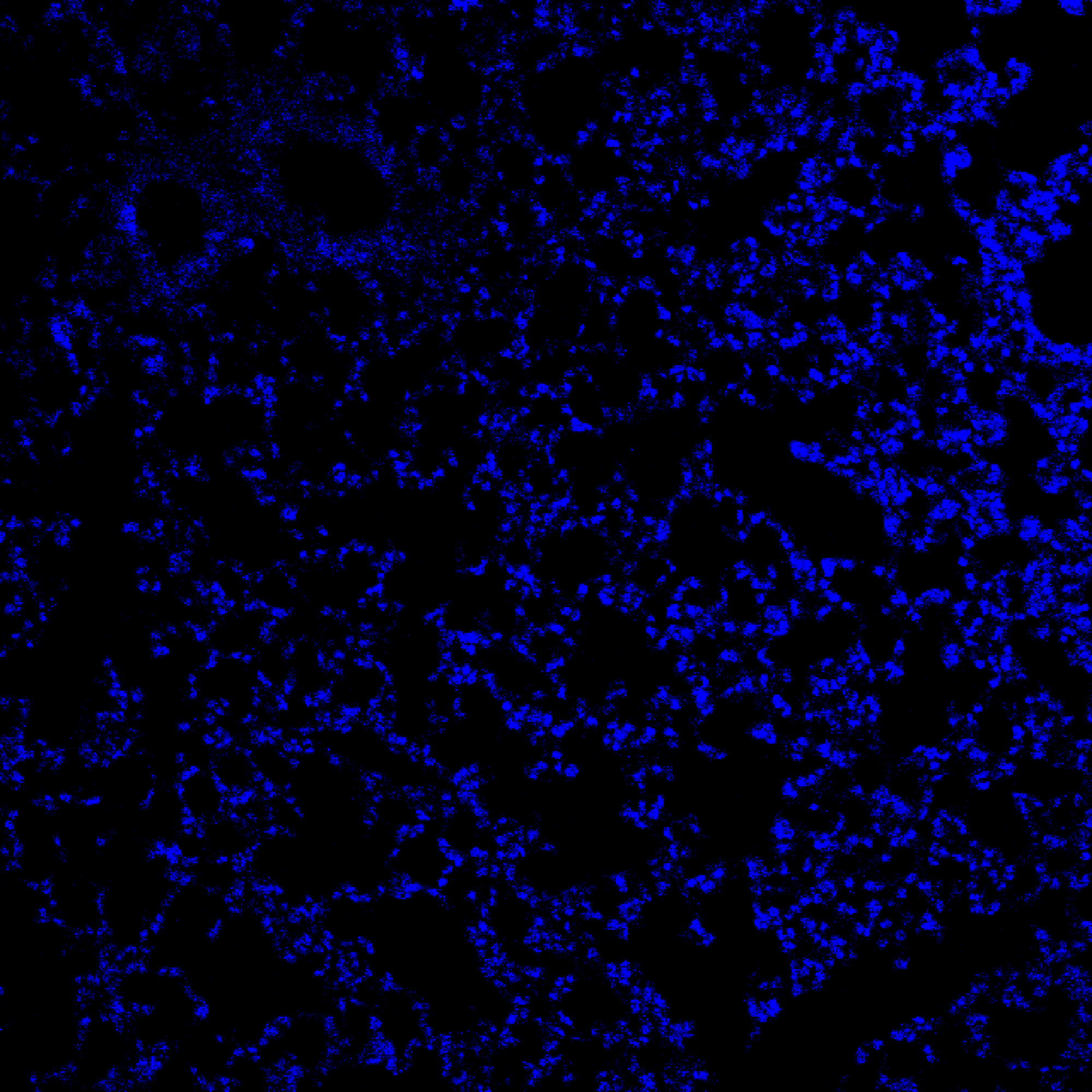

Supplement: S1 Raw Images — (ZIP) [file pone.0322653.s001.zip › S1_raw_images1-tunel and HE picture/TUNEL/picture/N3-002 20X dapi.tif]

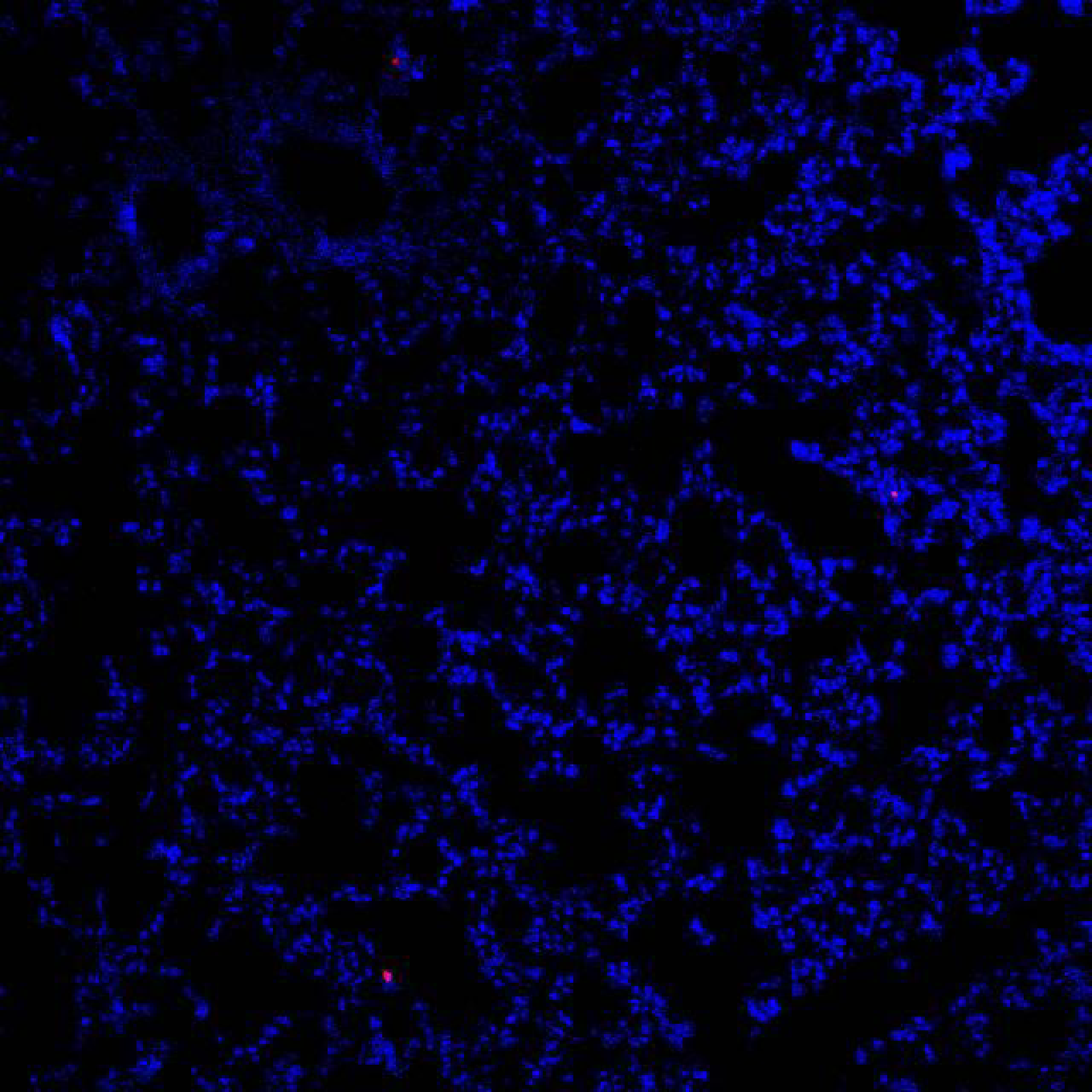

Supplement: S1 Raw Images — (ZIP) [file pone.0322653.s001.zip › S1_raw_images1-tunel and HE picture/TUNEL/picture/N3-002 20X.tif]

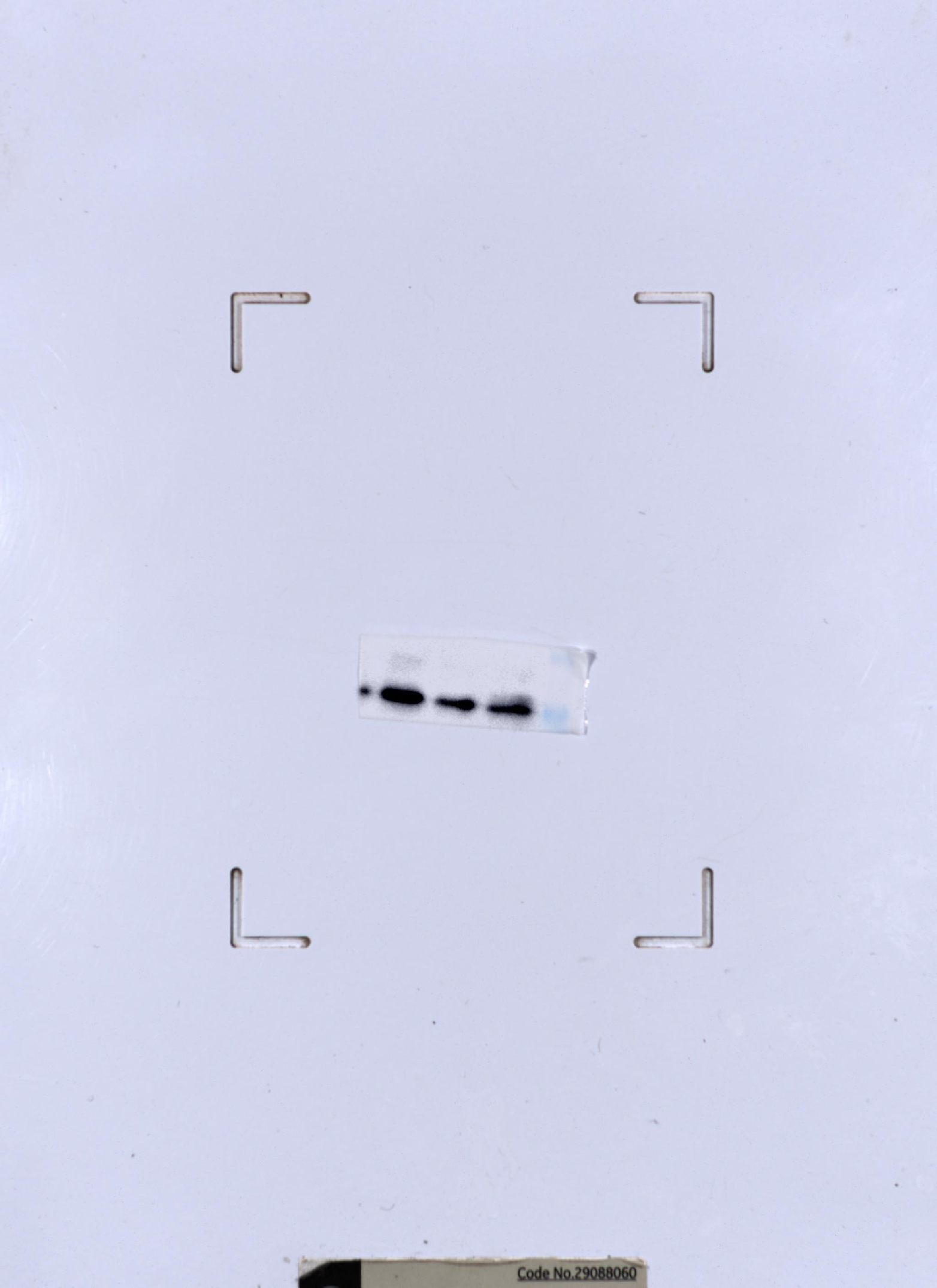

Supplement: S2 Raw Images — (ZIP) [file pone.0322653.s002.zip › S1_raw_images2-wb data/10.17 gp3/10.17-bcl2-gp3-2s.tif]

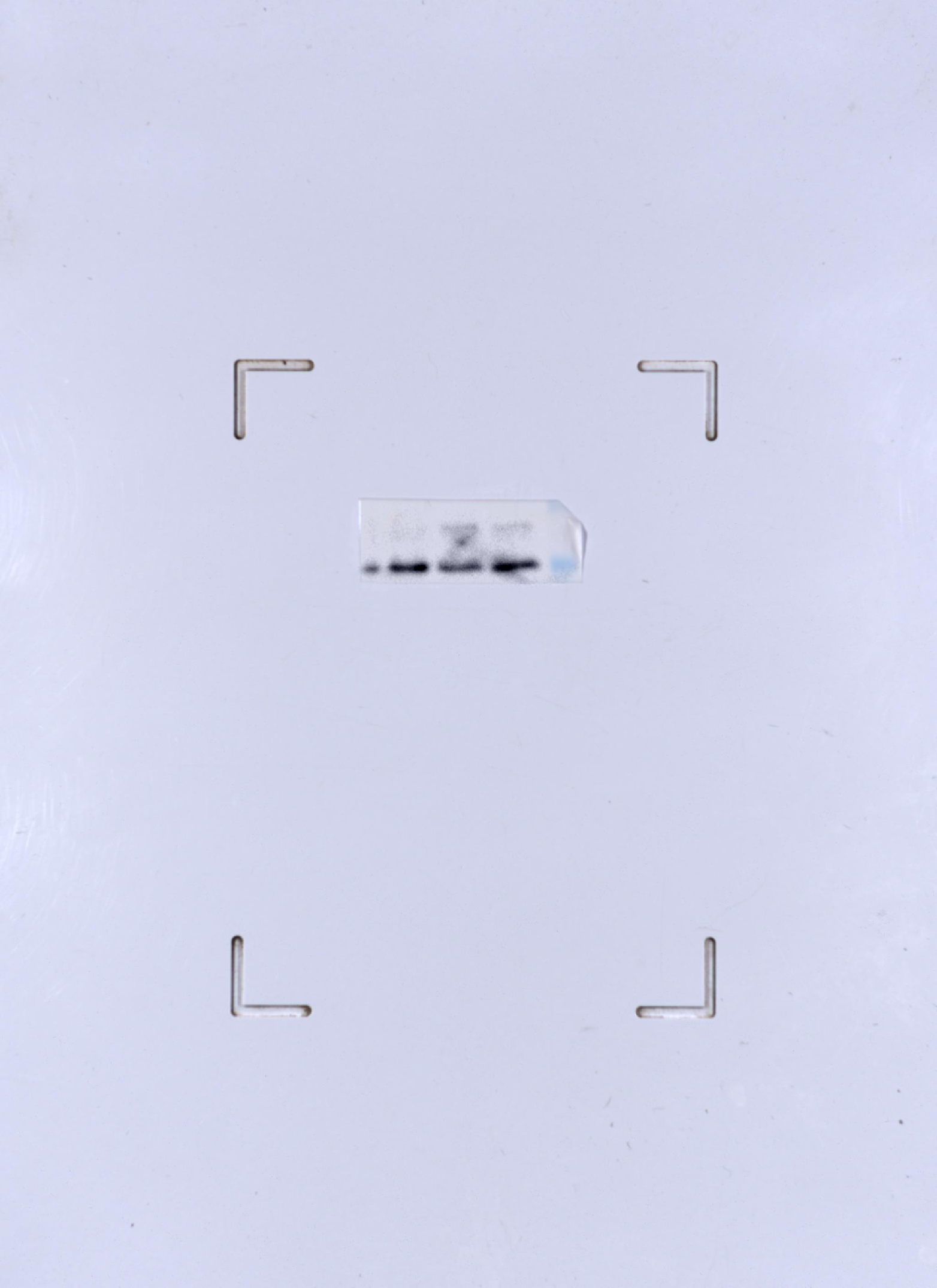

Supplement: S2 Raw Images — (ZIP) [file pone.0322653.s002.zip › S1_raw_images2-wb data/10.17 gp3/10.17-bcl2-gp4-2.5s.tif]

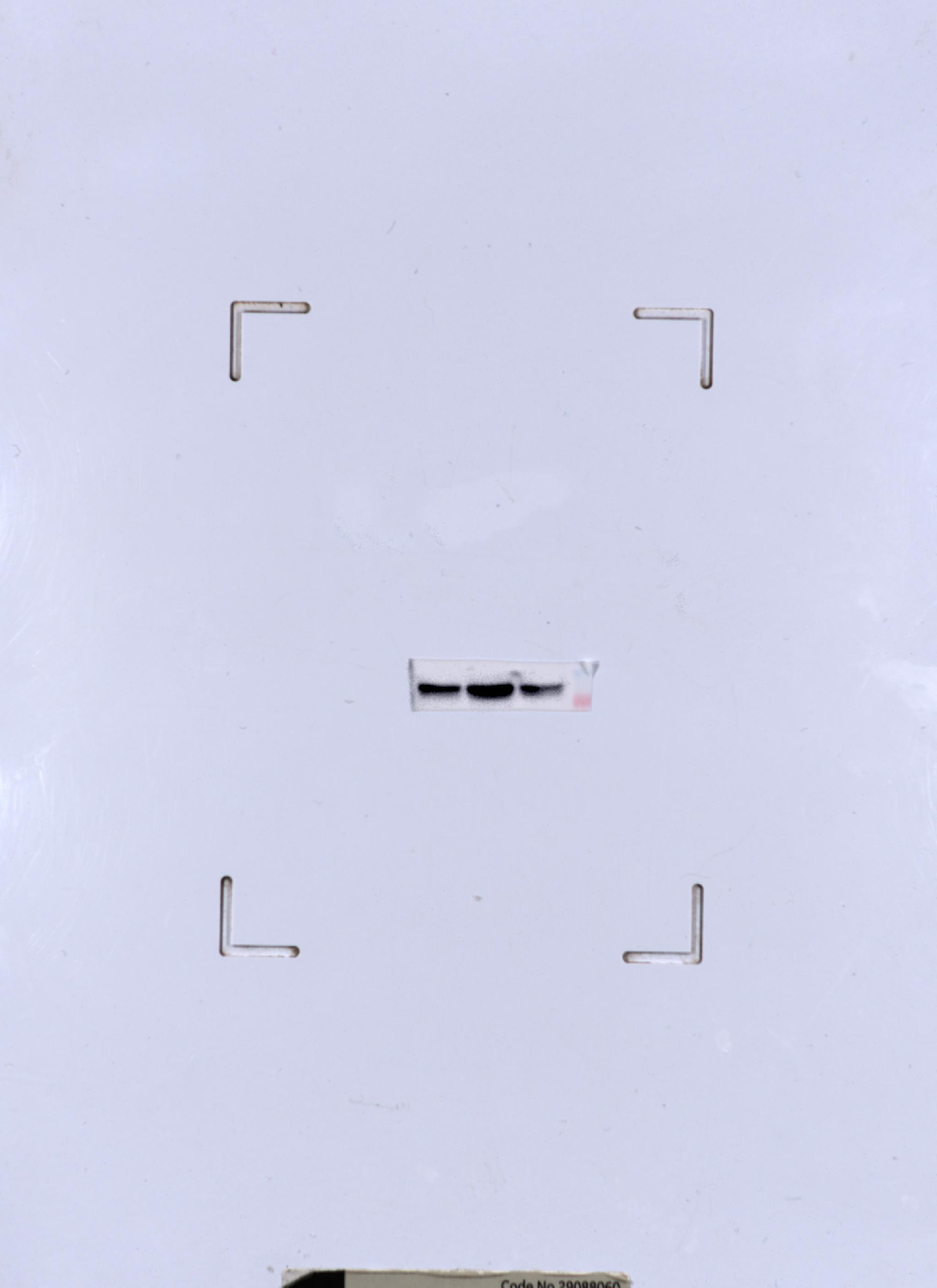

Supplement: S2 Raw Images — (ZIP) [file pone.0322653.s002.zip › S1_raw_images2-wb data/10.17 gp3/10.17-STAT3-gp3-0.4s.tif]

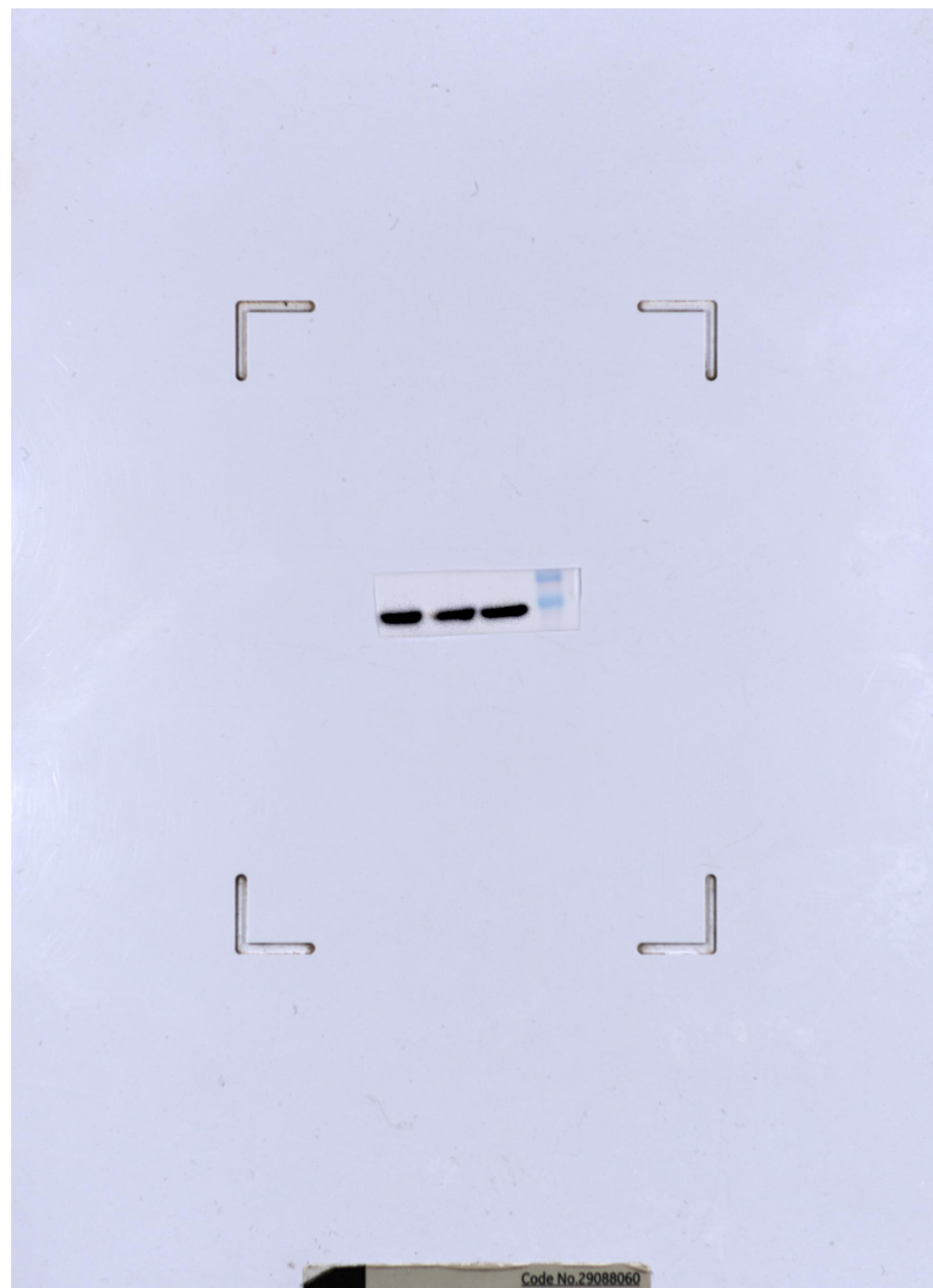

Supplement: S2 Raw Images — (ZIP) [file pone.0322653.s002.zip › S1_raw_images2-wb data/10.17 gp4/10.17-内参-gp4-0.2s.pdf]

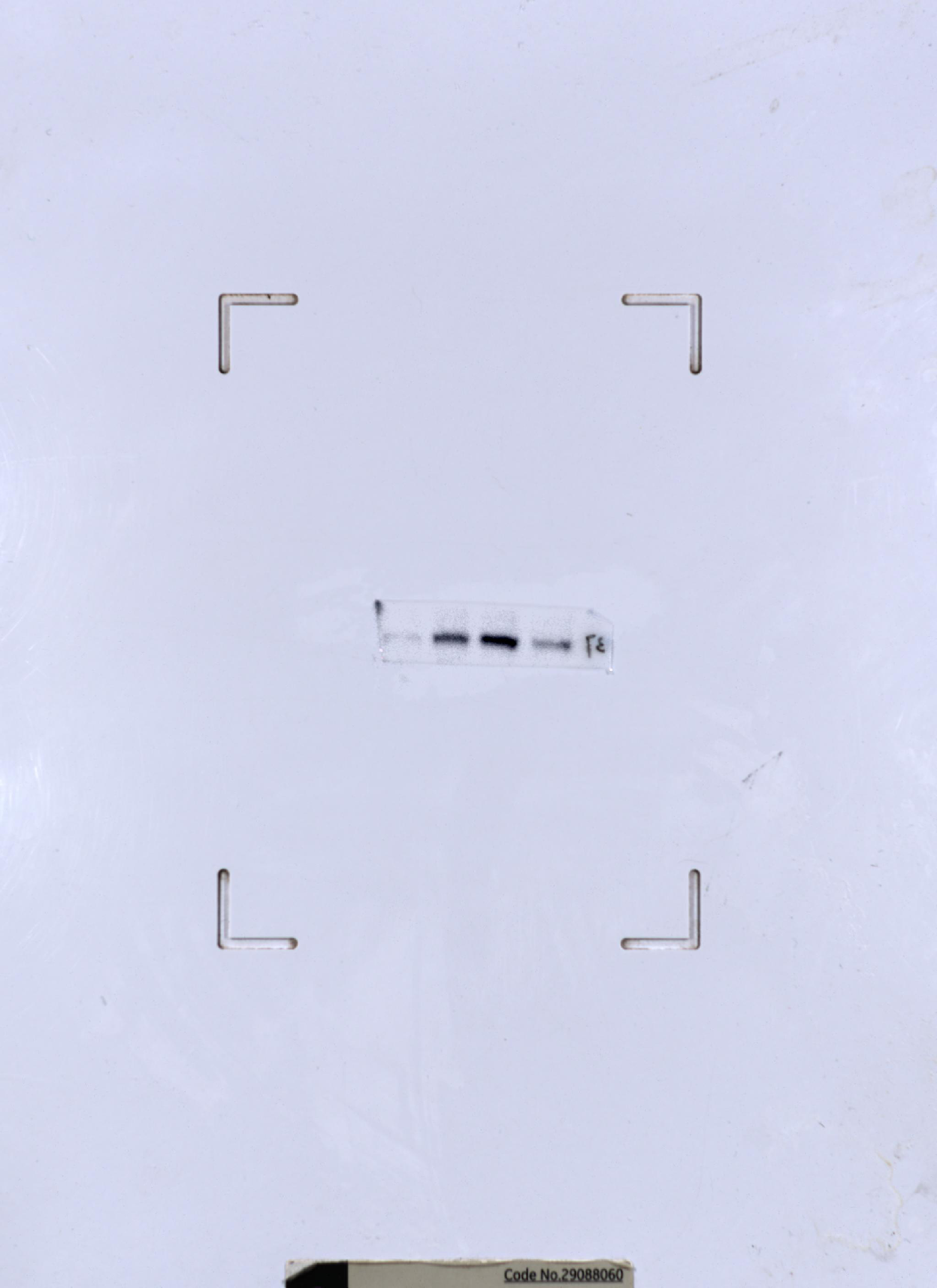

Supplement: S2 Raw Images — (ZIP) [file pone.0322653.s002.zip › S1_raw_images2-wb data/11.20 gp3/11.20-JAK2-gp3-1s.tif]

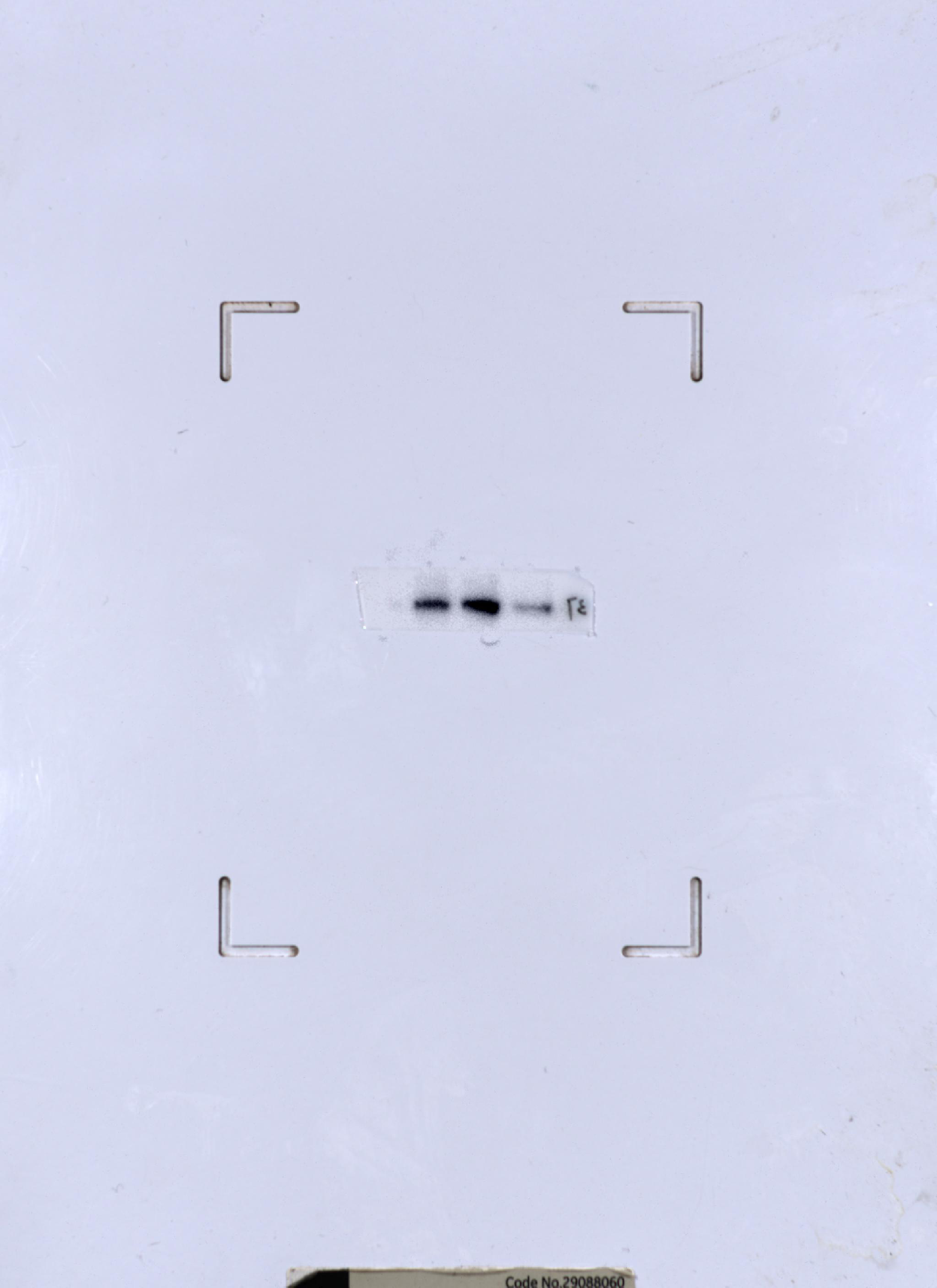

Supplement: S2 Raw Images — (ZIP) [file pone.0322653.s002.zip › S1_raw_images2-wb data/11.20 gp3/11.20-pJAK2-gp3-5S.tif]

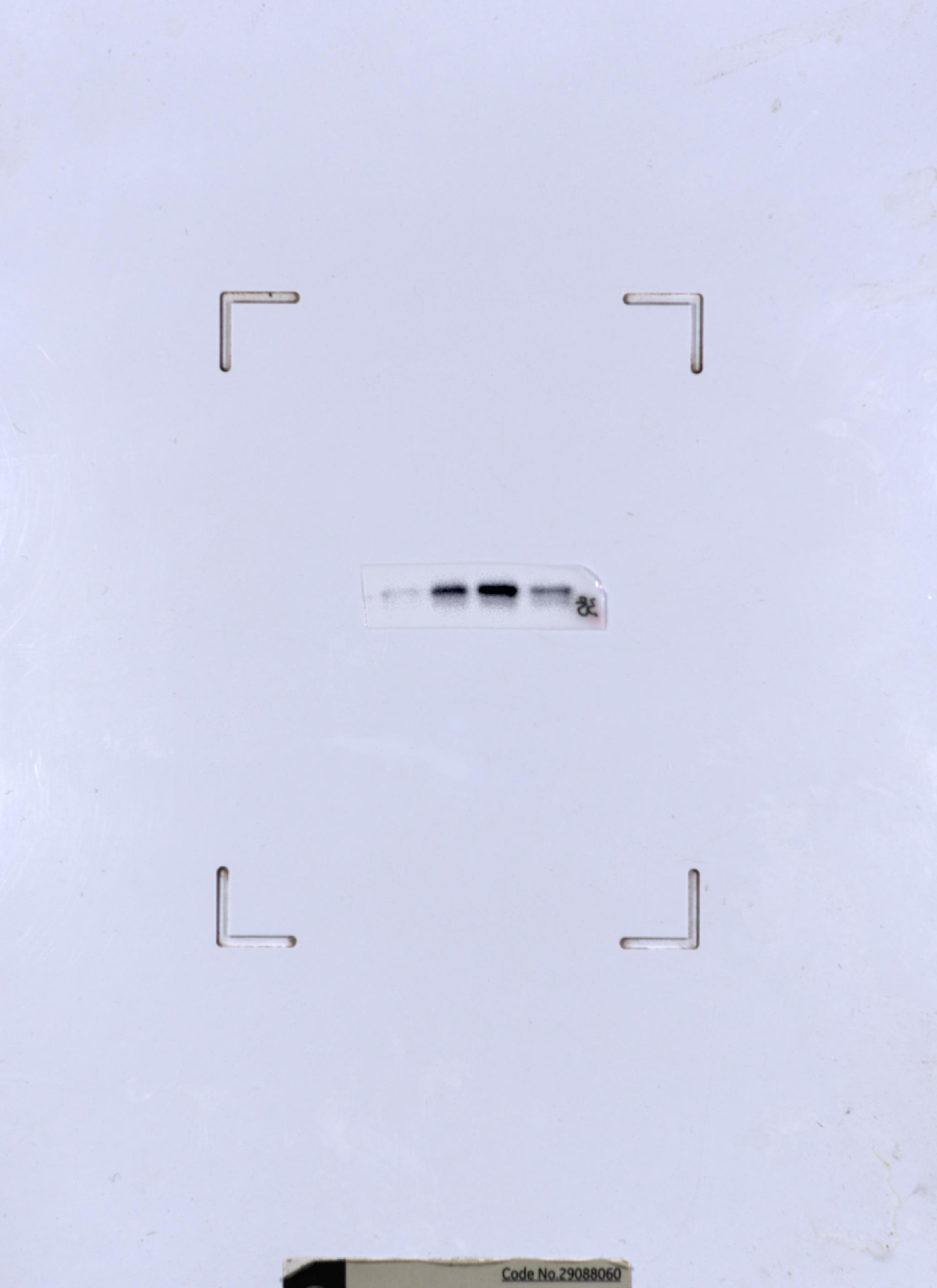

Supplement: S2 Raw Images — (ZIP) [file pone.0322653.s002.zip › S1_raw_images2-wb data/11.20 gp3/11.20-pSTAT3-gp3-0.5S.tif]

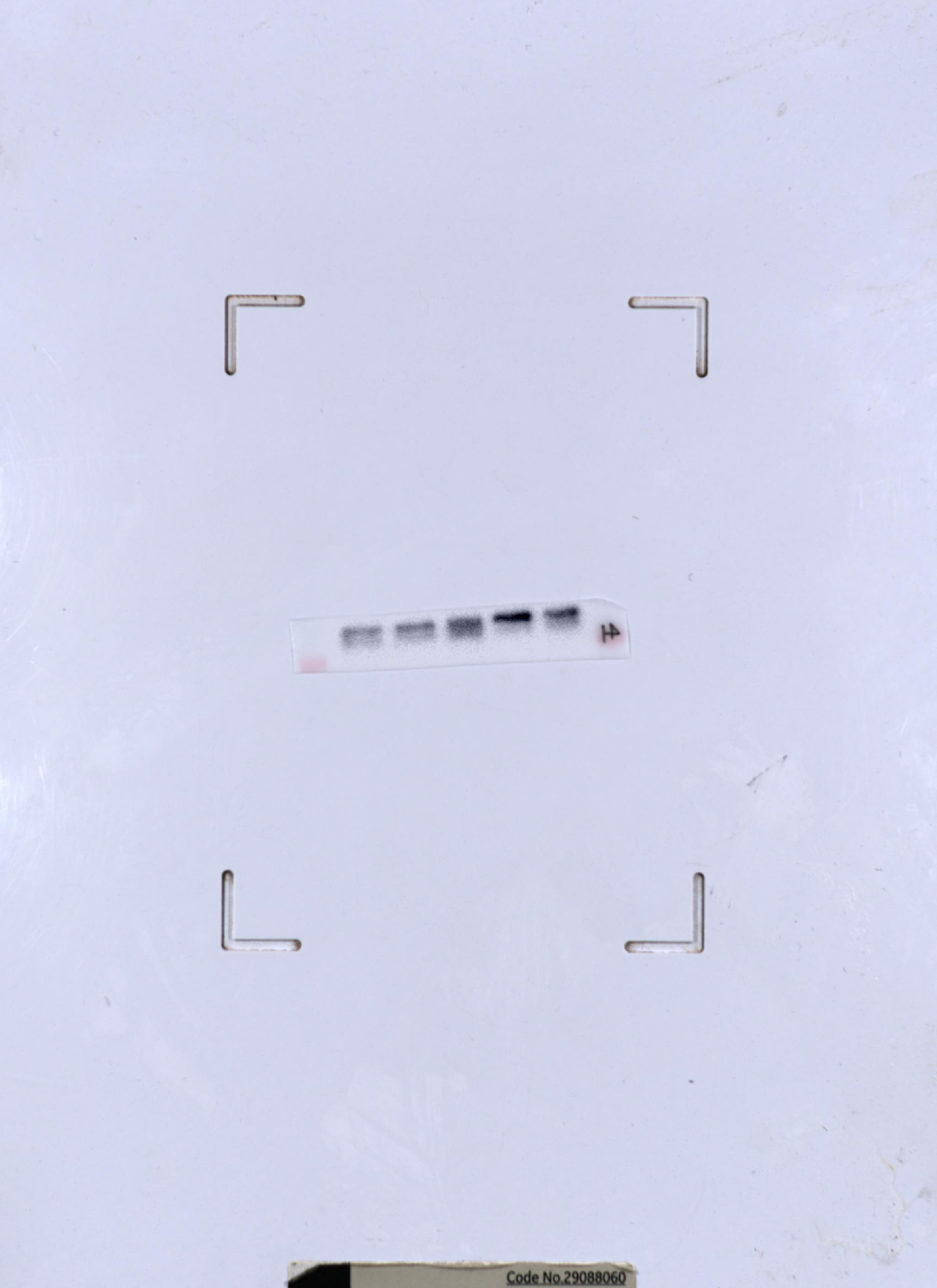

Supplement: S2 Raw Images — (ZIP) [file pone.0322653.s002.zip › S1_raw_images2-wb data/11.20 gp3/11.20-STAT3-gp3-0.1s.tif]

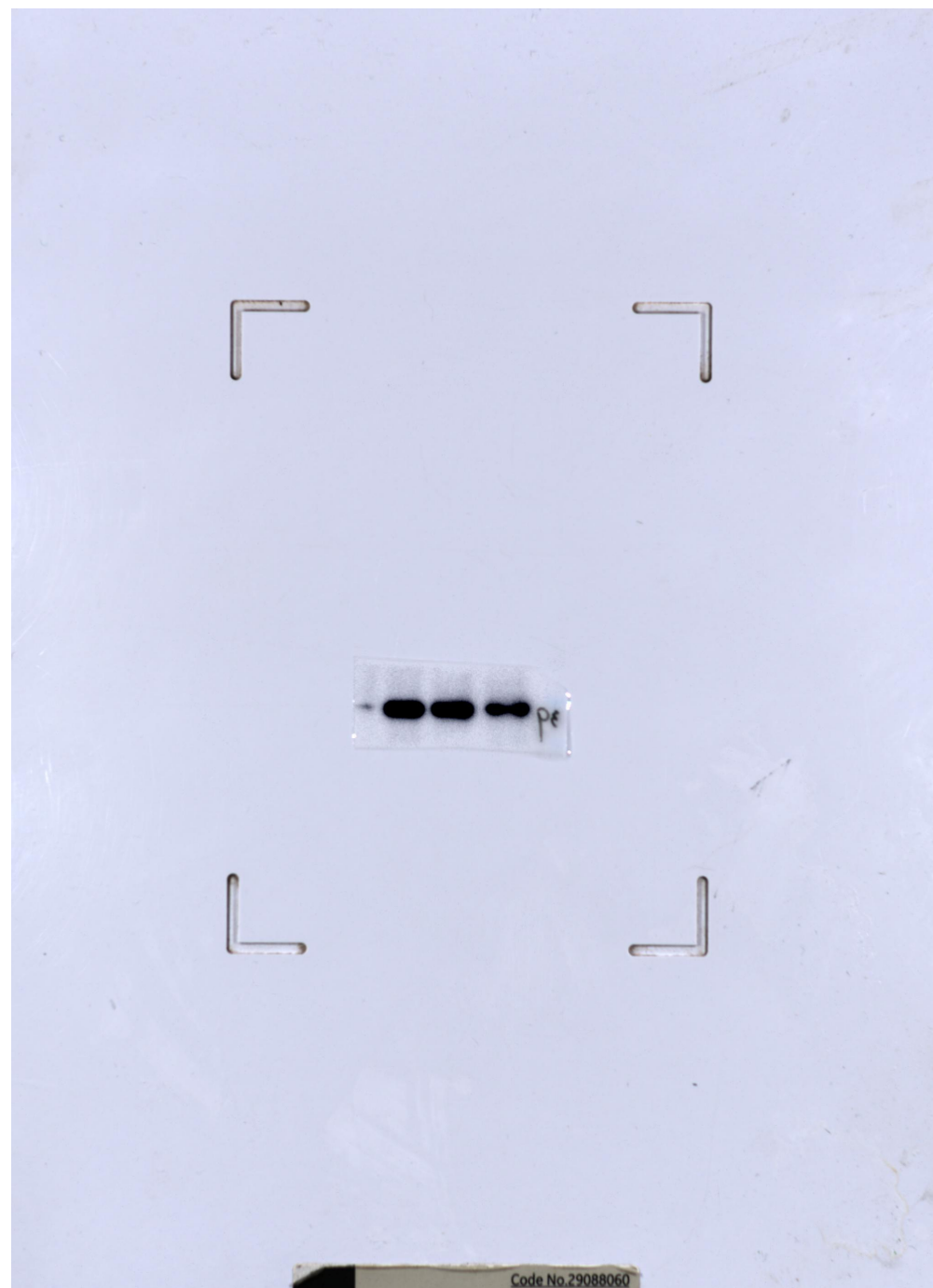

Supplement: S2 Raw Images — (ZIP) [file pone.0322653.s002.zip › S1_raw_images2-wb data/11.20 gp3/11.20-内参-gp3-2.5s.pdf]

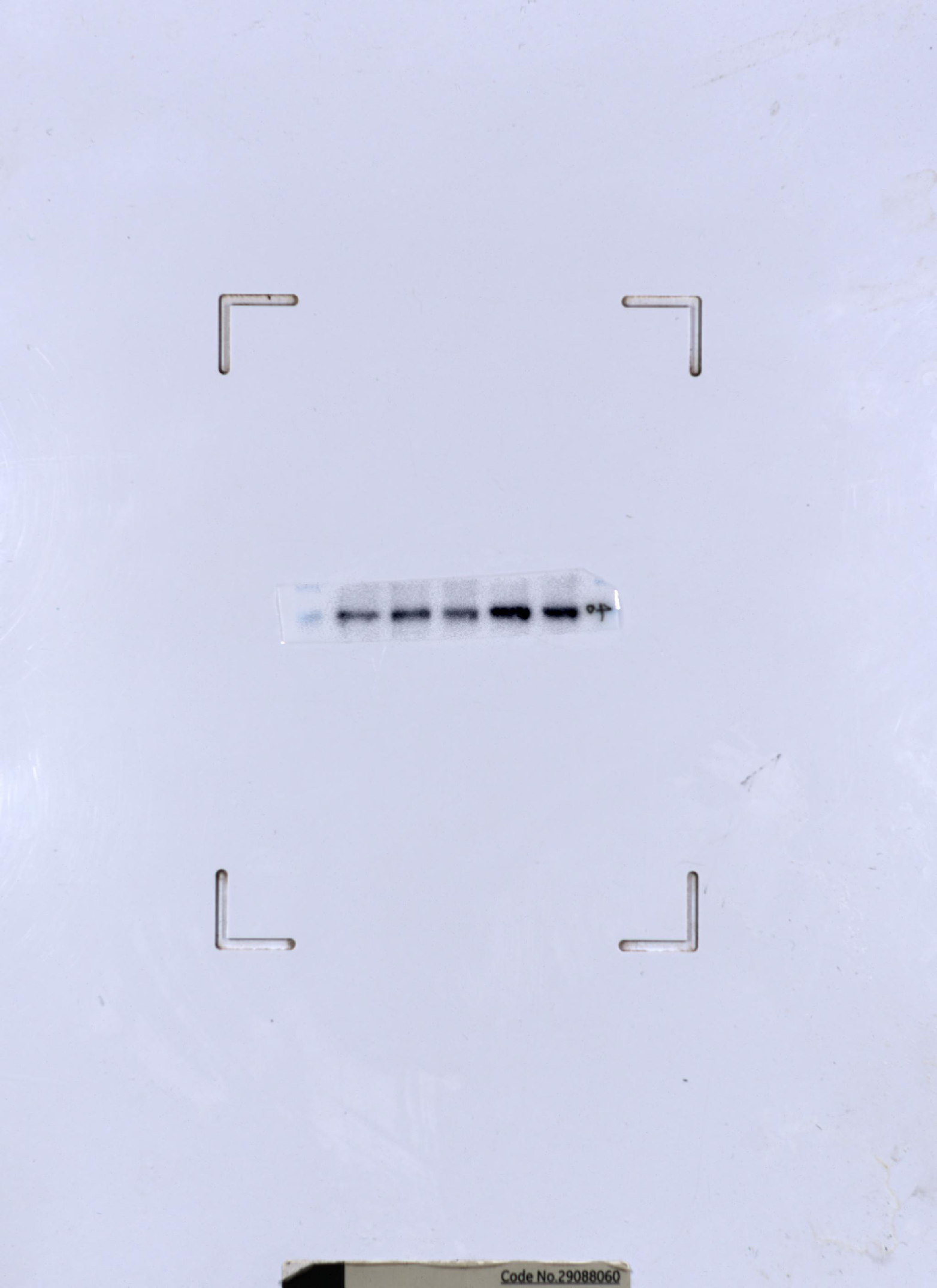

Supplement: S2 Raw Images — (ZIP) [file pone.0322653.s002.zip › S1_raw_images2-wb data/11.20 gp4/11.20-JAK2-gp2-1.5s.tif]

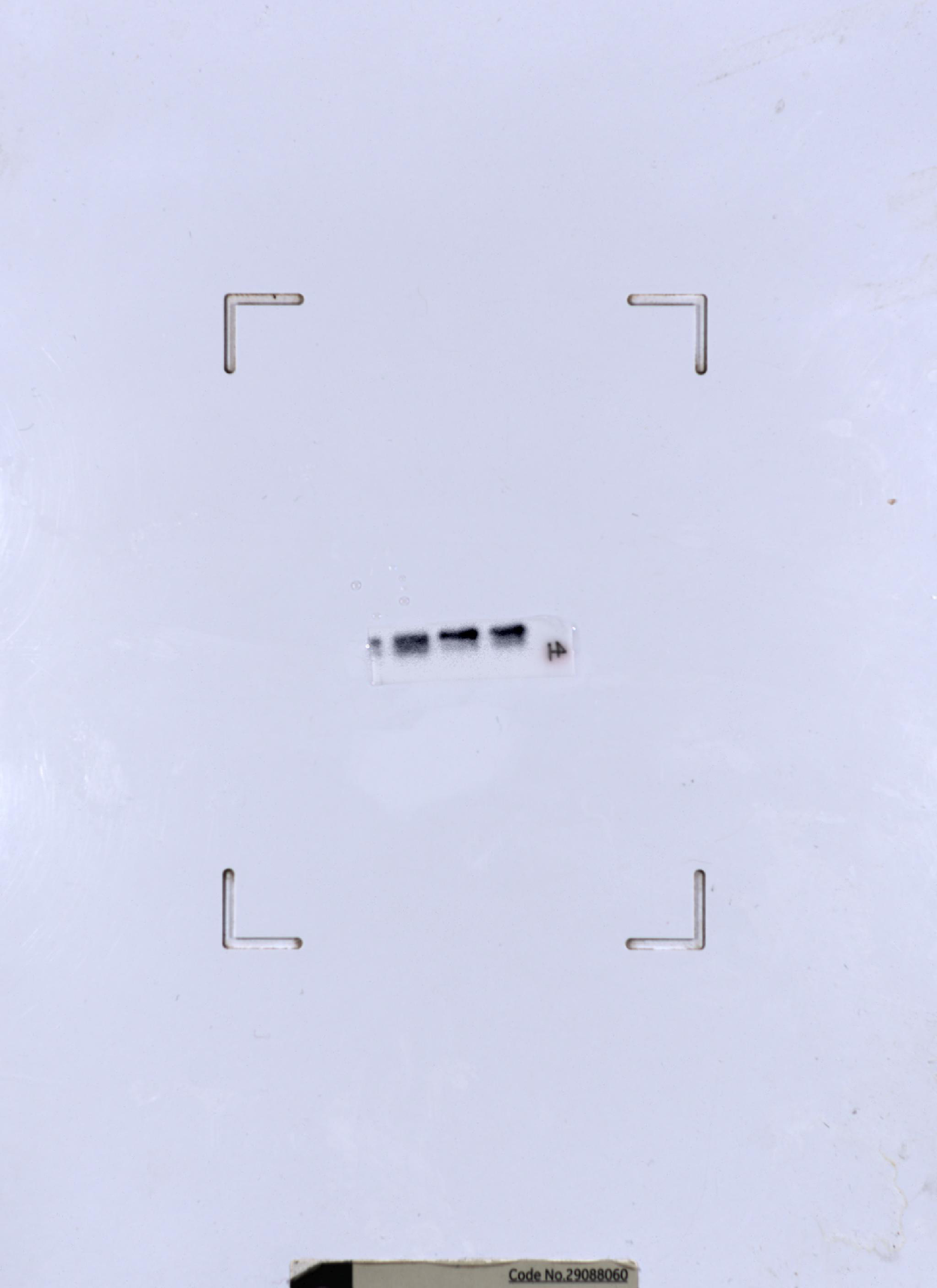

Supplement: S2 Raw Images — (ZIP) [file pone.0322653.s002.zip › S1_raw_images2-wb data/11.20 gp4/11.20-pSTAT3-gp4-0.8S.tif]

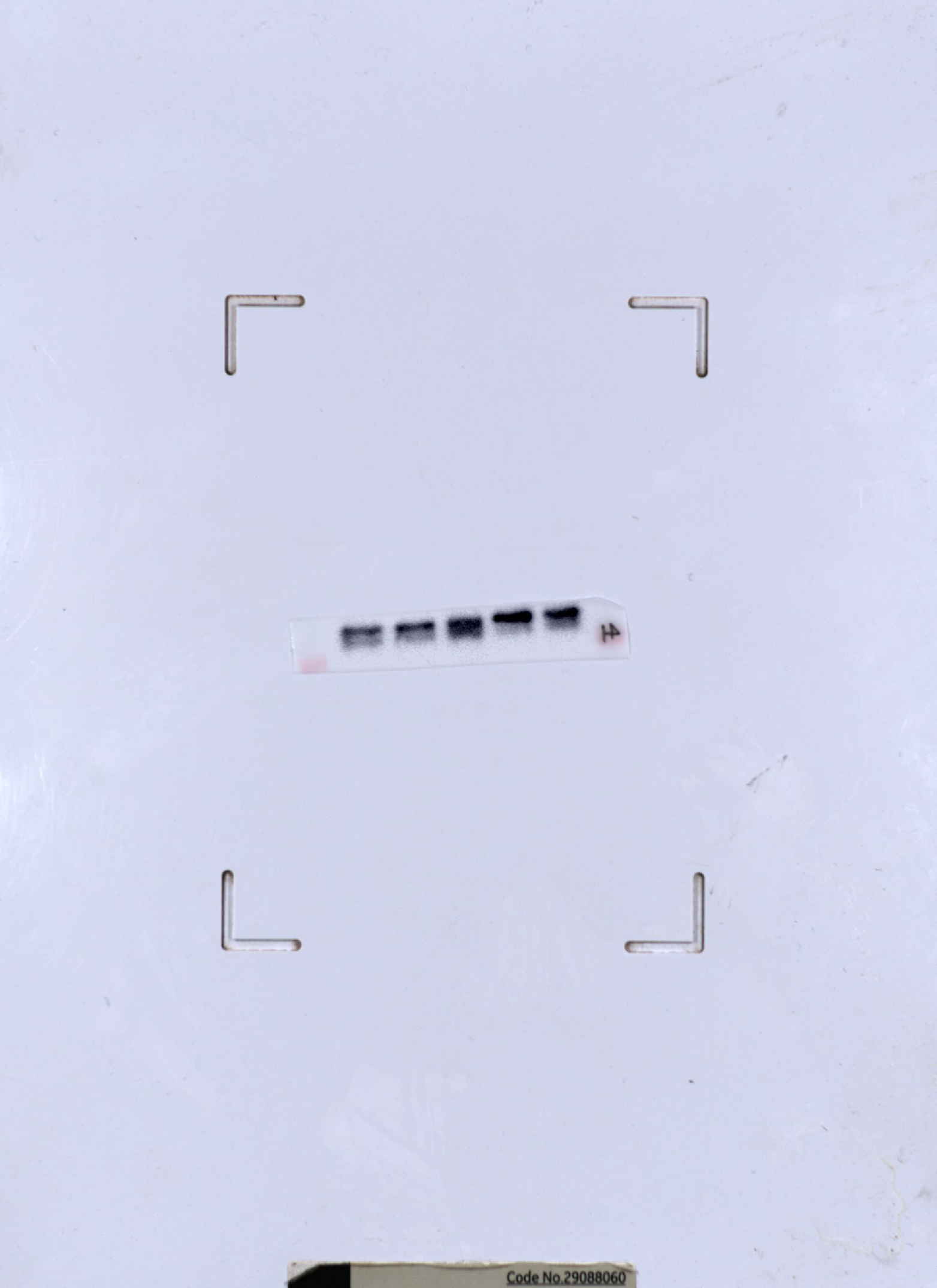

Supplement: S2 Raw Images — (ZIP) [file pone.0322653.s002.zip › S1_raw_images2-wb data/11.20 gp4/11.20-STAT3-gp2-0.2s.tif]

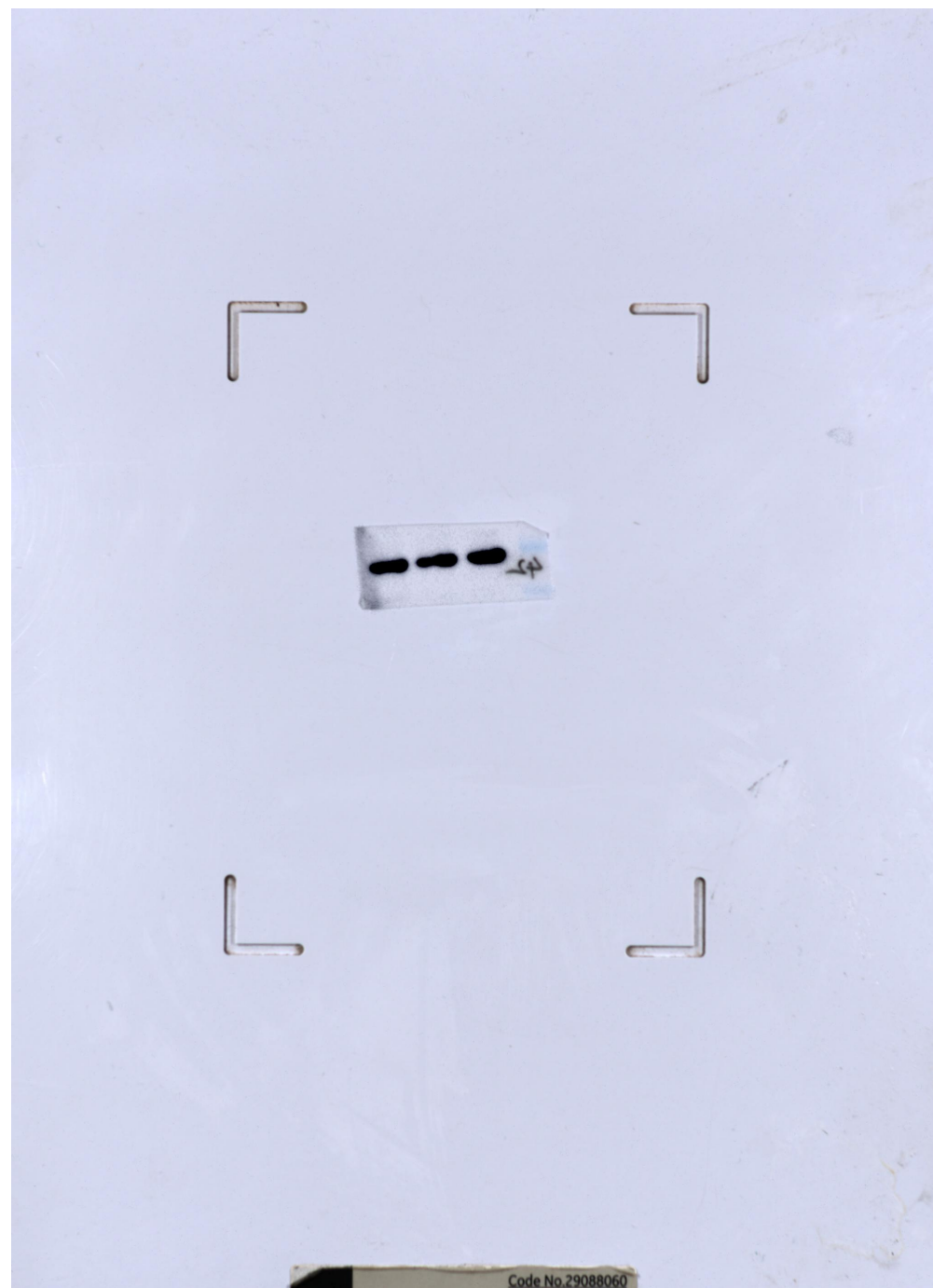

Supplement: S2 Raw Images — (ZIP) [file pone.0322653.s002.zip › S1_raw_images2-wb data/11.20 gp4/11.20-内参-gp4-0.51s.pdf]

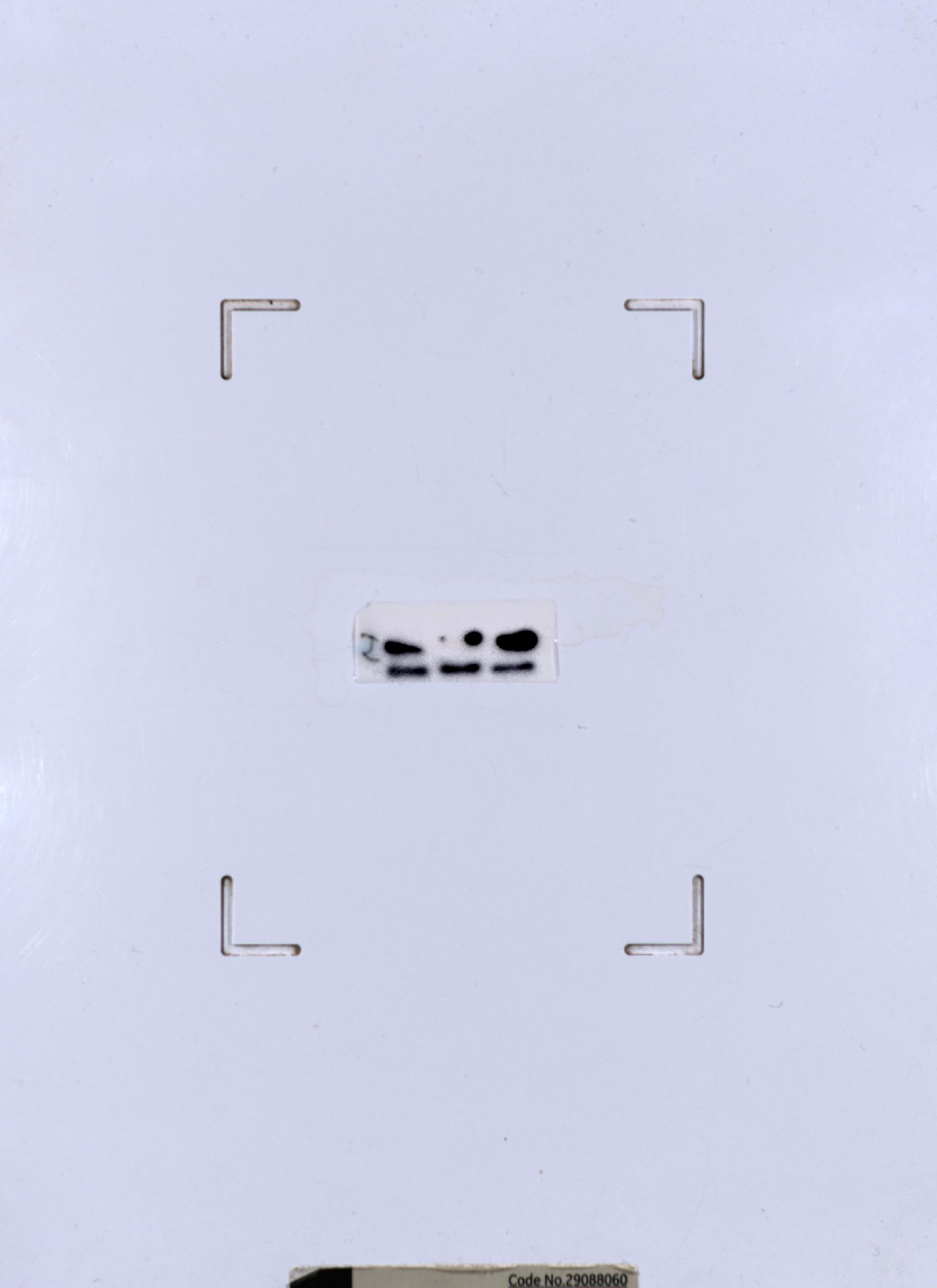

Supplement: S2 Raw Images — (ZIP) [file pone.0322653.s002.zip › S1_raw_images2-wb data/9.10 gp1/9.10-bax-gp1-3s.tif]

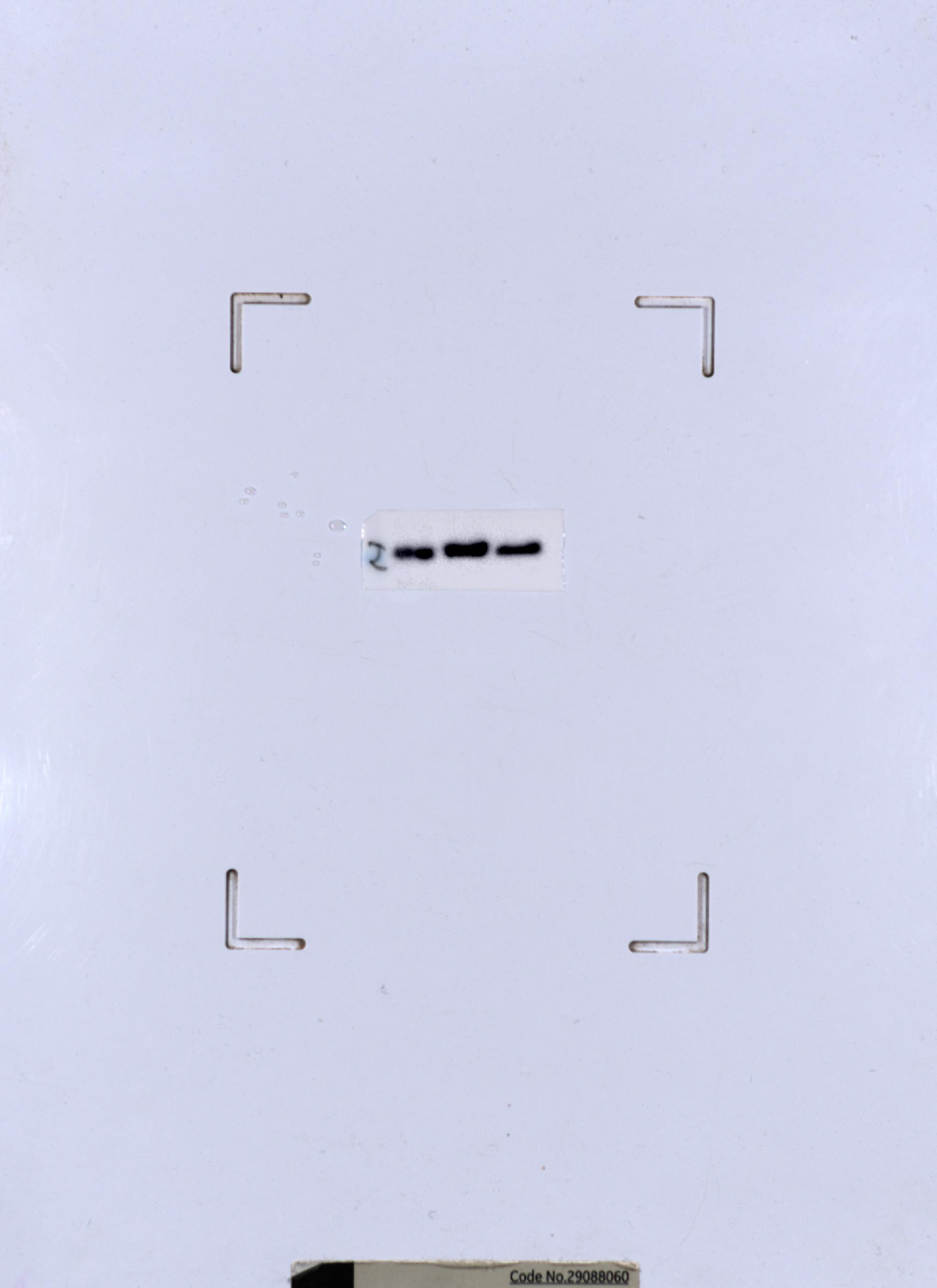

Supplement: S2 Raw Images — (ZIP) [file pone.0322653.s002.zip › S1_raw_images2-wb data/9.10 gp1/9.10-HMGB1-gp1-0.1s.tif]

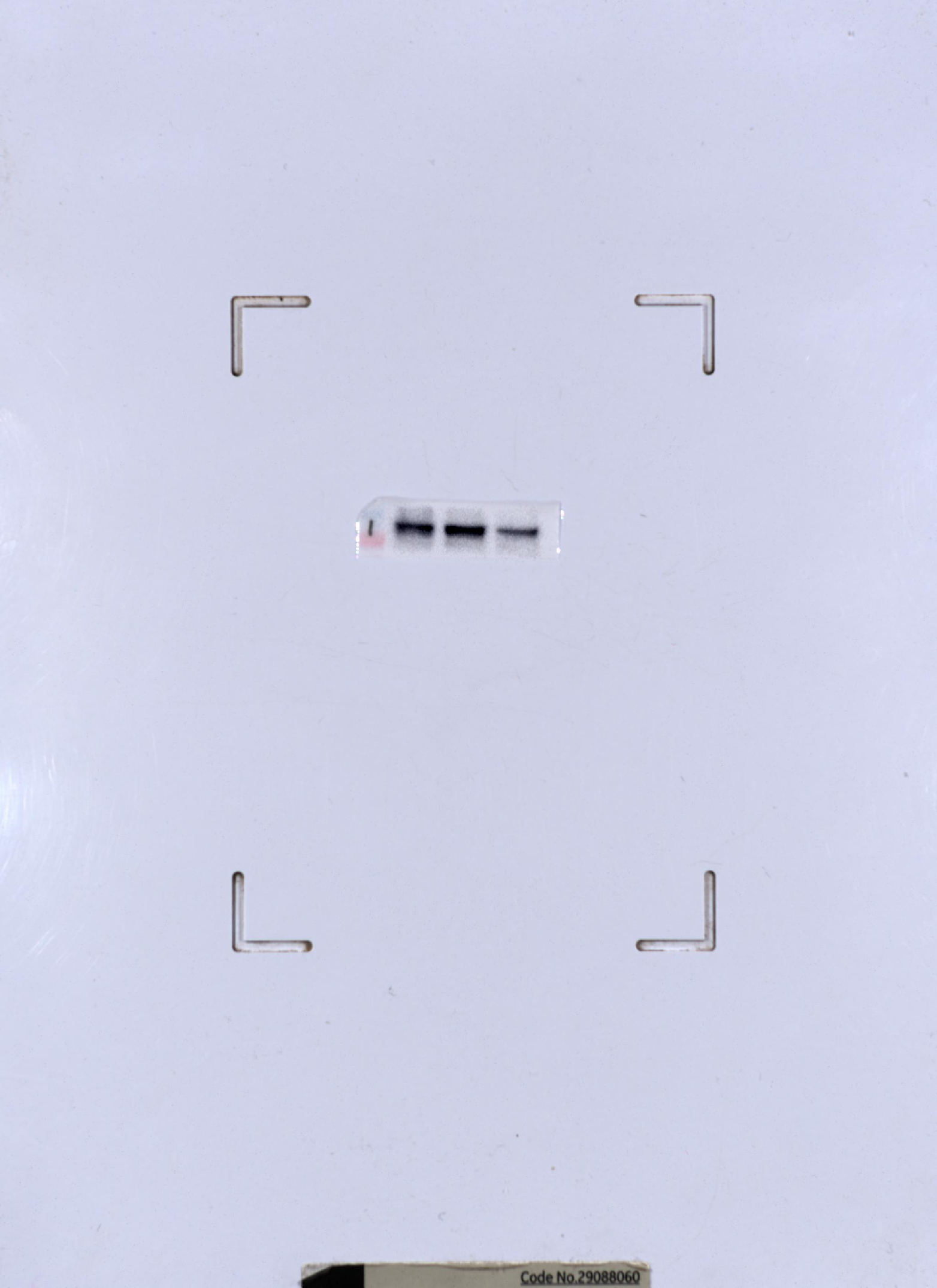

Supplement: S2 Raw Images — (ZIP) [file pone.0322653.s002.zip › S1_raw_images2-wb data/9.10 gp1/9.10-STAT3-gp1-0.2s.tif]

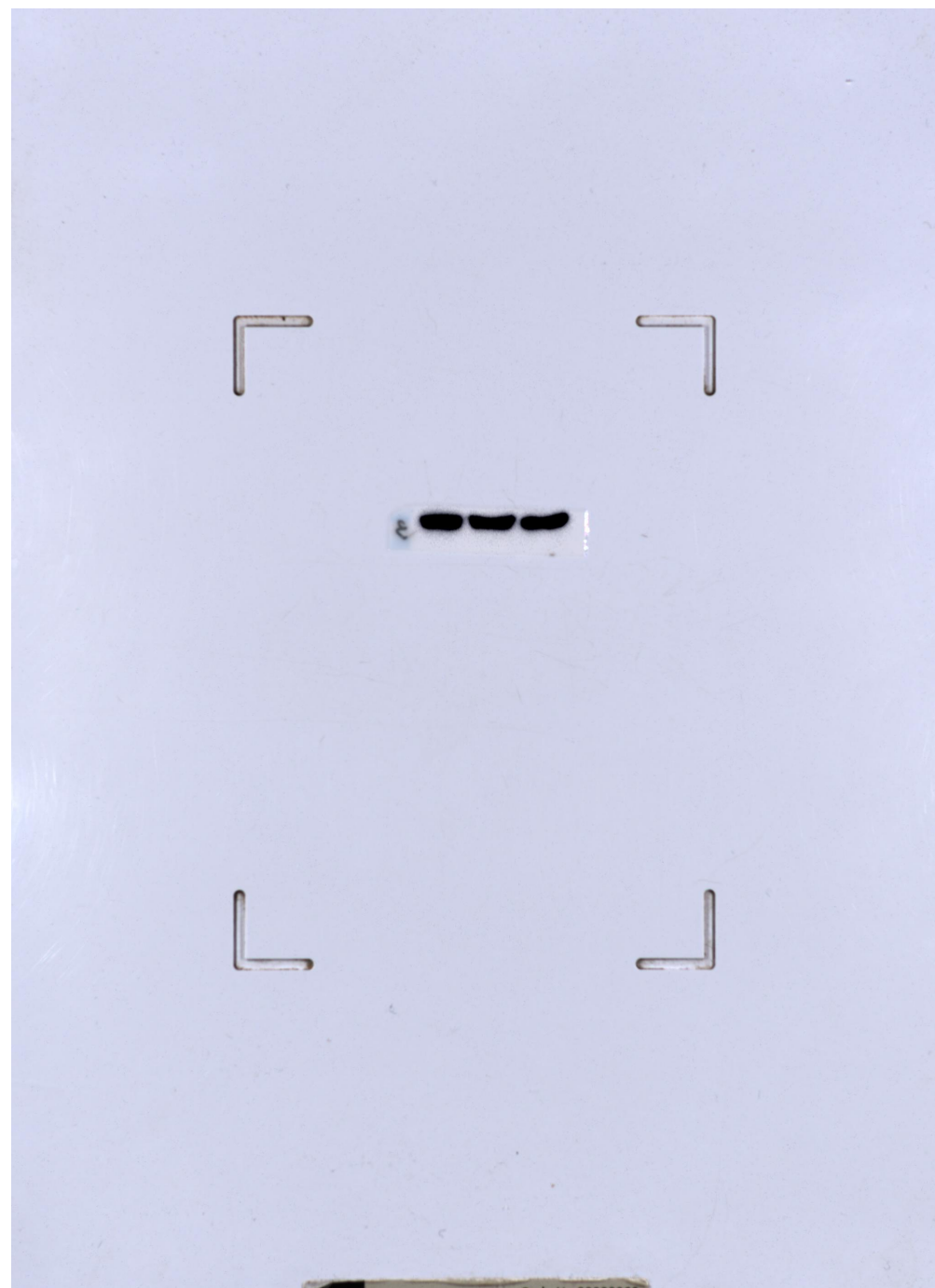

Supplement: S2 Raw Images — (ZIP) [file pone.0322653.s002.zip › S1_raw_images2-wb data/9.10 gp1/9.10-内参-gp1-0.20s.pdf]

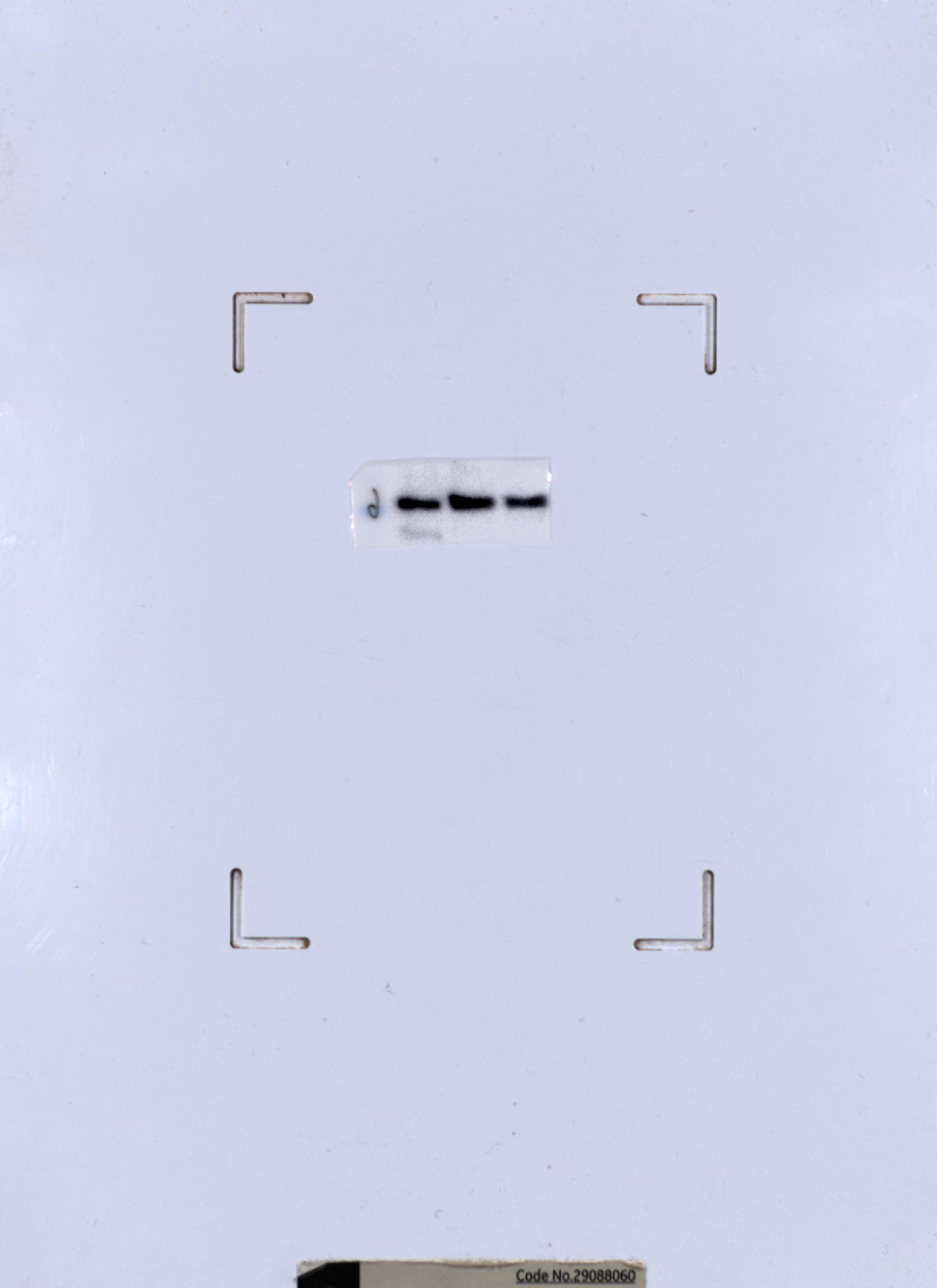

Supplement: S2 Raw Images — (ZIP) [file pone.0322653.s002.zip › S1_raw_images2-wb data/9.10 gp2/9.10-HMGB1-gp2-0.10s.tif]

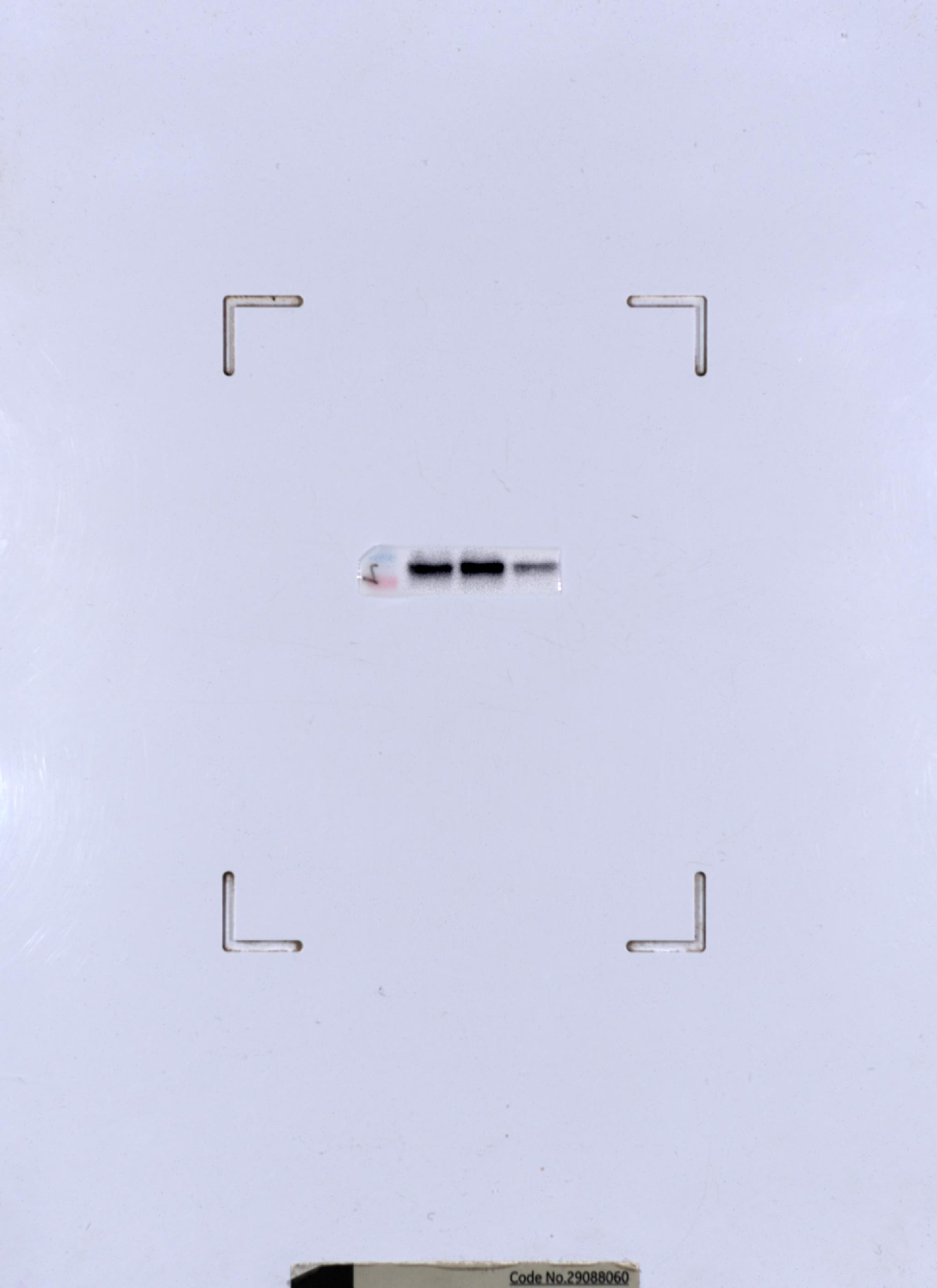

Supplement: S2 Raw Images — (ZIP) [file pone.0322653.s002.zip › S1_raw_images2-wb data/9.10 gp2/9.10-STAT3-gp2-0.1s.tif]

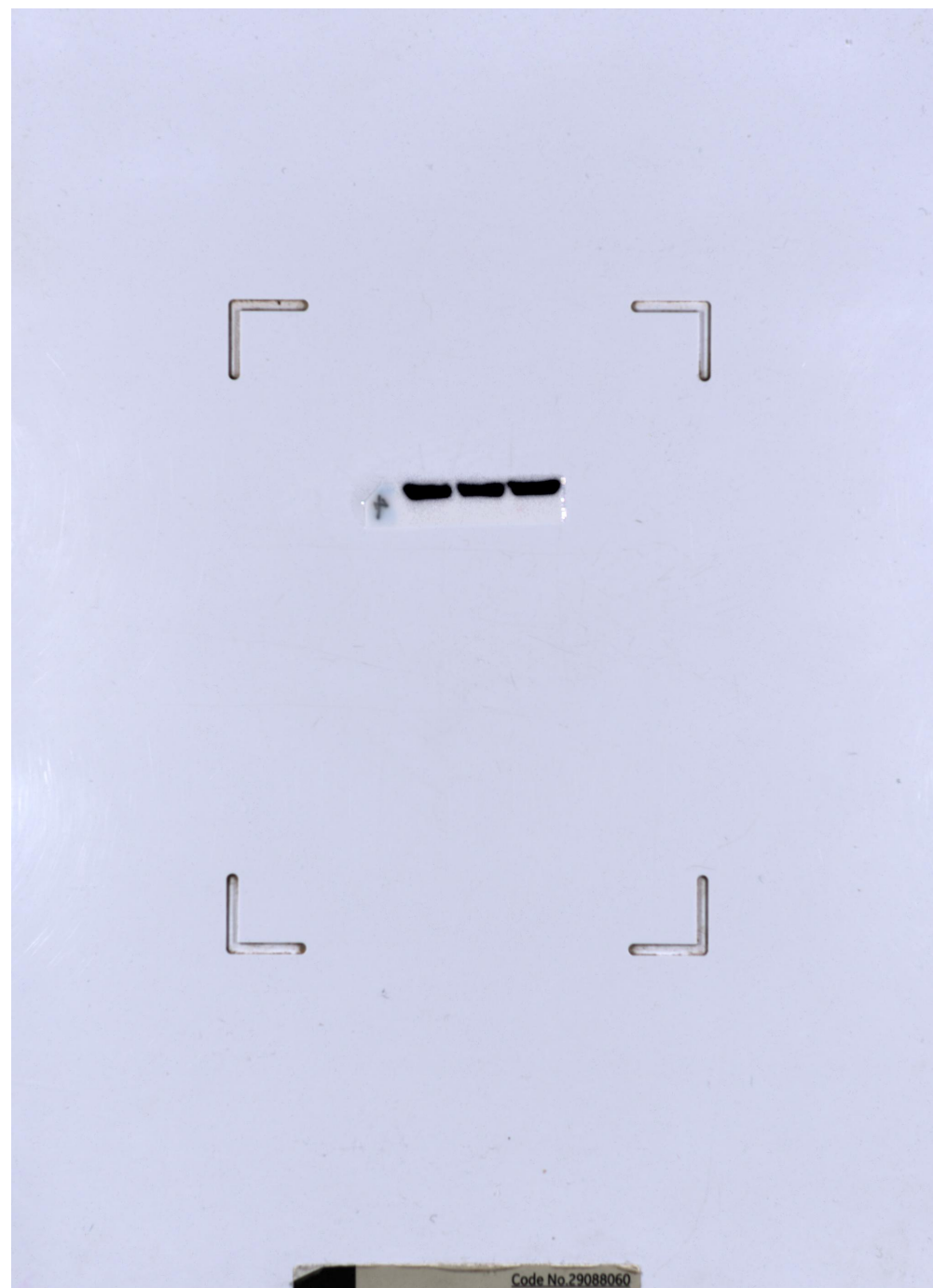

Supplement: S2 Raw Images — (ZIP) [file pone.0322653.s002.zip › S1_raw_images2-wb data/9.10 gp2/9.10-内参-gp2-0.20s.pdf]

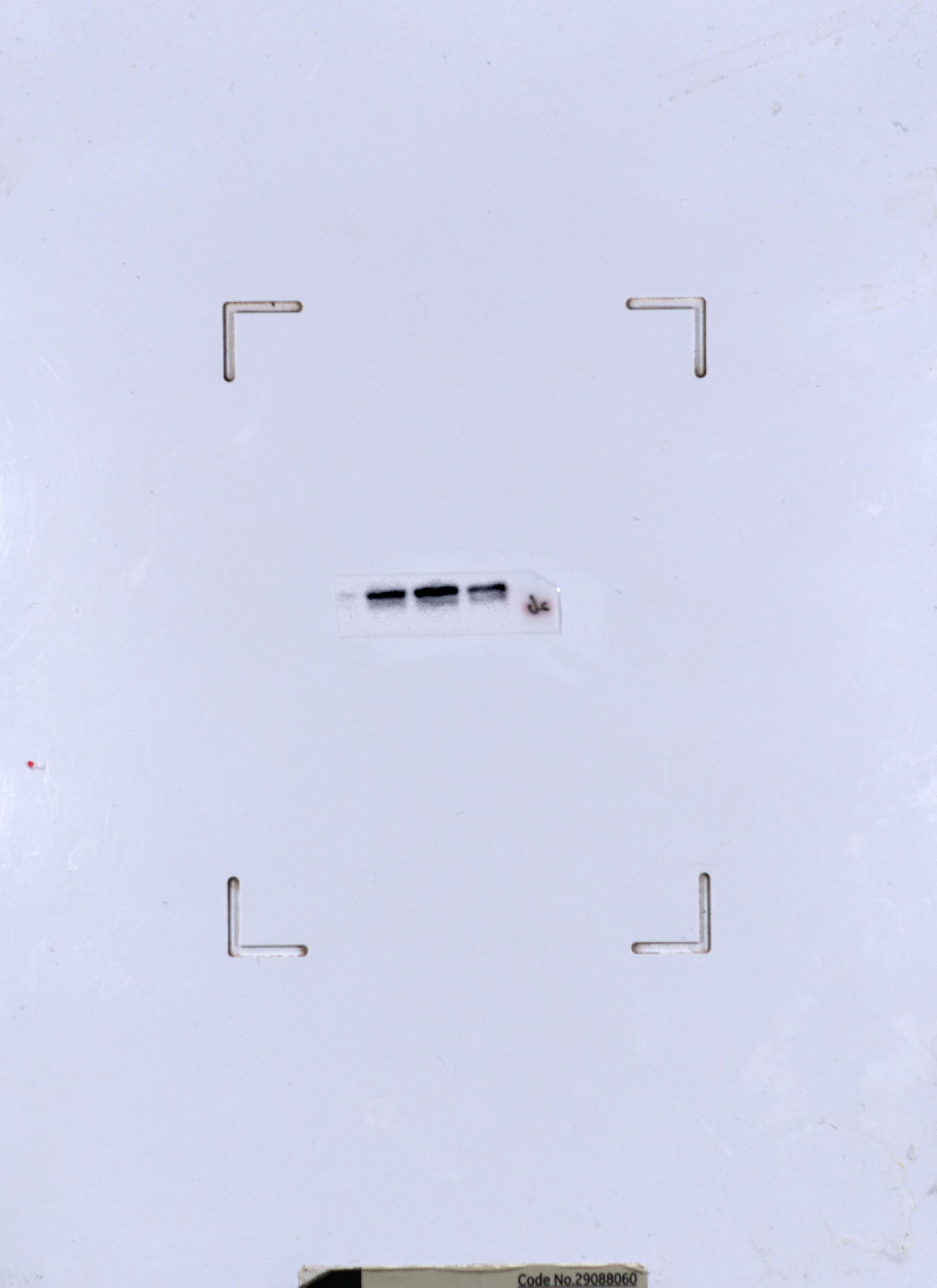

Supplement: S2 Raw Images — (ZIP) [file pone.0322653.s002.zip › S1_raw_images2-wb data/picture/11.17-stat3-gp3-0.2s.tif]

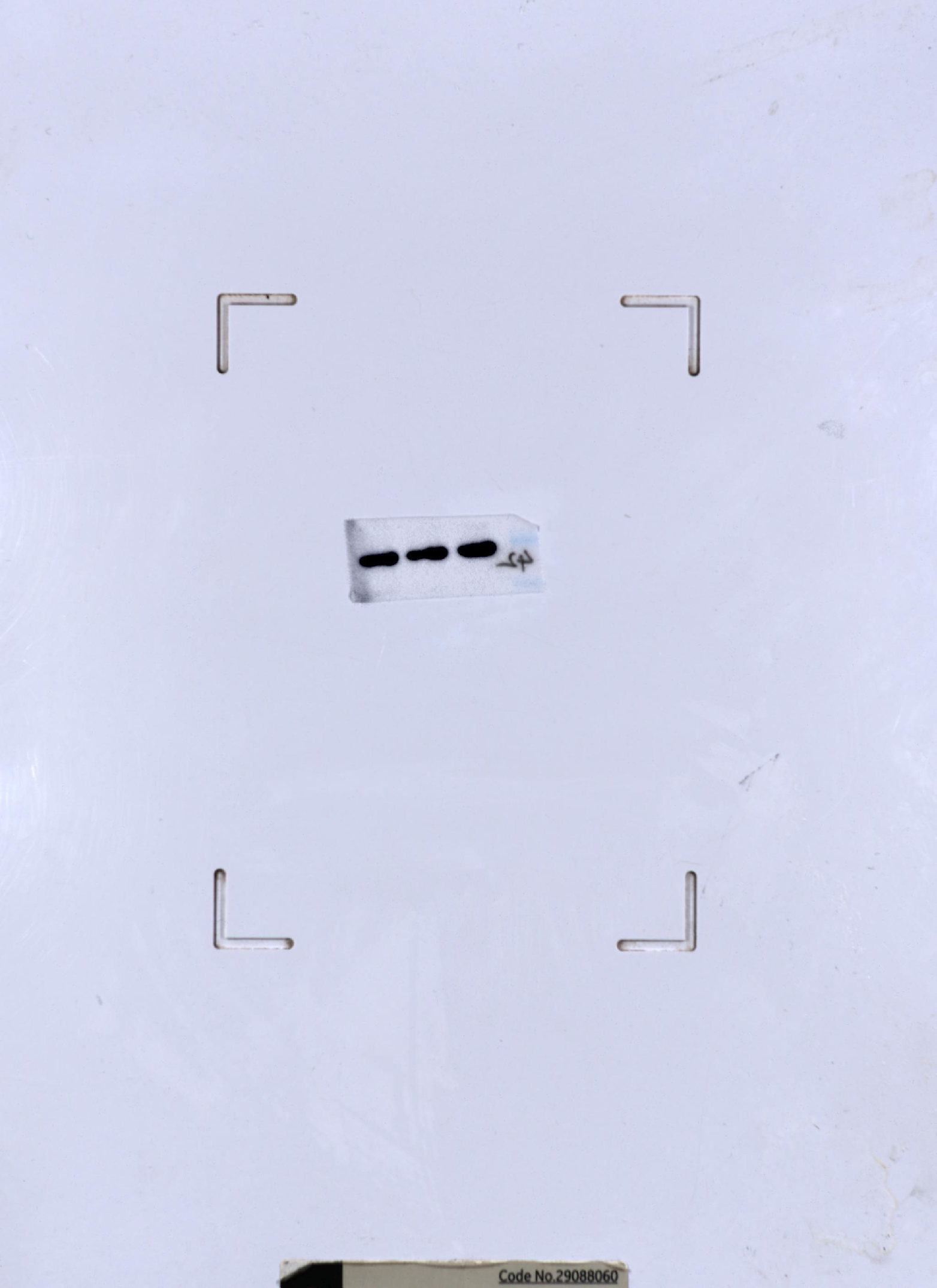

Supplement: S2 Raw Images — (ZIP) [file pone.0322653.s002.zip › S1_raw_images2-wb data/picture/11.20-reference -gp4-0.51s.tif]

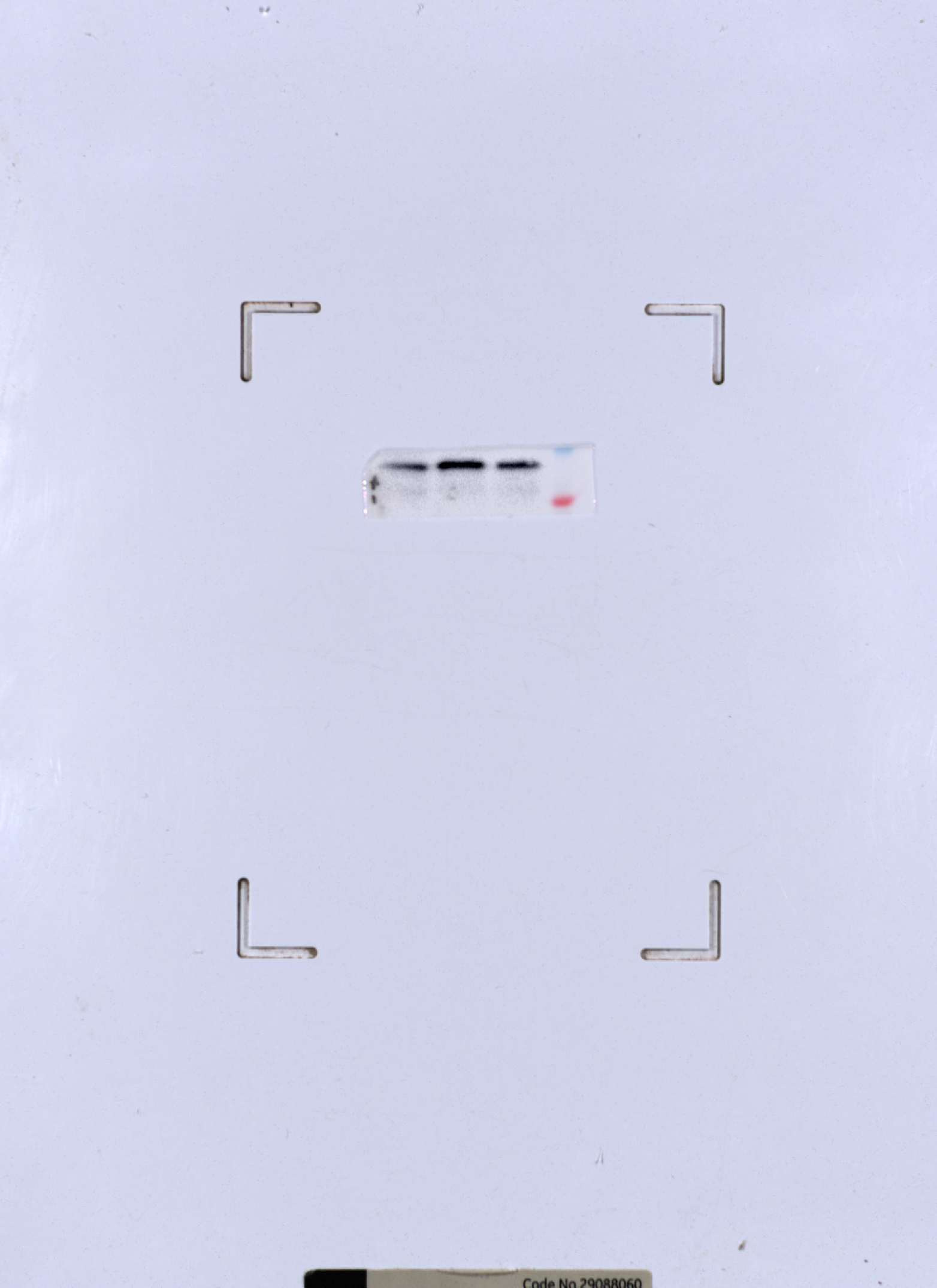

Supplement: S2 Raw Images — (ZIP) [file pone.0322653.s002.zip › S1_raw_images2-wb data/picture/8.8-Bax-S7 CA3 LD7.tif]

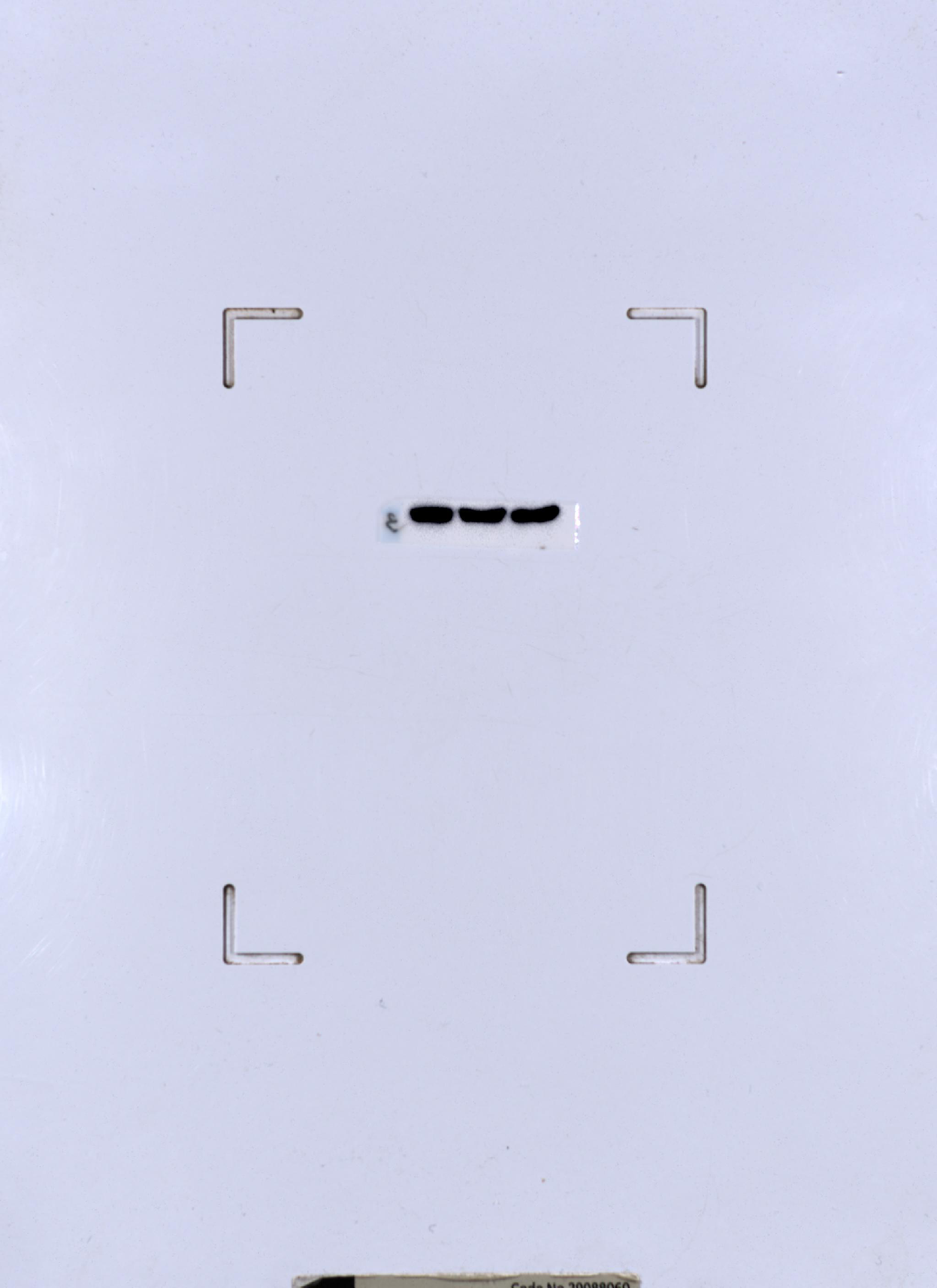

Supplement: S2 Raw Images — (ZIP) [file pone.0322653.s002.zip › S1_raw_images2-wb data/picture/9.10-reference -gp1-0.20s.tif]

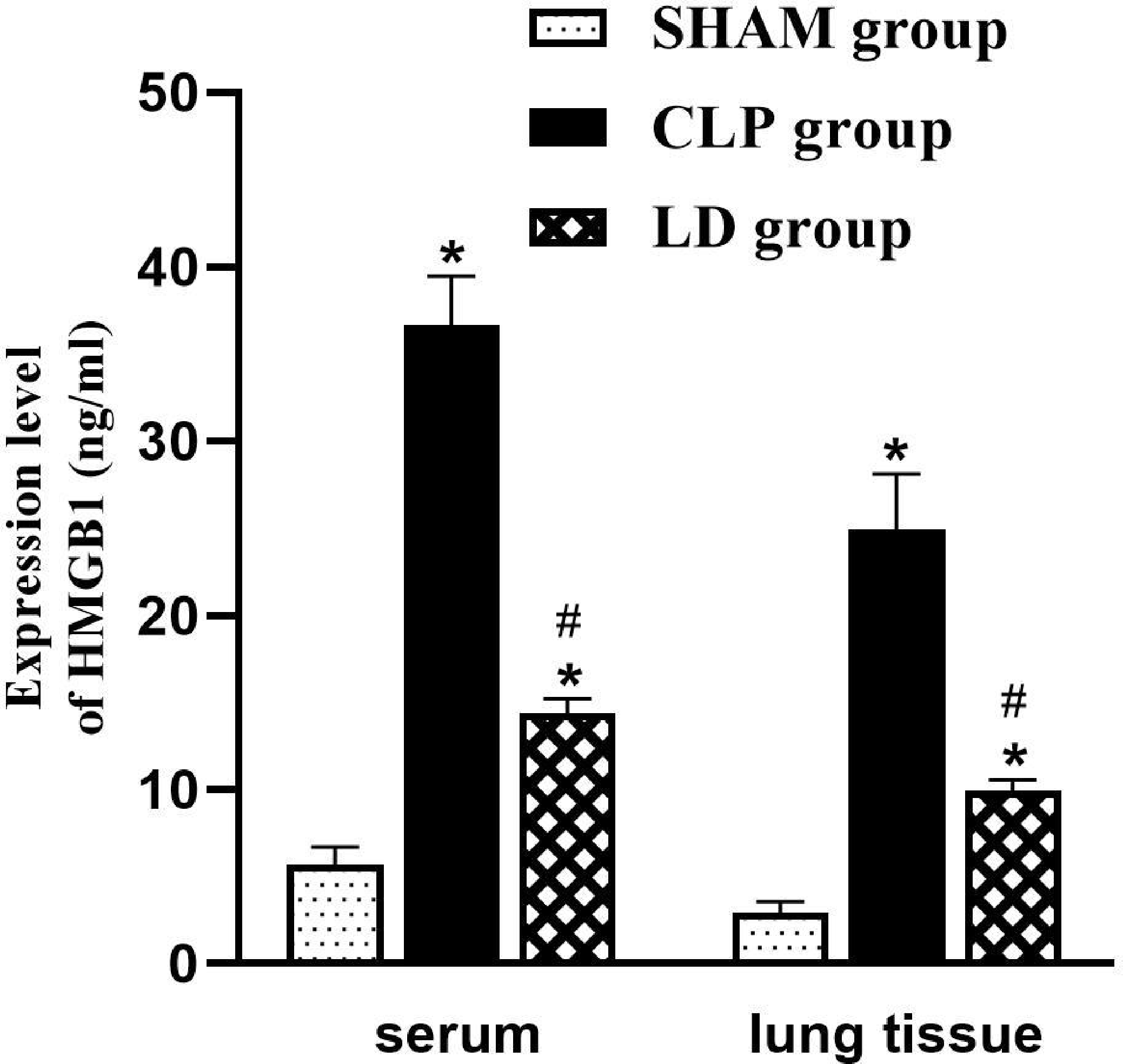

Supplement: S2 Raw Images — (ZIP) [file pone.0322653.s002.zip › S1_raw_images2-wb data/picture/HMGB1.tif]

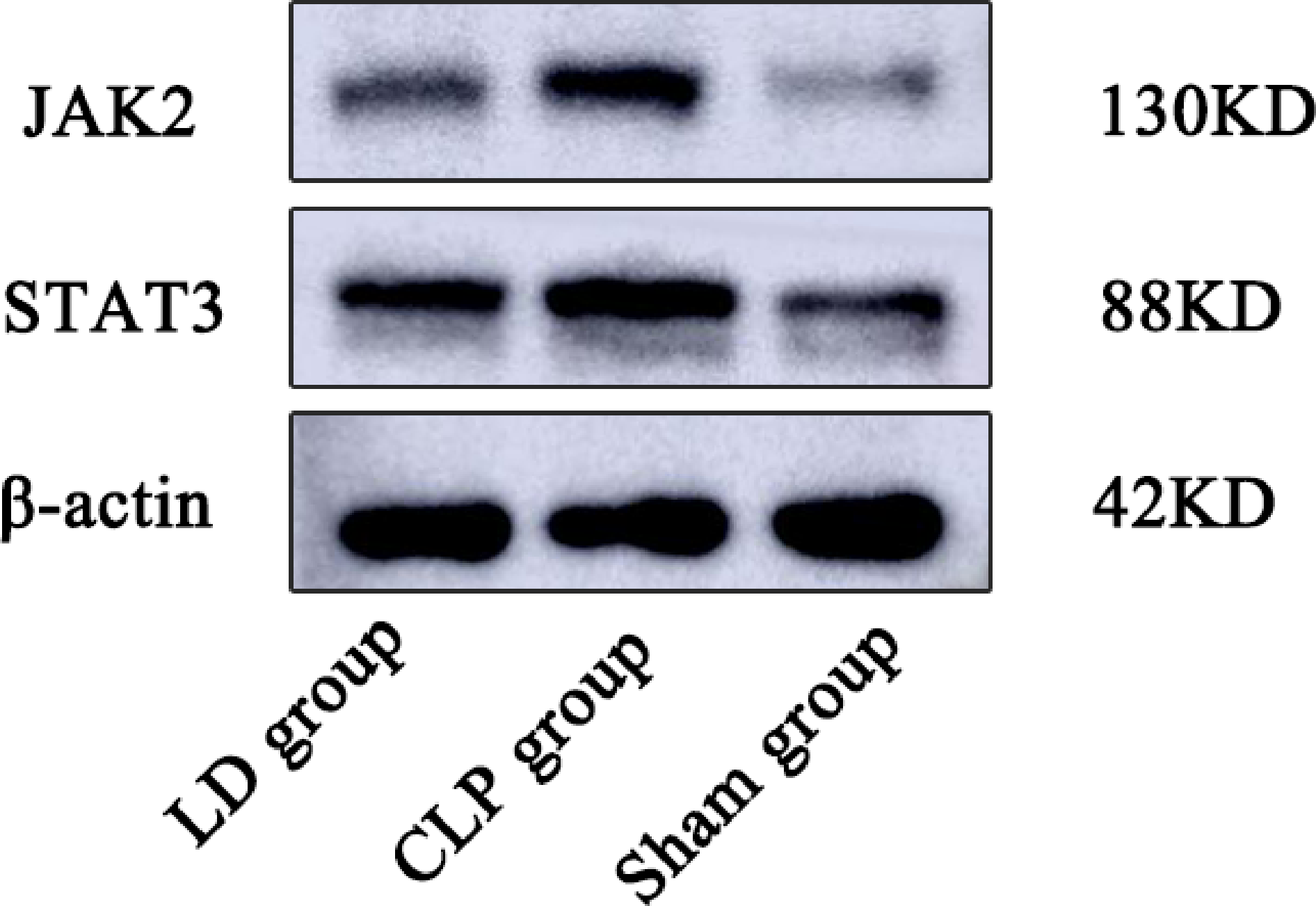

Supplement: S2 Raw Images — (ZIP) [file pone.0322653.s002.zip › S1_raw_images2-wb data/picture/macromolecule1.tif]

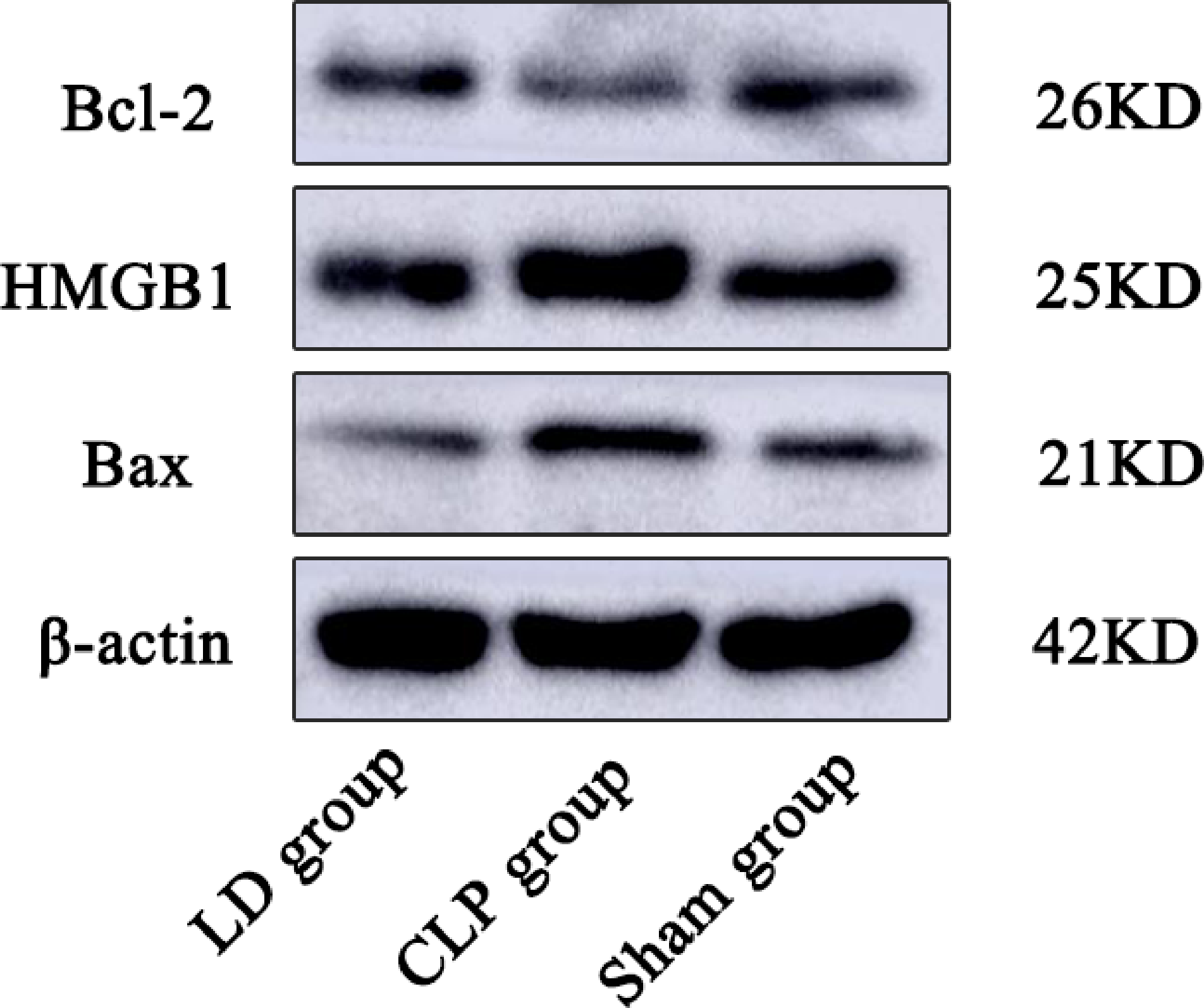

Supplement: S2 Raw Images — (ZIP) [file pone.0322653.s002.zip › S1_raw_images2-wb data/picture/micromolecule2.tif]

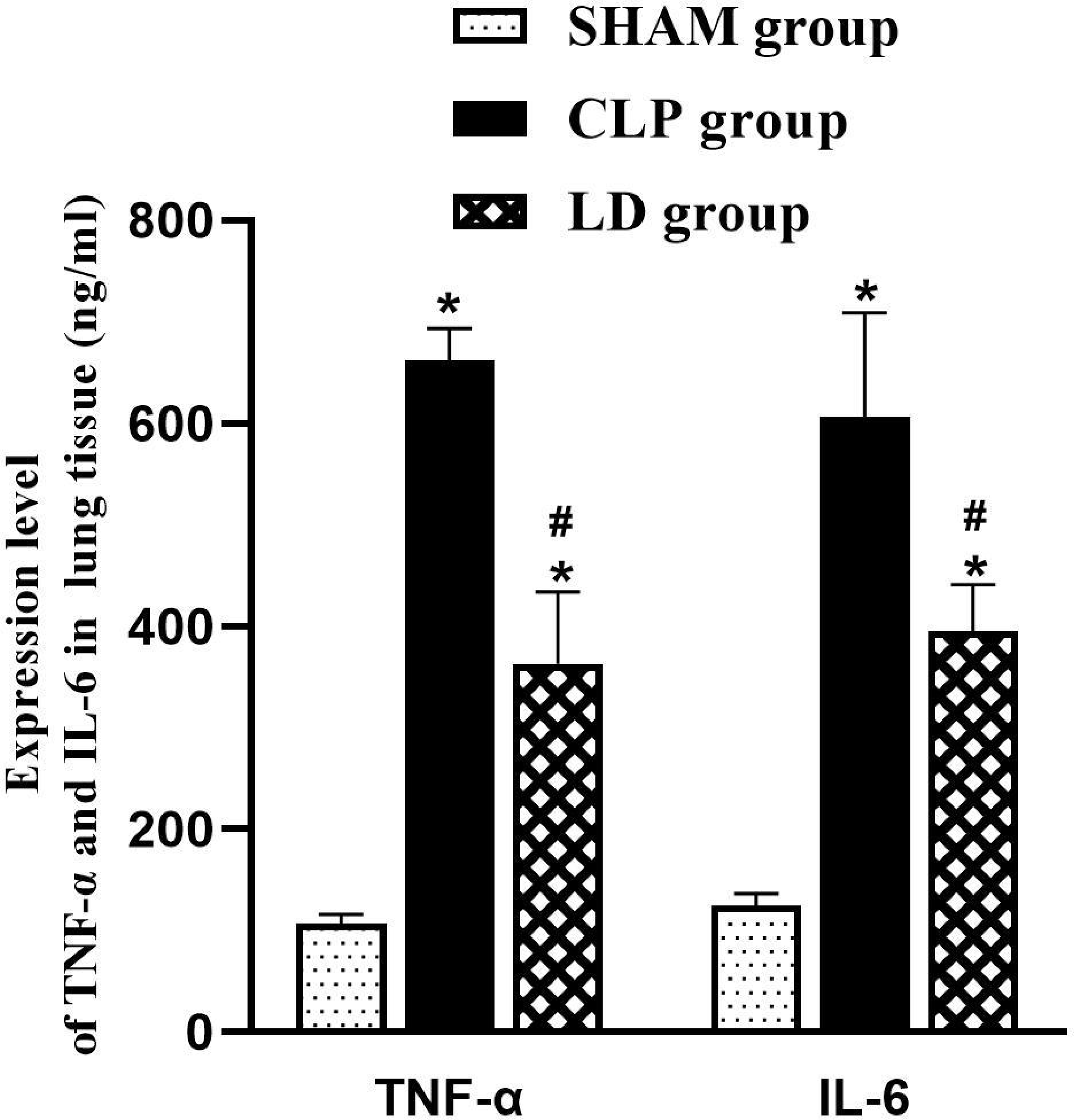

Supplement: S2 Raw Images — (ZIP) [file pone.0322653.s002.zip › S1_raw_images2-wb data/picture/TNF-a IL-6.tif]

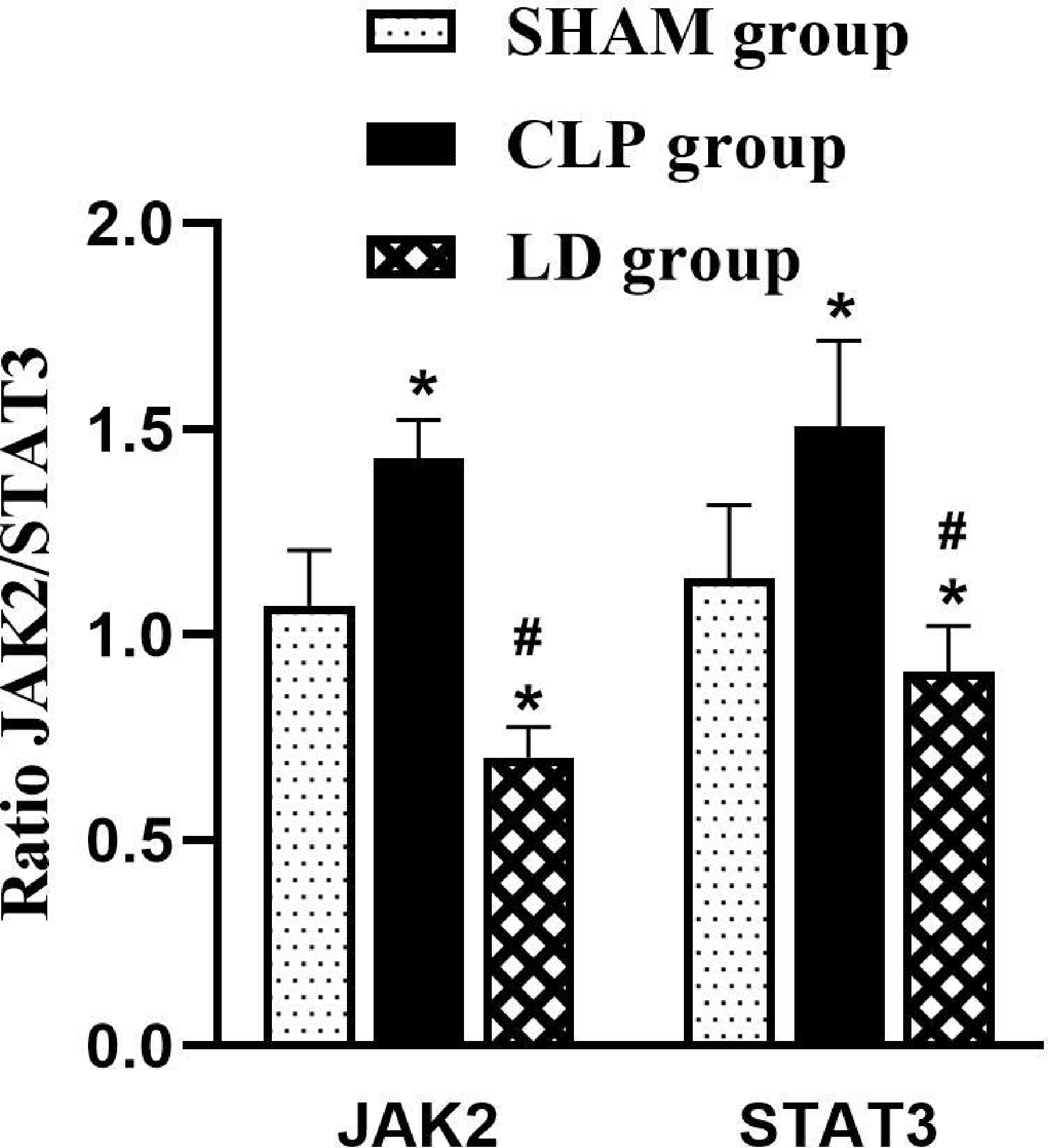

Supplement: S2 Raw Images — (ZIP) [file pone.0322653.s002.zip › S1_raw_images2-wb data/picture/wb 1.tif]

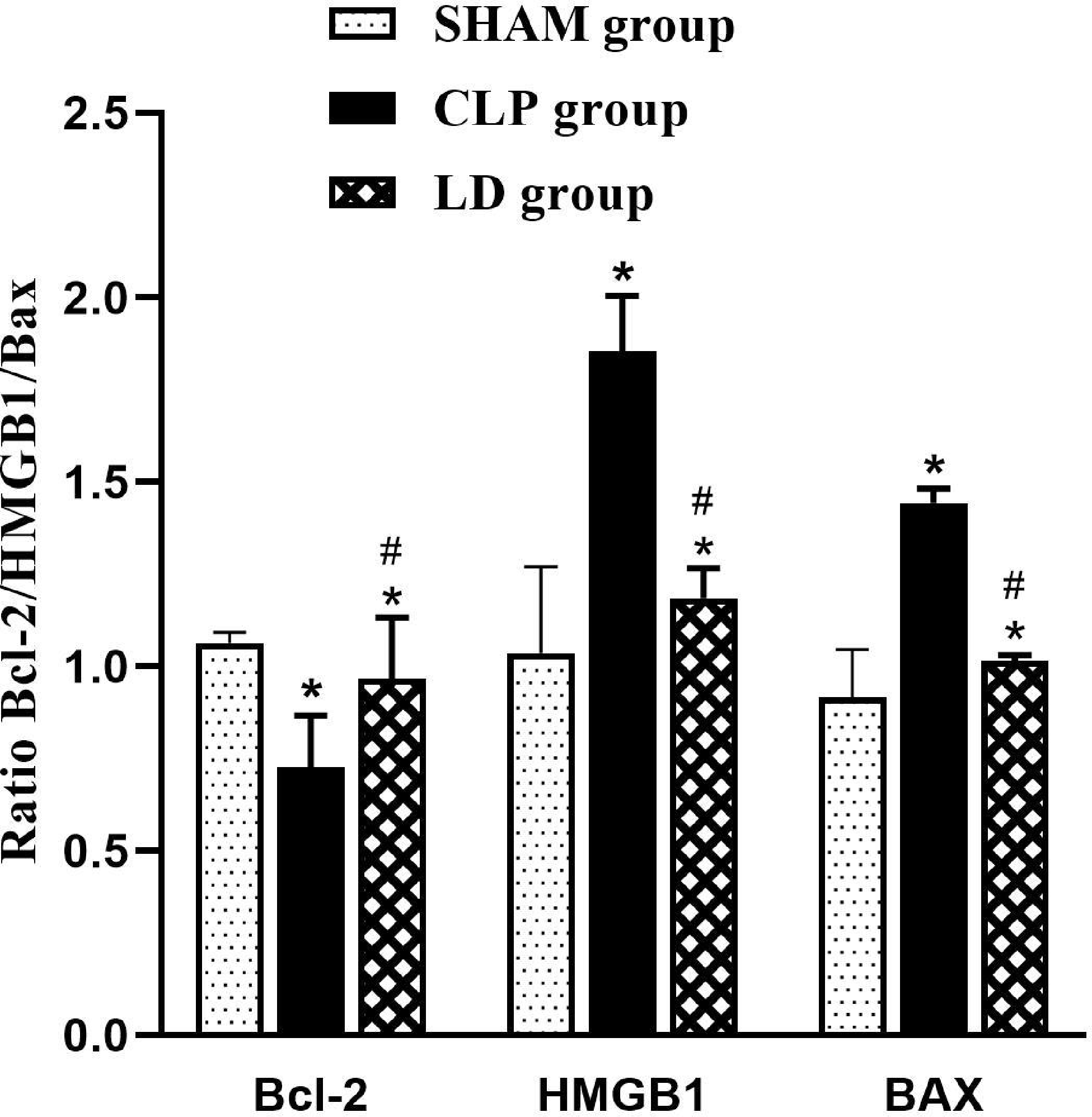

Supplement: S2 Raw Images — (ZIP) [file pone.0322653.s002.zip › S1_raw_images2-wb data/picture/wb4.tif]
