# Supplementary material for: The effect of lidocaine against sepsis-induced acute lung injury in a mouse model through the JAK2/STAT3 pathway
Source: PLoS One. 2025 May 8;20(5):e0322653. doi: 10.1371/journal.pone.0322653 (PMC12061136; doi:10.1371/journal.pone.0322653)
Supplement: Attachment — Submitted filename: 3-Response to Reviewers.docx [file pone.0322653.s004.docx]

**Response to Reviewers**

Firstly, I would like to express my gratitude for your attention and professional dedication to my research work. I appreciate and value your suggestions, and I have taken them seriously, making reasonable modifications. I am now returning the revised manuscript to you with the changes highlighted in red for your further review. Simultaneously, I have also uploaded the link to the raw data（https://www.jianguoyun.com/p/Dfz4doAQwOfCDBjTu-MFIAA） for the editor and peer reviewers to examine. Additionally, I will actively answer any questions you may raise, and if there are any inadequacies, please feel free to point them out at any time. Lastly, I would like to thank you once again for your valuable advice and suggestions, and I look forward to further cooperation with you. I am committed to continuously improving the quality of this paper. Below are my responses to and changes based on your comments.

**Reviewer #1**

1.A schematic diagram of the experimental design has been completed and added to the text.

2.The animals in this experiment were euthanized using inhalant anesthetics, and this has been added to the experimental methods.

3.The potential mechanisms by which lidocaine may affect signaling pathways related to inflammation and apoptosis are further mentioned in the discussion. Additionally, content related to the translational potential of these findings in the clinical setting has also been added.

4.I have carefully corrected the grammatical errors you mentioned.

**Reviewer #2**

1.This revision provides a more detailed description of how this pathway precisely mediates its anti-inflammatory effects.

2.In the discussion section, content comparing other anti-inflammatory drugs with lidocaine has been added, along with relevant literature citations.

3.The discussion section has been expanded to introduce the limitations of this study and to propose and design the next steps in the research.

4.The connection between this experiment and clinical practice has been further elaborated, and some solutions to the side effects of lidocaine have been introduced through literature. The next experiment will further study the long-term effects of lidocaine on septic mice.

5.The conclusion section has undergone significant revisions, providing a detailed summary of the research conclusions of this experiment, and the next steps in the research have also been mentioned.
